# Lipid Architecture in the Zika Virus

**DOI:** 10.1021/acsphyschemau.5c00113

**Published:** 2026-02-27

**Authors:** Camila Assis Tavares, Martín Soñora, Sergio Pantano, Leandro Martínez

**Affiliations:** † Institute of Chemistry and Center for Computational Engineering and Science, Universidade Estadual de Campinas (UNICAMP), Campinas, SP 13085-015, Brazil; ‡ 123939 Institut Pasteur de Montevideo, Montevideo 11400, Uruguay

**Keywords:** Zika virus, lipid bilayer, viral structure, coordination number, molecular simulations, distribution functions, soft matter

## Abstract

Far from being passive barriers, viral membranes actively influence the mechanical properties and biological activity of viruses. Their lipid–protein composition forms a responsive interface that impacts how viruses assemble, remain stable, and interact with their surroundings. While the behavior of lipids and proteins in cellular membranes is well-described, their specific contributions within viral systems remain underexplored, largely due to nanoscale complexity and experimental limitations involved. Here, coarse-grained molecular dynamics simulations of Zika virus were analyzed to characterize lipid organization inside the virus. Lipid selectivity is strongly influenced by helix residue composition and depth of insertion into the membrane. The amphipathic EH-3 helix, for instance, preferentially coordinates with POPC but also establishes localized contacts with POPS through Lys and Ser residues, reflecting a balance between hydrophobic and electrostatic interactions. In contrast, the transmembrane ET-2 helix, dominated by hydrophobic residues, displays reduced lipid selectivity, with only peripheral serines showing a modest preference for POPS. Across the viral envelope, POPE contributes less to residue-specific coordination, while POPS participates in polar interactions that modulate the environment near positively charged residues. By shedding light on how lipids contribute to the architecture of the viral envelope and membrane, this work offers insights that deepen the understanding of viral particle integrity, guiding target membrane-dependent processes in antiviral design.

## Graphical abstract

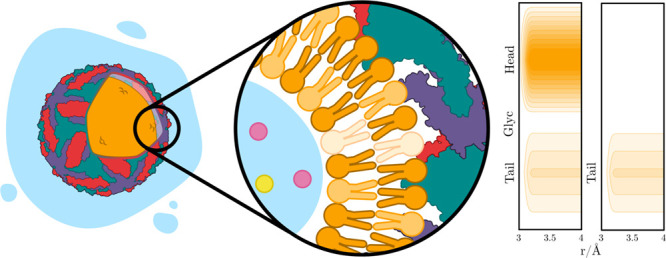

## Introduction

The Flaviviridae virus family, composed of some well-known viruses, such as Zika (ZIKV), dengue, Japanese encephalitis, West Nile, and tick-borne encephalitis viruses, is of great relevance to human health.^1^ From the yellow fever outbreaks in the 17th century to the dengue fever endemic scenario in tropical countries, the number of people affected by these viruses has risen significantly.^2–4^ The Zika virus outbreak in Brazil during 2015–2016, for instance, led to an estimated 440 thousand to 1.3 million infections. While most cases are mild or asymptomatic, the epidemic raised alarm due to Zika’s sexual transmission even months after infection^5^ and a sharp intensification in microcephaly cases among neonates born to mothers infected during pregnancy (particularly when infection occurred in the first trimester). The presence of congenital Zika syndrome significantly increased child mortality, with affected individuals experiencing a death rate of 52.6 per 1000 person-years.^6^ In adults, infection was also associated with an elevated incidence of Guillain–Barré syndrome.^7^

Despite existing control measures and vaccines, flavivirus transmission persists due the high costs of rapid diagnosis tests, unsustainable vector control, incomplete vaccine protection, and zoonotic reservoirs, highlighting the urgent need for better tools to combat these viruses.^8–13^ Thus, the importance of understanding and combating flaviviruses is a crucial priority for global health initiatives.

Flaviviruses are spherical particles approximately 50 nm in diameter, containing a positive single-stranded RNA genome of around 11,000 bases.^14,15^ This genome encodes both structural and nonstructural proteins. The mature Zika virus is composed of three main structural proteins: Capsid (C), Membrane (M), and Envelope (E), organized into a shell-like structure that encloses a host-derived lipid bilayer.^16–20^ Meanwhile, seven nonstructural proteins (NS1, NS2A, NS2B, NS3, NS4A, NS4B, and NS5) play crucial roles in viral replication, assembly, and immune evasion.^21,22^

The outer protein layer of Zika virus consists of 90 E−M heterodimer pairs (homodimers) arranged in a metastable herringbone pattern on the viral surface, where three such dimers within each icosahedral asymmetric unit are nearly parallel to one another (shown in Figure 1a, chains K, M, and O in red, violet, and green, respectively). The E protein plays a central role in this organization and is composed of three structural domains (I, II, and III depicted in red, yellow, and blue, respectively), which form a predominantly β-sheet-rich surface (Figures 1b,c). Besides these three domains, the E protein is also composed of the stem and transmembrane subunits (green), which anchor the E protein to the lipid bilayer (Figure 1c). On the other hand, the M protein (dark blue) is formed mainly by α-helices, represented in Figure 1c.^23–26^ In their mature form, these structural elements are arranged into a highly organized, quasi-perfect icosahedral shell, composed of 180 E and M protein copies.^27^

**Figure 1:**
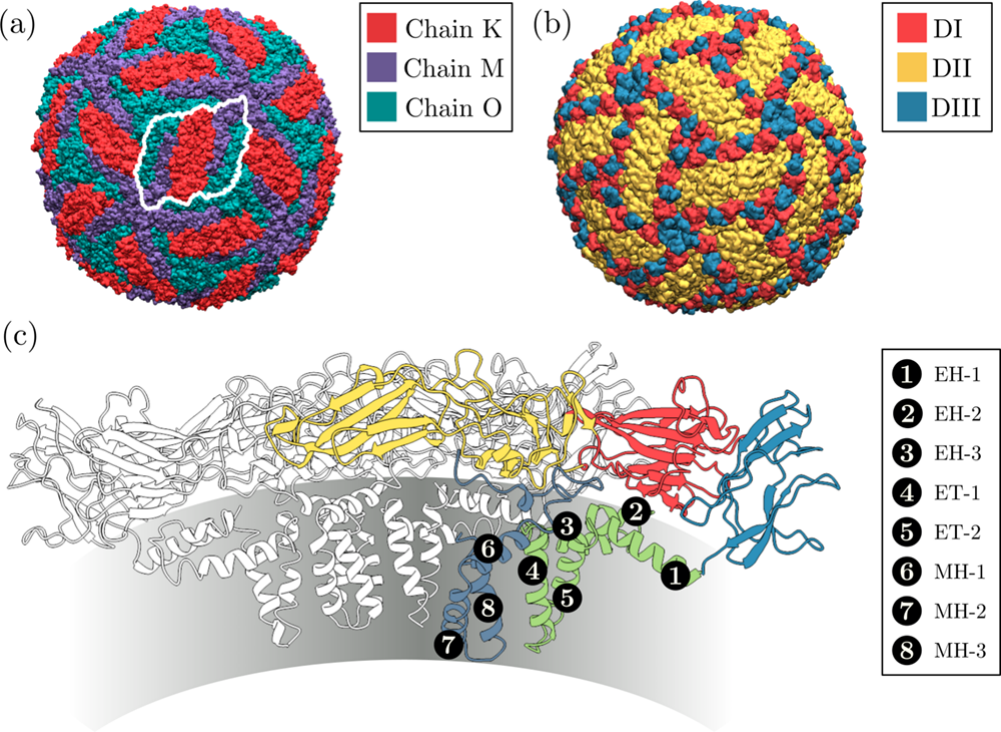
Structural organization of the Zika virus protein layer: (a) surface view displaying the characteristic herringbone pattern formed by E-protein dimers, highlighting the E–M raft (the three parallel dimers); (b) domain architecture of the E protein, showing domains I, II, and III; (c) side view of the E–M heterodimer, illustrating the E protein with its stem (EH-1–EH-3) and transmembrane regions (ET-1–ET-2), and the M protein (MH-1–MH-3). The lipid bilayer is depicted as a gray gradient band.

Another essential part of the viral structure is the lipid bilayer of flaviviruses, including ZIKV, which is derived from host cell membranes, primarily the endoplasmic reticulum (ER) and the trans-Golgi network.^28^ During assembly, the capsid protein packages the viral RNA to form the nucleocapsid, which associates with the premembrane (prM) and E proteins embedded in the ER membrane. This interaction induces budding into the ER lumen, encapsulating the nucleocapsid within a host-derived membrane enriched mainly in phosphatidylcholine (POPC), phosphatidylserine (POPS), and phosphatidylethanolamine (POPE).^29,30^ The immature virion is transported through the Golgi, where cellular furin cleaves prM into Pr and M fragments. In the acidic Golgi environment, Pr remains bound to the particle and prevents premature (futile) fusion; upon release into the neutral extracellular milieu, pr dissociates, promoting the formation of the mature, infectious virion.^31^

This lipid membrane is not merely a scaffold but an active determinant of viral function, shaped by selective phospholipid enrichment and leaflet-asymmetric dynamics.^32^ Beyond contributing to local stress accumulation, the intrinsic shapes of constituent lipids and transmembrane charge asymmetry promote membrane curvature and create conditions favorable for fusion.^33–35^ Furthermore, studies have shown that variations in the interactions between lipids and nonstructural proteins (NS1) across distinct ZIKV genotypes may contribute to differences in virulence.^36,37^ Lipidomic analysis revealed a predominance of zwitterionic phospholipids (mainly POPC and POPE), with a smaller anionic fraction (≈6–8 mol %) corresponding to POPS. This distribution closely mirrors that of the mammalian endoplasmic reticulum membrane, as reported previously.^38,39^ Guided by these data, model liposomes mimicking this composition at a 6:3:1 molar ratio of POPC:POPE:POPS were prepared.^40^

Experimental techniques such as X-ray crystallography and cryo-electron microscopy have provided high-resolution structures for multiple flaviviruses, detailing the organization of proteins and nucleic acids.^23,41–44^ However, these methods generally fall short of resolving atomic-level features of low-electron-density components (water, counterions, and other small molecules) that are critical for understanding viral stability.^45^ This limitation reflects the large size and complexity of the assemblies (often exceeding one million particles), as well as the high mobility, partial occupancy, and structural disorder of these constituents within the virion architecture.^46–48^

The main goal of this study is to delineate the molecular architecture of Zika virus, with emphasis on the spatial organization of its lipid bilayer. Leveraging large-scale coarse-grained molecular dynamics of the intact virion, combined with minimum-distance distribution analyses, lipid enrichment and bilayer lipid distribution are quantified, providing a detailed picture of lipid distribution in a whole Zika virus particle, which is not easily observable through traditional experimental techniques. This study enhances our understanding of the molecular interactions of viral components, offering new insights into the function and structure of the viral membrane.

## Methods

### Zika Virus Molecular Dynamics Simulations

Coarse-grained simulation of Zika virus was performed based on the high-resolution structure of the mature virion (PDB code: 6CO8).^23,49^ The model virion has an approximate radius of 210 Å, with 180 E–M complexes arranged according to the experimentally derived icosahedral symmetry. The system was solvated through a multiscale configuration, using the SIRAH Force Field version 2.0, as shown in Figure 2.^50–53^ SIRAH is a coarse-grained, self-consistent molecular dynamics force field that employs a residue-based classical Hamiltonian with optimized bonded and nonbonded parameters to reproduce solvation, hydrophobic/hydrophilic balance, and long-range electrostatics across biomolecular systems, while enabling multiscale coupling with atomistic models. A solvation shell of coarse-grained WT4 water with ions was placed directly adjacent to the protein–membrane interface on both the luminal (virion cavity) and extraviral sides, while the remaining bulk solvent (inside the cavity and in the external medium) was represented by the supramolecular WLS model.^54,55^ The WLS model is useful for representing bulk water with reduced computational cost in systems where the diffusion of components across the solvent is not of interest.^55^ For simplicity, the intravirion WLS is not shown in Figure 2. The viral envelope was modeled as a symmetric lipid bilayer composed of POPC, POPE, and POPS in a 6:3:1 ratio, distributed between the inner and outer leaflets. System building was performed with PACKMOL,^49,56,57^ and MD simulations were conducted with GROMACS (version 2018.4, http://www.gromacs.org).^50,51,53–59^ The full model contains 1,297,988 CG beads, of which 489,960 comprise the viral proteins and 135,240 are lipid CG beads. A temperature of 300 K was maintained using a V-rescale thermostat,^60^ while pressure was kept at 1 bar using a Parrinello–Rahman barostat.^61,62^ The minimum cutoff for nonbonded interactions was set at 12 Å, and long-range electrostatics were evaluated using particle mesh Ewald.^63,64^ Integration steps and neighbor searching occurred every 10 steps. The equations of motion were solved using a leapfrog integration algorithm^65^ with a time step of 2 fs for the first nanosecond and then switched to 20 fs. The details of the construction and equilibration of the system, and the validation of the lipid membrane model, are described in Soñora et al.^49^ Here, snapshots were taken every 100 ps for analysis from a 2 μs production simulation.

**Figure 2:**
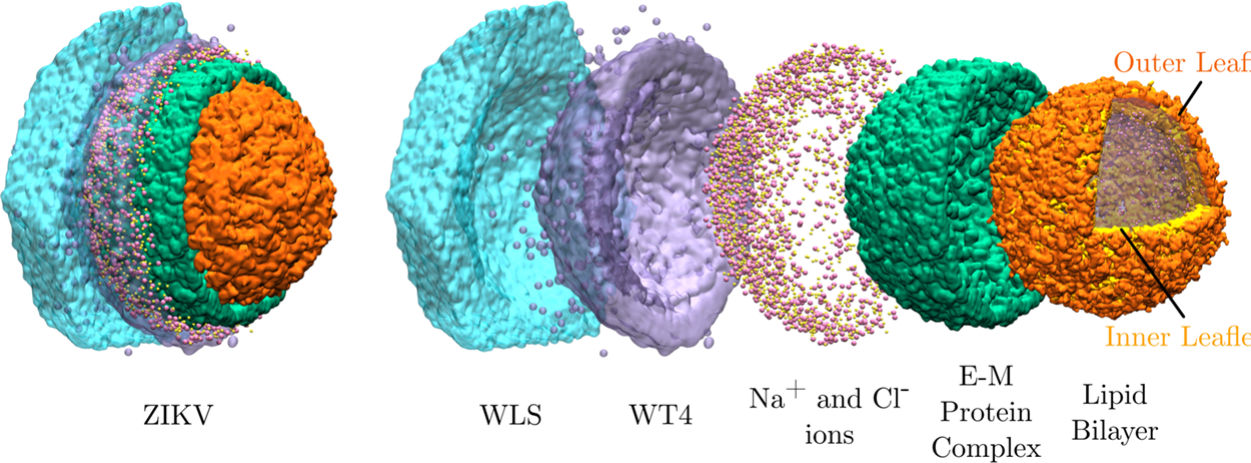
Simulated Zika virus model. The figure shows the system divided into layers: water model WLS, water model WT4, ions Na^+^ and Cl^–^, the E–M protein complex, and the lipid bilayer. The bilayer is highlighted with its outer (orange) and inner (yellow) leaflets, illustrating the organization of the viral membrane. Finally, the core of the virus is filled with layers of WT4 and WLS water models (not shown). The full model contains ∼1.3 million CG beads.

### Light-Material Distribution Analysis

The spatial organization of light material in the viral system was investigated by analyzing coordination numbers (CNs). Because of the complex shapes of the proteins and lipids, the CNs were computed from *minimum-distance* counts between the lipid bilayer and viral proteins along the molecular dynamics simulation trajectories. These analyses were carried out using ComplexMixtures.jl (https://m3g.github.io/ComplexMixtures.jl), an open-source package implemented in Julia,^66^ designed to quantify solute–solvent interactions in systems with complex geometries.^46,67^

ComplexMixtures.jl allows the computation of minimum-distance distribution functions (MDDFs) and Kirkwood–Buff integrals (KBIs), offering a detailed view of solute–solvent distributions.^46^ In a nutshell, MDDFs represent the distribution of the shortest distances between solute and solvent atoms. Because minimum distances implicitly take the shapes of the molecules into account, they are particularly useful for studying macromolecular solvation.^67^

These distributions can further be decomposed into the contributions of individual atoms or groups of atoms to the minimum-distance count, offering detailed insights into the chemical nature of the interactions.^46^ While beyond the purpose of the current work, MDDFs can also be used to compute KBIs, linking the microscopic picture of solvation to macroscopic thermodynamic parameters, like the relative stability of different states of the solutes in the presence or absence of a cosolvent.^67^ The structural complexity of the virus architecture makes it difficult to normalize MDDFs. In this case, therefore, coordination numbers are the most useful parameters for providing insights into the molecular arrangement of solvent molecules in the vicinity of the proteins.^46,67^ Minimum-distance coordination numbers can also be resolved per atom group or residue, offering a rich view of the chemical and topological determinants of molecular recognition and solvation in complex, crowded biological environments.^46,67^

Minimum-distance CNs measure the number of solvent molecules surrounding a solute within a specified radial cutoff. For each simulation frame, CN values were obtained by counting the number of solvent molecules (e.g., lipids) with at least one atom (or CG bead, in this case) located within a distance *r*_*c*_ from the solute atoms (e.g., proteins), as illustrated in Figure 3.^46,67^

**Figure 3:**
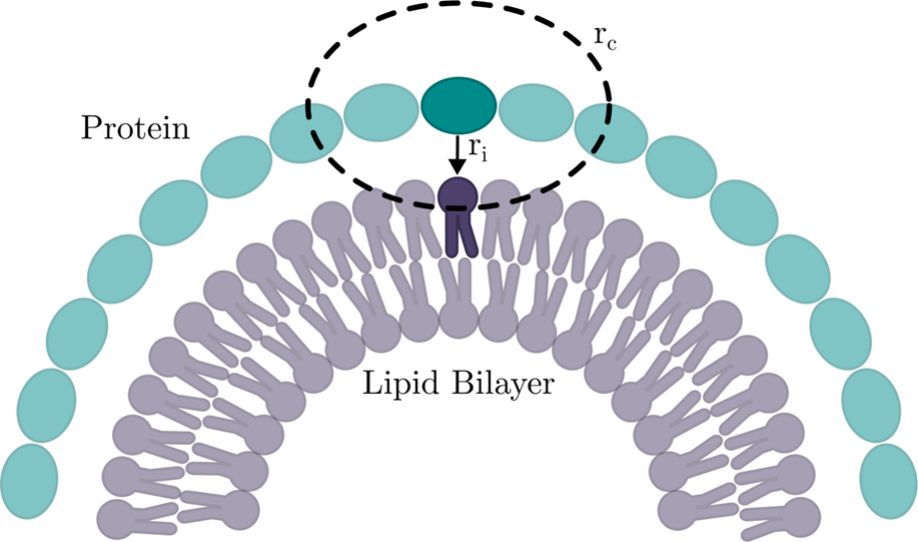
Schematic representation of the minimum distance *r*_*i*_ between a lipid molecule in the outer monolayer (purple) and a protein chain (teal). This distance is used to compute the coordination number, where lipid molecules with at least one atom within a defined cutoff radius (*r*_*c*_) contribute to the sum.

Mathematically, the CN can be expressed as eq 1.

$$\mathrm{CN}=\sum_{i=1}^{N_{\mathrm{LM}}}\Theta (r_{i}-r_{c})$$ 1

Where *r*_*i*_ is the *minimum distance* between any atom of the solute molecule and any atom of each solvent molecule *i*, *r*_*c*_ is the cutoff radius, and Θ is the Heaviside function (here defined as 1 if *r*_*i*_ ≤ *r*_*c*_, 0 otherwise). The sum covers all light-material molecules (*N*_LM_) within the cutoff.^46^ The fast computation of these cutoff-delimited properties is possible using cell lists, implemented here in the CellListMap.jl (https://m3g.github.io/CellListMap.jl) library.^68^ Special handling of SIRAH particles and residue types was implemented in PDBTools.jl (https://m3g.github.io/PDBTools.jl) for customizable and flexible analysis of coordination numbers and contributions.

This approach provides a robust and interpretable metric for assessing the spatial distribution of lipids and other light components around viral proteins. Examining CNs across time and different protein regions provides microscopic insights into interaction patterns that contribute to viral structure and assembly.

### Helical Wheel Projection

The correlation between helix polarity and lipid distribution was analyzed using helical wheel projections, which provide a two-dimensional representation of the α-helical geometry. In this projection, residues are positioned sequentially along a circle, assuming an angular displacement of 100° per residue, consistent with the periodicity of the α-helix. To accommodate longer sequences, the radius of the helix is incrementally increased after every 18 residues, preventing overlap between consecutive turns.^69^

The residue-specific contributions to the lipid coordination numbers were obtained with the *ResidueContributions* function of ComplexMixtures.jl. At a 5 Å cutoff distance, the contributions were used to define a color gradient, with optional normalization to the interval [0, 1], ensuring comparability across different systems or distance thresholds.

To complement this visualization, each amino acid was assigned a hydrophobicity value according to the Fauchère and Pliska scale.^70^ These values were then used to calculate the hydrophobic moment vector, which represents the amphipathic character of the sequence. The vector is obtained as the weighted average of hydrophobicity contributions along the x and y components of the helix. For interpretability, the entire projection is rotated such that the hydrophobic moment vector is oriented vertically, highlighting the separation between hydrophobic and hydrophilic residues.

## Results and Discussion

The following analyses will focus on the interactions between the lipid bilayer and the protein complex (E and M proteins, Figure 1). Here, an interaction refers strictly to a coordination event at 5 Å, a measure of local contact density, without carrying any implication of a defined chemical bond. Three distinct types of lipids representing phospholipid families with varying physicochemical characteristics are present in the membrane: POPC, POPE, and POPS, with a proportion ratio of 6:3:1. The coarse-grained representation of these lipids is shown in Figure 4 and will be important for the description of the contributions of each chemical group to the interactions with the proteins.

**Figure 4:**
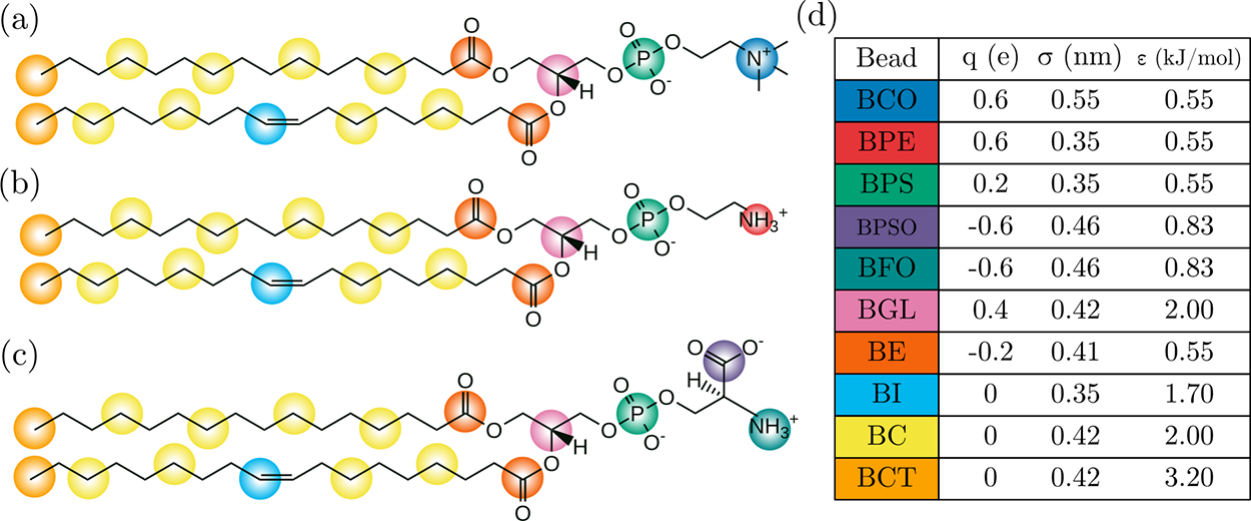
Coarse-grained (CG) representation of phospholipids used in the simulations.^50,51,53^ Chemical structures of (a) POPC, (b) POPE, and (c) POPS are shown with their respective CG beads colored by chemical type. Each bead corresponds to a specific portion within the lipid structure, including headgroups, the glycerol moiety, and acyl tails. (d) Table of nonbonded parameters for each bead type, listing partial charge, Lennard–Jones radius, and well depth, as used in the SIRAH force field.^50^

### Structural Overview of the Viral Envelope and Lipid Coordination

Figure 5 shows the structure of the Zika virus proteins colored according to the average POPC coordination numbers obtained from the molecular dynamics simulations. The colors are scaled contributions of POPC to chains of types K and L and represent relative POPC coordination levels rather than absolute counts, providing a spatial profile of lipid–protein interactions along the heterodimer. The most intense contributions are observed in α-helices, which act in membrane anchoring or stabilization, as expected.

**Figure 5:**
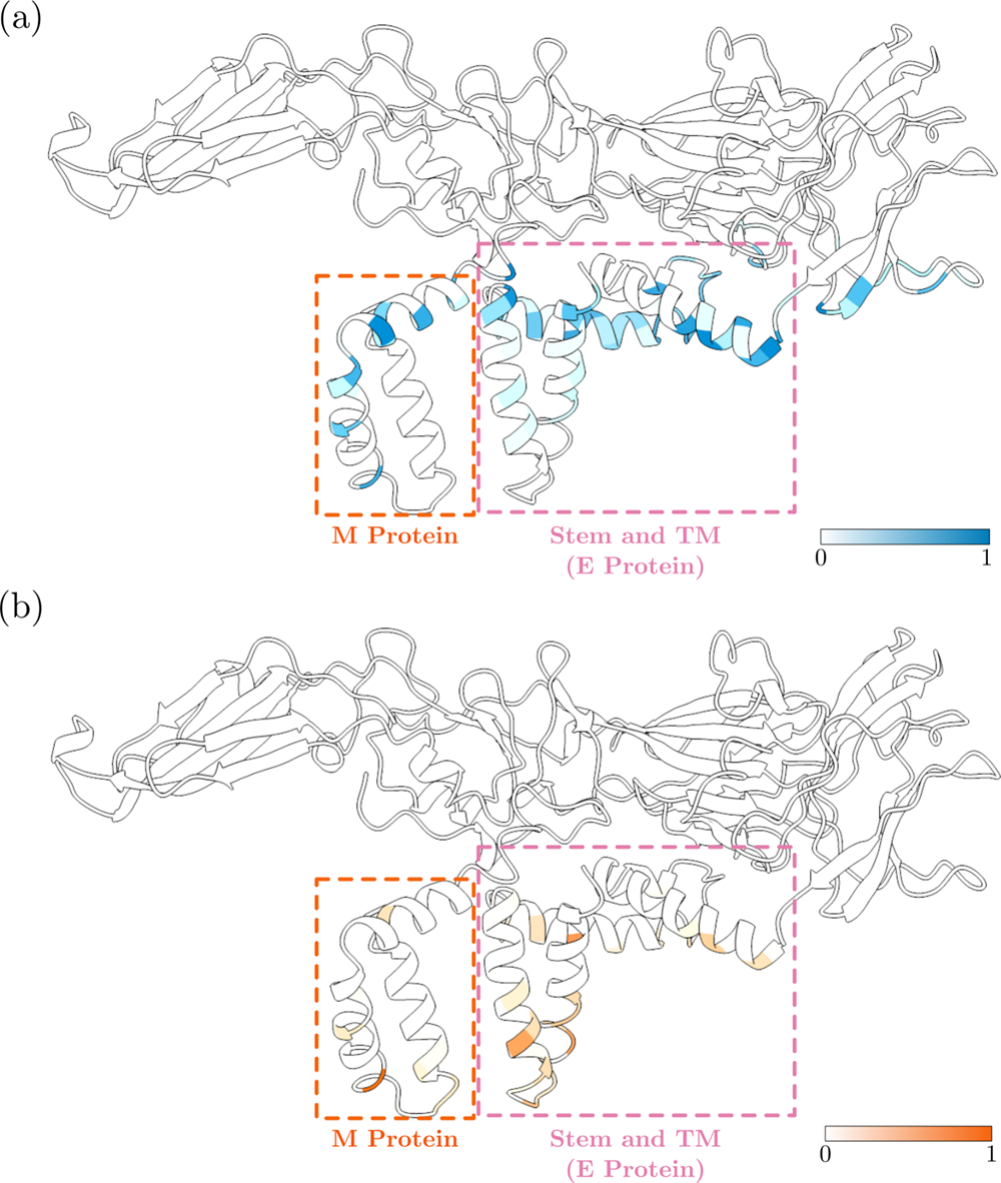
POPC interactions with the proteins of the E–M complex: interactions with lipids of the (a) outer and (b) inner leaflets of the membrane. Darker regions imply more frequent contacts with lipids. Similar figures for POPE and POPS are available in Figure S1 of Supporting Information (SI).

The interaction patterns are, at first sight, similar for the three lipid types (Figure S1). The heterogeneous distribution of interactions occurs because membrane association is localized to specific structural domains.

The observation that lipids from the inner leaflet interact with the amphipathic helices, while those from the outer leaflet contact the inner regions of the transmembrane helices, suggests an active lipid flip-flop process across the bilayer. This dynamic exchange between leaflets contributes to the asymmetric profile of the distribution of phospholipids, likely facilitating the continuous reorganization of lipid–protein contacts required to maintain the curvature and stability of the viral envelope.

Figure 6 displays the viral shell projected onto a plane as Hammer–Aitoff projections for E proteins (Figures 6a,c,e) and M proteins (Figures 6b,d,f). The density of lipids in each region is illustrated. Regions of greater density correspond to insertions into the lipid membrane. POPC molecules are arranged around the symmetry axis of the chains of E proteins (indicated by the oval marker for the 2-fold axis, the triangle marker for the 3-fold axis, and the pentagon marker for the 5-fold axis, in the insets of Figures 6a,c,e), forming clustered and patchy interaction patterns. Areas of higher and continuous intensity indicate strong and recurrent POPC enrichment. Notable clustered and patchy patterns are also observed in the M protein, associated with specific affinity for phospholipids. Figures S2 and S3 show the distributions of POPE and POPS across each chain.

**Figure 6:**
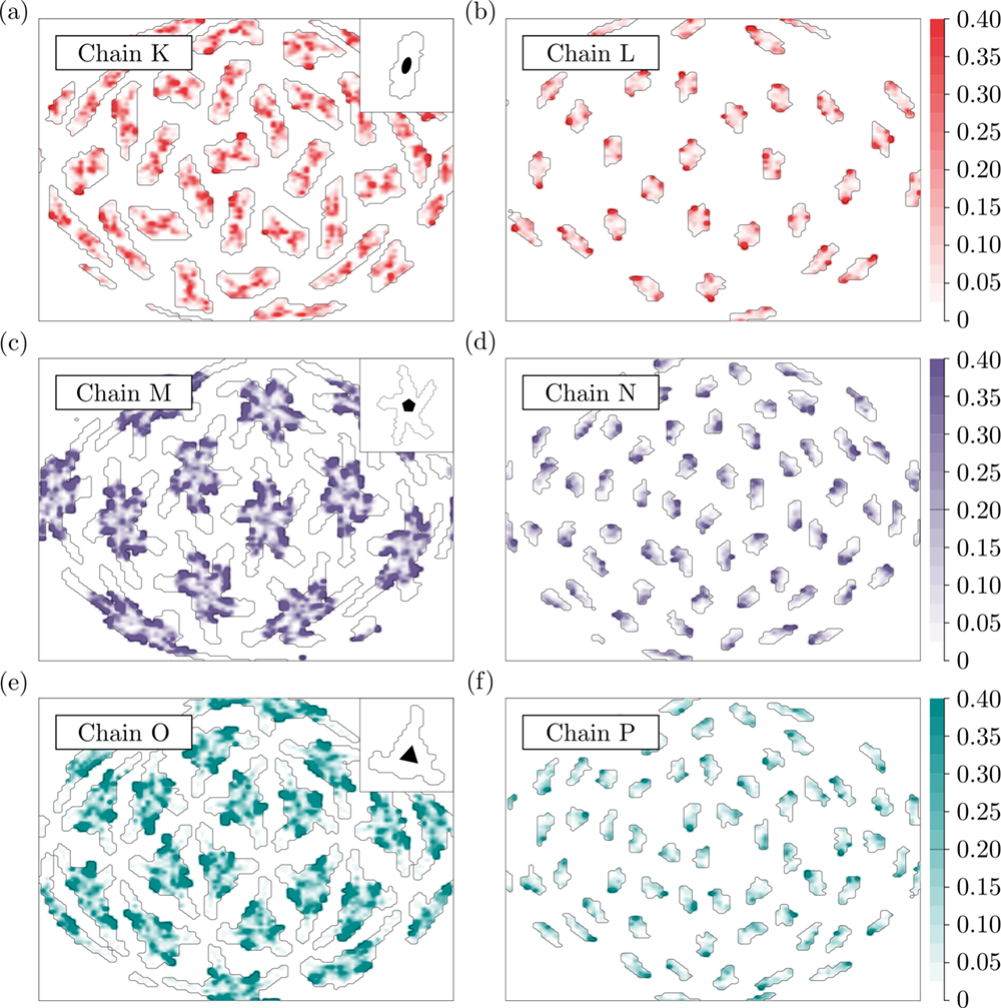
Overview of the heterogeneity in POPC contributions to envelope and membrane protein: chains (a) K, (b) L, (c) M, (d) N, (e) O, and (f) P. The edges of each chain are outlined with black lines, and geometric shapes represent each one of the symmetric axes: 2f (●), 3f (▲), and 5f (⬟). Similar figures for POPE and POPS distributions are available as Supplementary Figures S2 and S3.

Figure 7 presents the coordination numbers of the phospholipids as a function of the distance from the surface of the E–M protein complex, with contributions of different types of residues of the proteins indicated.

**Figure 7:**
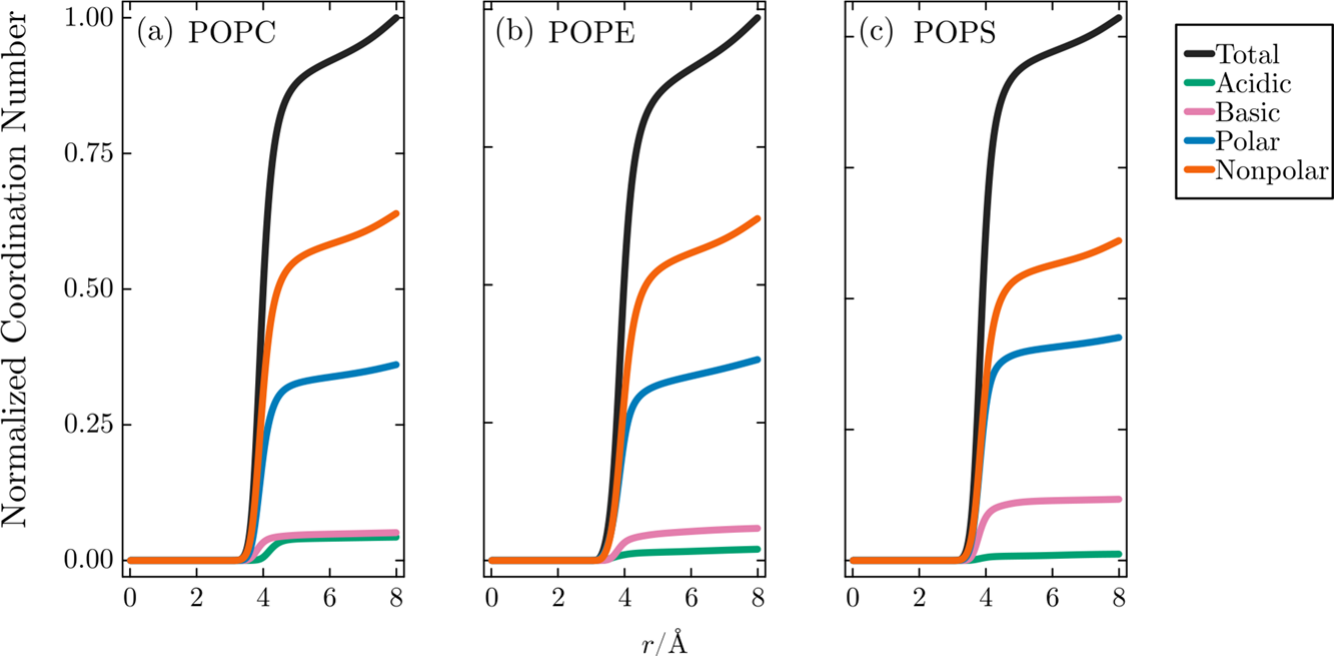
Coordination numbers of (a) POPC, (b) POPE, and (c) POPS across the six chains (K, L, M, N, O, and P), normalized by total coordination at 8 Å. Contributions of residues of distinct chemical properties (acidic, basic, polar, and nonpolar) are shown. *r* denotes the minimum distance, in angstroms, between any protein atom and the atoms of lipids.

As expected, phospholipids exhibit higher coordination with nonpolar residues because of the hydrophobic nature of phospholipid tails. Interactions with polar and charged residues are less frequent, with basic residues (positively charged) showing slightly higher coordination than acidic residues (negatively charged) due to electrostatic repulsion from the negatively charged phosphate groups of the phospholipids. These comparisons reflect the number of contacts and do not account for normalization by residue abundance.

Furthermore, POPC exhibited more frequent interactions with apolar residues per lipid, compared to POPE and POPS, consistent with its more hydrophobic and less polar character. The coordination numbers observed for basic residues across all chains might arise primarily from interactions with the phosphate groups of the lipids. Stronger interactions of basic residues with POPS were observed because of its negatively charged headgroup. Acidic residues displayed higher coordination with POPC around 4.5 Å, which can be attributed to the larger choline headgroup available for interactions.

To provide a global overview of lipid–protein interactions, the fraction of each phospholipid species located within 5 Å of the protein in the viral envelope was quantified (Table 1). In both inner and outer monolayers, POPC accounts for the majority of contacts (60.4 and 60.6%, respectively), which is consistent with its higher molar ratio in the membrane composition. POPE and POPS follow with progressively lower values, reflecting their relative abundance.

**Table 1:** Fraction of Phospholipids in the Inner and Outer Monolayers That Are Found within 5 Å of Any Protein Surface^a^

	**phospholipids**	**fraction of lipids that interact with the proteins** (%)
inner monolayer	POPC	60.14 ± 0.41
	POPE	28.57 ± 0.40
	POPS	11.29 ± 0.22
outer monolayer	POPC	60.52 ± 0.03
	POPE	29.28 ± 0.03
	POPS	10.21 ± 0.02

a The time dependence of the composition of the lipid layers is shown in Supplementary Figure S37. Errors correspond to the standard error of the mean considering the number of uncorrelated samples (Supplementary Figures S39–S44).

These patterns of interaction, although largely dictated by lipid composition, are not entirely uniform across the viral envelope. Even though the ZIKV envelope does not follow Caspar and Klug quasi-equivalence in the strict sense, the icosahedral organization implies that equivalent copies of E–M complexes can experience slightly different local environments depending on their position within the herringbone pattern, leading to subtle variations in lipid coordination.^71^ This principle also extends to membrane-associated regions, where the same transmembrane helices or amphipathic segments may be embedded in subtly distinct lipid environments, depending on their spatial position within the icosahedral shell and on the heterogeneous envelope thickness and curvature characterized for this model in our companion study ‘*The stressed life of a lipid in the Zika virus membrane*’.^33^ Indeed, the substructure interaction patterns computed here, particularly within the transmembrane segments of the E and M proteins, reveal small but consistent variations in lipid coordination, reinforcing this concept of context-dependent interaction landscapes. This will be addressed in the next section.

### Spatial Distribution of Lipids and Local Preferences

In this section, the spatial distribution of phospholipids surrounding all protein chains of the Zika virus was analyzed, with a particular focus on how these lipids are organized relative to the viral envelope residues. This analysis highlights patterns of lipid arrangement and enrichment across different regions of the protein surface, providing insights into preferential lipid–protein interactions.

Figure 8 displays density maps showing the weighted differences in coordination numbers between lipid pairs. The weights correspond to the proportion of the lipids in the membranes; thus, the differences shown are associated with the relative affinities of each type of lipid to the corresponding residue. In Figure 8a, for instance, positive values (blue) indicate higher affinity of the residue for POPC, whereas negative values (orange) indicate higher affinity for POPE. The figure focuses on the TM region (residues Val473–Ile484) of chain K, and on region MH-3 (residues Ala50–Leu64) of chain L. These regions were selected because significant differences in lipid affinities were observed along the chains.

**Figure 8:**
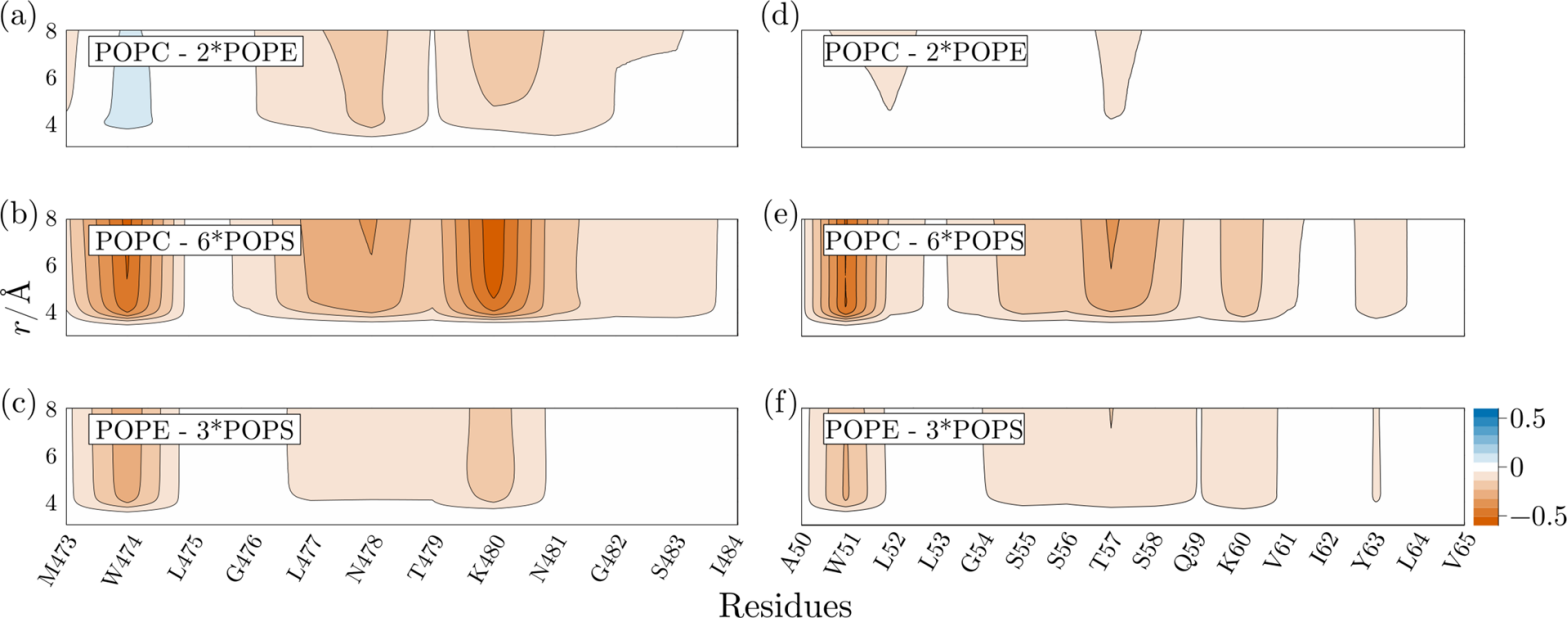
Density maps showing the weighted differences in coordination numbers between lipid pairs within residues Met473–Ile484 of the TM region in chain K: (a) POPC and POPE, (b) POPC and POPS, and (c) POPE and POPS, and the relative differences in the lipid interactions among phospholipids at residues Ala50–Val65 of the M protein in chain L: (d) POPC versus POPE, (e) POPC versus POPS, and (f) POPE versus POPS. The parameter *r* represents the minimum distance (Å) between any protein atom and the nearest lipid atom. The coefficients in the labels represent weighting factors based on the 6:3:1 lipid ratio (POPC:POPE:POPS) used in the simulation.

Figure 8a shows POPE exhibiting slightly higher affinity (negative weighted difference) compared to POPC in the TM region selected, except for residue Trp474. Similarly, Figure 8b,c shows the greater affinity of POPS relative to POPC and POPE. The greater affinity of POPS to this region can be traced down to the interactions of POPS with Asn478 and Lys480, as well as to Trp474. The stronger interactions with the polar residues are a consequence of the greater hydrophilicity of the polar head of POPS relative to the other lipids. On the other side, the interaction with Trp474 results from an orientational anchoring role that this tryptophan plays, where its pyrrole moiety points toward the more hydrophilic part of the lipid bilayer. Moreover, this coordination number is also associated with the nearby presence of residue Lys480.

Similar patterns are also observed in Figure 8d–f, which analyze chain L of the M protein. Preferential interactions with POPS are induced by interactions with Lys60 and the hydrogen bonds with Thr57 and Ser55. Notably, Trp51 also interacts with greater affinity with POPS than with the other lipids, due to its proximity to the Lys60 residue of the dimer vicinal parallel chain that composes the dimer.

These preferential interactions can be attributed to the unique properties of POPS, which, unlike other phospholipids, carries a net negative charge. This characteristic enhances its ability to interact with polar molecules and positively charged residues. In comparison, POPE and POPC exhibit similar but progressively smaller affinities due to their differing chemical structures, with POPE demonstrating moderate coordination numbers and POPC being the lipid with the most hydrophobic head.

Supplementary Figures S4–S20 show the differences in local affinities across all chains for the three phospholipids. For chains K, M, and O, the relative affinity of POPC and POPE was similar up to residue Thr321, except for chain O, where residues Lys215–Ile221 favored interactions with POPC. The stem and transmembrane regions of the E protein exhibited the most pronounced contributions of both lipids when compared to other subunits. The relative local affinities of POPC and POPS to chains K, M, and O showed a small preference for POPS up to residue Val341. Chains K, M, and O displayed a relevant deviation between each other at residues Val347–Pro354, with residue Asp348 contributing notably to POPC affinity. In the stem and transmembrane regions of E, POPC was generally favored, except for residues Ala473–Ser485, which displayed a stronger affinity for POPS. Finally, the comparison of local POPE and POPS affinities indicated that chain K prefers to interact with POPS, whereas chains M and O prefer POPE. Residues Trp429–Phe449 consistently showed POPE preference across all three chains, while residues Phe453–Ser485 favored POPS in all cases.

For chains of types L, N, and P, corresponding to the M protein, differences were observed primarily in the local affinities of POPC and POPE, and of POPC and POPS. Chain N exhibited a stronger affinity for POPC, in contrast to chains L and P, which displayed a preference for POPE. In chain N, residue Glu33 contributed markedly to this behavior. For the relative affinities of POPC and POPS, chain N again differed from the others, alternating the preference of these lipids throughout the sequence. It also maintained a strong contribution in Glu33 and residues within the Leu51–Leu63 region, favoring POPS over both POPC and POPE. In general, POPS displayed a consistent greater affinity to chains L, N, and P than POPE. Apart from these highlighted regions, differences in lipid affinities across residues were negligible.

Lysine residues 480 (in the envelope protein) and 60 (in the membrane protein) were selected for focused analysis based on prior reports indicating their role in clustering POPS near the luminal face of the TM helices,^49^ and a detailed analysis of their interactions with each chemical group of the lipids is shown in Figure 9. These residues, conserved across flaviviruses,^49^ are oriented toward the interior of the viral particle and have been implicated in mediating lipid–protein interactions that may contribute to the structural organization of the membrane.

**Figure 9:**
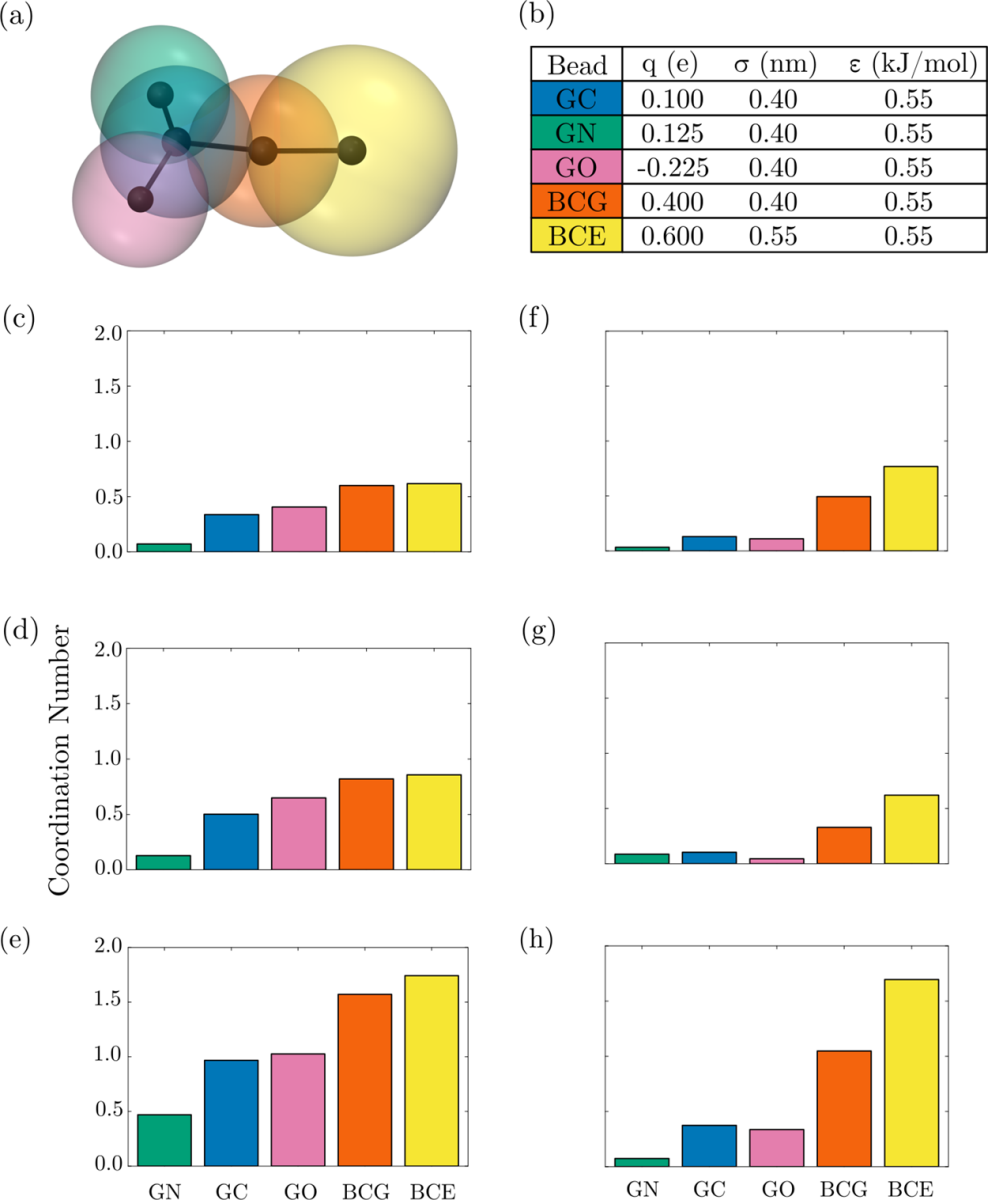
(a) Representation of a lysine residue with annotated SIRAH bead names and (b) Lennard–Jones parameters and partial charges for each CG bead. Bar plots show the normalized coordination number of (c) POPC, (d) POPE, and (e) POPS around residue Lys480 in chains of type K, and the respective distributions of these lipids around residue Lys60 in chains of type L (f–h). Normalization was performed by scaling POPE and POPS contributions by factors of 2 and 6, respectively, to match the lipid ratio of 6:3:1 (POPC:POPE:POPS).

Lys480 in chains of type K displayed patterns in lipid interactions similar to chains of types M and O. For POPC (Figure 9c), coordination is dominated by the BCE bead, representing the terminal amine group of lysine, consistent with strong electrostatic attraction between the positively charged side chain and the zwitterionic phosphate–choline headgroup. A similar pattern is observed for POPE (Figure 9d), though the interactions are slightly more balanced across GO and BCG, reflecting the smaller ethanolamine headgroup likely making additional contacts with the backbone beads. In contrast, POPS (Figure 9e) shows a distinct profile: the negatively charged serine headgroup of POPS strengthens interactions with the positively charged lysine beads (particularly BCE), and this effect propagates to neighboring beads.

The comparison with Lys60 in chain L further highlights the impact of helix positioning and the environment on lipid selectivity. For POPC (Figure 9f), BCE coordination dominates, but with lower overall values than observed for Lys480, suggesting a less exposed position in the membrane. With POPE (Figure 9g), coordination decreases even further across all beads, consistent with limited accessibility imposed by the smaller headgroup, when compared to that of POPC. Figure 9h emphasizes again the strong preference of BCE beads for anionic lipids (POPS), which influences the counts for other beads. These results show that lysine–lipid interactions are primarily driven by electrostatics at the BCE bead, modulated by headgroup size, charge, and the spatial context of the residue within the protein (similar profiles are observed for all chains in Figure S21).

### Selective Lipid Interactions at Protein–Membrane Interfaces - Part I

To gain further insight into lipid–protein interactions, this section focused on specific regions of the viral envelope proteins: the stem domain (E-H1, E-H2, and E-H3), the transmembrane helices (E-T1 and E-T2), and the membrane protein helices (M-H1, M-H2, and M-H3) (Figure 1c). These segments were selected for their structural relevance, as the E and M protein stems and transmembrane regions are directly involved in host cell entry and are highly exposed to the membrane. The analysis aimed to determine how phospholipid beads (Figure 2) coordinate with residues in these protein domains.

Figure 10 shows the contrasting interaction patterns of amphipathic and transmembrane helices with lipid heads and tails, as detailed in Figure 10a. In regions with strong amphipathic character compared to predominantly hydrophobic segments, distinct interaction patterns emerge in the head-to-tail interaction ratio between POPC and POPS.

**Figure 10:**
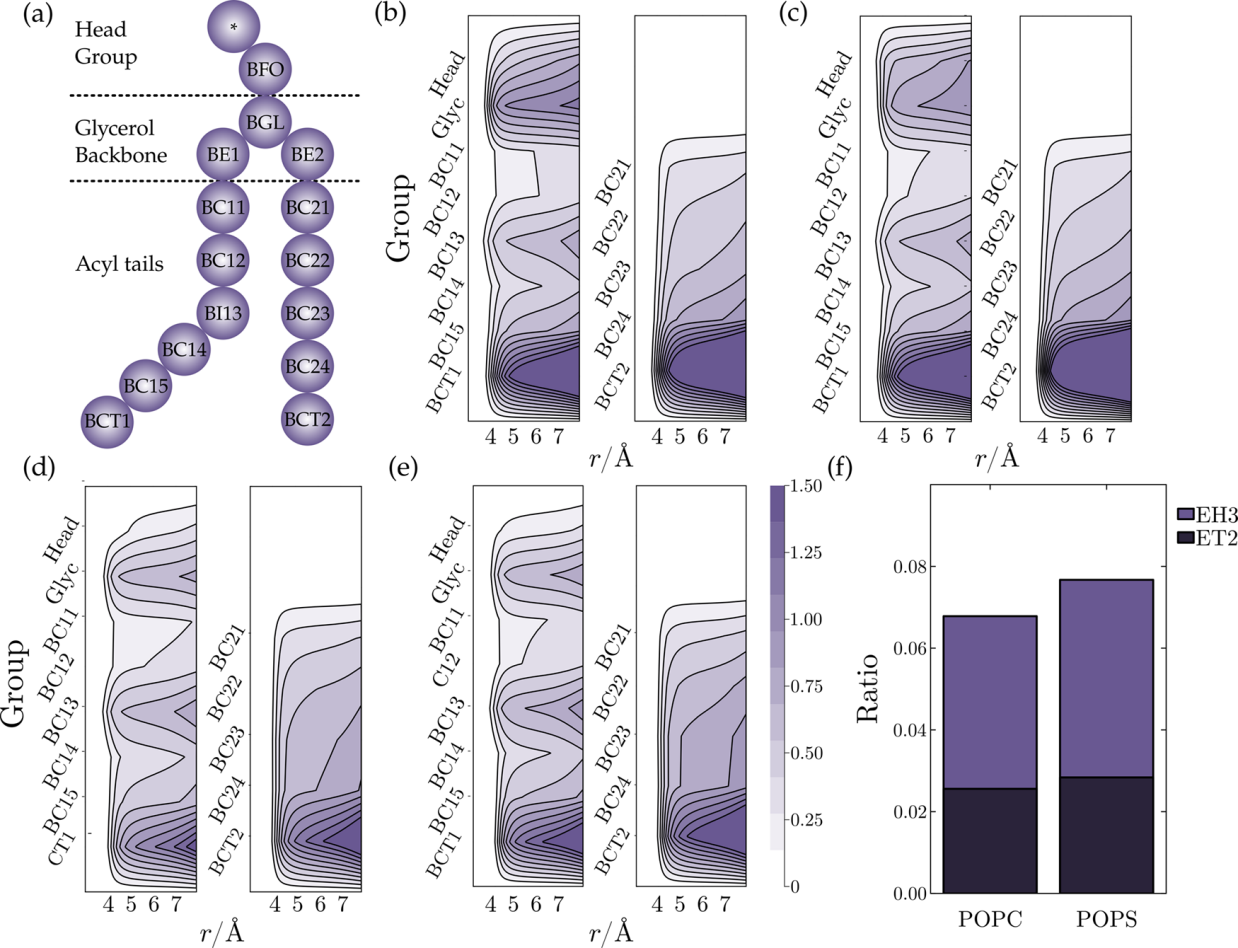
(a) Schematic representation of the beads and the main regions of a generic phospholipid, adapted from Figure 3, where * denotes beads BCO, BPE, or BPSO–BPS. Panels (b) and (c) display 2D density maps illustrating the spatial contributions of lipid beads from POPC and POPS in the EH-3 region. Panels (d) and (e) show the contributions of lipid beads from POPC and POPS in the ET-2 region, respectively. Panel (f) exhibits the head-to-tail interaction ratio for EH-3 and ET-2 in POPC and POPS. Higher density values correspond to greater lipid coordination with residues in these regions. Minimum protein–lipid distances are defined by *r*, calculated as the shortest distance in angstroms between any pair of protein and lipid atoms.

In the highly amphipathic EH-3 region, which possesses the largest hydrophobic moment, interactions are dominated by the lipid headgroups, glycerol moieties, and terminal methyl. For POPS (Figure 10b), this effect appears more pronounced than that in POPC (Figure 10c). The coordination map for POPS indicates a noticeably localized density at the headgroup, with a gradual reduction in contribution from the acyl tails and a strong contribution from the terminal methyl. This pattern suggests a preferential interaction guided by electrostatic forces. The negatively charged phosphoserine headgroup of POPS tends to associate with the polar/charged face of the amphipathic helix, in line with previous reports of POPS showing increased affinity for basic residues.

Conversely, in the hydrophobic transmembrane ET-2 region (Figure 10d,e), the interaction pattern appears different, in which coordination is more broadly distributed. This suggests that the interaction is mainly influenced by hydrophobic interactions between the nonpolar protein surface and the lipid tails. In comparison, the overall coordination of POPS with ET-2 is slightly less pronounced. The polar and charged POPS headgroup is less compatible with the nonpolar transmembrane environment, leading to a lower lipid density. In these cases, the interactions seem more dependent on the acyl tails, while the overall contribution remains smaller.

Such observations are clearly observed in Figure 10f, where the head–tail interaction ratios for EH-3 and ET-2 are displayed. Here, an interaction is defined as a coordination event. For the amphipathic helix EH-3, we observed a clear increase in the head-to-tail ratio when moving from POPC (0.0678) to POPS (0.0767). This result supports the hypothesis that electrostatic forces guide the interaction, enhancing the relative contribution of the anionic POPS headgroup in polar protein environments, which is consistent with the formation of domains enriched in specific lipids.

For ET-2, the head-to-tail ratio for POPS (∼0.0256) was comparable to that of POPC (∼0.0284). This suggests that the interaction is not governed solely by hydrophobic bonding with the helix core. Although the ET-2 helix core is hydrophobic, its terminal regions at the protein–lipid interface contain serine residues that may be forming specific interactions with the anionic headgroup of POPS. This interfacial interaction may influence the balance between polar and nonpolar contacts, leading to a headgroup contribution comparable to that of POPC.

Overall, across all regions examined, the CN was mainly influenced by contributions from the lipid headgroups, the glycerol backbones, and the terminal methyl in the acyl tail, indicating a consistent interaction pattern across both envelope and membrane proteins (Figures S22–S27). Within this overall consistency, however, nuanced differences in lipid preferences emerge. These distinctions, although subtle, are systematically observed for all lipid species and across different envelope regions, suggesting that local topological and electrostatic variations modulate lipid engagement. In particular, the recurrent accumulation of POPS around basic residues such as Lys480 (E protein) and Lys60 (M protein) supports the view that electrostatic forces promote local lipid enrichment, contributing to the formation of microdomains within the otherwise homogeneous membrane composition.^72^

### Selective Lipid Interactions at Protein–Membrane Interfaces—Part II

Helical wheel projections were used to visualize the contribution of each lipid at the residue level, allowing the type of interaction to be associated with the structural nature of the helical segments.

Figure 11 shows the normalized difference of the coordination of POPC versus POPS for EH-3 and ET-2, amphipathic membrane and transmembrane helices, respectively. In Figure 11a, the amphipathic helix EH-3 shows preferential interactions of Phe, Ser, and Lys with POPS, while most other residues display stronger affinity for POPC. The interactions of Ser and Lys with POPS are primarily driven by electrostatic forces, whereas Phe is positioned close to these polar and charged residues on the polar face of the helix, which explains its POPS preference. In contrast, the majority of residues prefer to interact with POPC, reflecting the complexity of residue–lipid interactions. This outcome is particularly noteworthy, as one would expect POPS to preferentially coordinate positively charged residues, yet the observed pattern reveals a more nuanced interplay between residue chemistry, helix orientation, and lipid environment.

**Figure 11:**
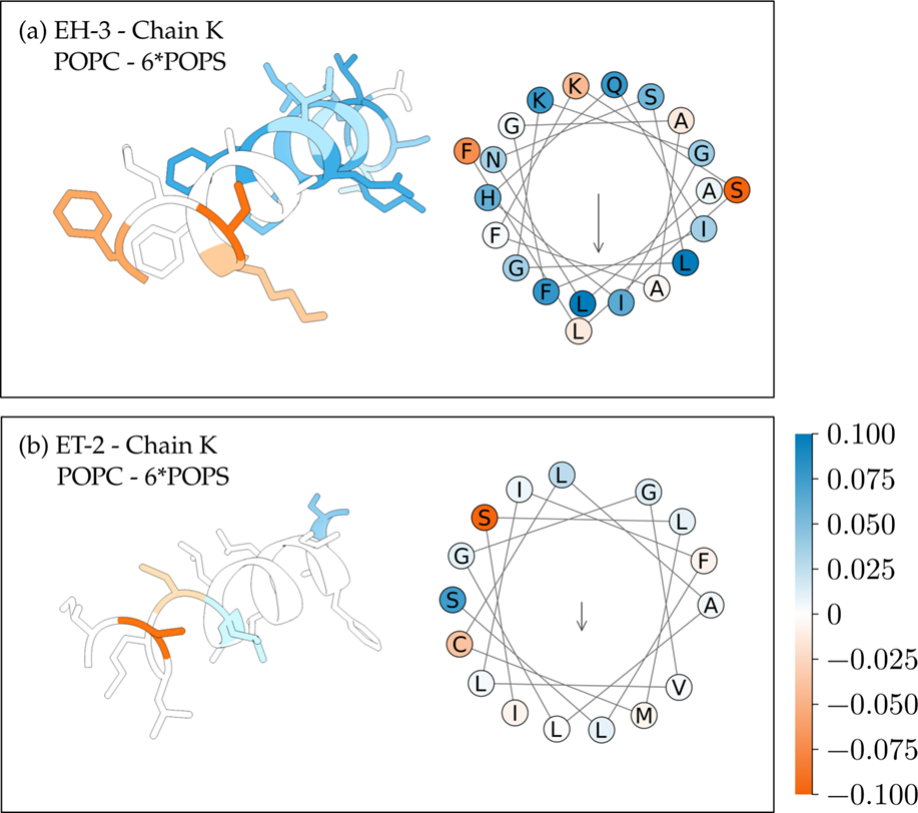
3D representations and helical wheel projections of segments from the E and M proteins of the Zika virus, with a gradient of color representing the scaled difference of coordination of POPC and POPS lipids. (a) EH-3 and (b) ET-2 correspond to α-helices from chain K of the E protein. The arrows represent the hydrophobic moment perpendicular to the helices.

In contrast, the transmembrane ET-2 helix, which contains only two polar residues (Figure 11b), displays minimal differences in lipid preference, with most residues interacting similarly with POPC and POPS. The main exceptions are Ser499, positioned at the helix periphery and likely establishing localized contacts with POPS, and Ser485, situated at the opposite end of the helix, where lipid coordination is more strongly modulated by the surrounding environment. However, because ET-2 is deeply inserted into the membrane and composed predominantly of hydrophobic residues, its interactions are less sensitive to headgroup composition, resulting in lower variability and reduced lipid selectivity overall. The modest differences observed for the two Ser residues are therefore more consistent with local packing effects than with strong electrostatic drivers.

The amphipathic EH helices (EH-1, EH-2, and EH-3) are positioned near the membrane surface and display distinct interaction profiles with the bilayer. EH-1 contains one lysine and several hydrophobic residues that promote stable association at the interface, while EH-2 is notable for its single tryptophan residue, which anchors the helix through interactions with lipid headgroups. EH-3 combines a larger fraction of hydrophobic residues with neutral polar side chains, supporting surface association mediated by both hydrophobic and polar contacts. These amphipathic segments interact with lipid headgroups and glycerol beads, particularly in EH-3, where interactions are spatially organized and consistent with a surface-parallel orientation.

The ET helices adopt transmembrane configurations characterized by strong hydrophobicity and low hydrophobic moments, consistent with full insertion into the bilayer. ET-1 is anchored by tryptophans at the interface and further stabilized by aromatic and hydrophobic residues, while ET-2 similarly interacts through hydrophobic contacts, with serines at the interface contributing complementary headgroup interactions. The MH helices display varied behaviors: MH-1 shows amphipathic character, with aromatic and polar residues favoring interfacial alignment, whereas MH-2 and MH-3 are more hydrophobic, interacting primarily with lipid tails, in line with their transmembrane character. A further analysis of each helical wheel is described in the SI (Figures S28–S36).

Table 2 integrates the physicochemical parameters obtained from the helical wheel analysis with the observed lipid interaction profiles, providing a comprehensive overview of how each segment contributes to membrane association. This synthesis of structural, chemical, and functional descriptors underscores the mechanistic basis by which different viral protein segments interact with specific regions of the lipid membrane. References in the ‘Features’ column identify the structural and functional motifs described; all other quantitative descriptors in Table 2 are original results derived from the molecular dynamics analyses performed in this study or from the structure of the mature virion.

**Table 2:** Structural and Functional Features of α-Helical Regions from Zika Virus E and M Proteins, Including Hydrophobicity (According to the Fauchère and Pliska Scale^70^), Hydrophobic Moment, Residue Polarity, Lipid Interaction Patterns, Orientation within the Membrane, and Associated Features

**subunit**	**<H>**	**hydrophobic moment**	**key residues**	**lipid interactions**	**features**	**references**
**EH-1**	0.320	0.381	Thr406, Ile407, Phe411, Thr414	interacts with POPC and POPE headgroups + glycerol. Occasional POPS enrichment near Lys residue	key components of E protein trimeric rearrangement during fusion	^73^
**EH-2**	0.410	0.360	Trp429	minimal lipid interaction; prefers headgroups via Trp429 indole hydrogen bonding		
**EH-3**	0.583	0.511	Leu438, Asn439, Ser440, Leu441, Ile445, His446, Gln447, Ile448, Phe449, Phe453, Ser455, Leu456, Phe457	strong lipid coordination with both headgroup and tail beads. Aromatic residues increase hydrophobic interactions		
**ET-1**	1.119	0.136	Trp462, Phe463, Thr470, Met473, Trp474	strong hydrophobic interactions with lipid tails. Trp462 and Trp474 act like orientational anchors	antiparallel helices; cross the two leaflets of the lipid bilayer, and mediate the fusion between the virus and the membrane of the target cell	^16,23,24^
**ET-2**	1.132	0.217	Ser485, Cys488, Phe497, Ser499	strong tail contacts with membrane; stabilization via serine and cysteine with headgroups		
**MH-1**	0.298	0.419	Tyr25, His28, Trp35, Phe37	interacts with lipid headgroups and glycerol portions	involved in E protein conformational change, folding traffic and function of the fusion protein E	^43,73^
**MH-2**	0.715	0.118	Pro40, Gly41, Phe42, Leu44, Ala45, Ile49	deep transmembrane insertion; dominated by lipid tail contacts. Aromatic residues may stabilize bilayer insertion	antiparallel helices; cross the two leaflets of the lipid bilayer, and are involved in E protein conformational change, folding traffic and function of the fusion protein E	^23,24,43^
**MH-3**	0.948	0.136	Thr57, Ser58, Gln59, Lys60, Val61, Tyr63, Val65	similar to MH-2, but shows localized POPS preference at Lys60 and Tyr63, indicating selective electrostatic contribution		

## Conclusions

The present study provides a detailed characterization of lipid–protein interactions within the Zika virus complex proteins, revealing both global and site-specific determinants of membrane association. Clear preferences for specific residues and protein domains emerged among POPC, POPE, and POPS, highlighting the balance among lipid abundance, headgroup chemistry, and electrostatics in shaping the interaction landscape. POPS emerged as a key determinant of local specificity, consistently clustering near conserved lysine residues (e.g., Lys480 in the E protein and Lys60 in the M protein), where electrostatic attraction creates localized microdomains of negative charge enrichment. In contrast, POPC and POPE predominantly mediated hydrophobic and hydrogen-bond interactions, engaging amphipathic and transmembrane helices in ways that reflect residue polarity and spatial orientation.

Distinct lipid preferences were observed across structural motifs. Amphipathic helices, such as EH-1 and EH-3 in the envelope protein and MH-1 in the membrane protein, are aligned parallel to the bilayer surface and engage lipid headgroups and glycerol regions, consistent with their role in membrane association without full insertion. Transmembrane helices (ET-1, ET-2, MH-2, and MH-3) displayed strong coordination with acyl chains, stabilized by aromatic belt residues that anchor interfacial regions while promoting deep bilayer penetration. Moreover, small but consistent variations in lipid coordination across symmetry-related subunits underscore the quasi-equivalence principle, whereby identical protein motifs experience distinct lipid surroundings depending on their geometric placement within the viral shell.

Altogether, these findings demonstrate that the lipid–protein interface of the Zika virus is not solely dictated by lipid abundance but reflects the interplay among helix topology, amphipathicity, and residue chemistry. This refined molecular picture highlights potential lipid-specific contributions to viral stability, membrane fusion, and envelope remodeling, offering mechanistic insights that may extend to other flaviviruses.

## Supplementary Material



## Data Availability

Example scripts used for the analyses described in this study are provided at a GitHub repository (https://tcvmilvv.github.io/TavaresSonoraPantanoMartinez2025.jl), where all scripts are organized, documented, and maintained in a form that is more accessible and reusable for the simulations community. These scripts include routines for computing coordination numbers and visualizing solvation patterns using ComplexMixtures.jl. Users can adapt these examples to analyze similar systems or extend them for custom applications. Additional data can be obtained upon request to authors.

## Notes

Structural overview of the viral envelope and lipid coordination; spatial distribution of lipids and local preferences; selective lipid interactions at protein–membrane interfaces—Part I; helical wheel representations of lipid–helix interactions across envelope–membrane subunits and additional discussion; and example scripts for reproducibility, including workflow setup, coordination number, and residue contribution analyses (PDF)
