# Supplementary material for: Lipid Architecture in the Zika Virus
Source: ACS Phys Chem Au. 2026 Feb 27;6(4):665–77. doi: 10.1021/acsphyschemau.5c00113 (PMC13397441; doi:10.1021/acsphyschemau.5c00113)
Supplement: Supplementary file 1 [file pg5c00113_si_001.pdf]

Supplementary Information of

Lipid Architecture in the Zika Virus

Camila Assis Tavares[1], Martín Soñora[2], Sergio Pantano[2], Leandro Martínez[1,*]

[1]Institute of Chemistry and Center for Computational Engineering & Science, Universidade Estadual de Campinas (UNICAMP), Campinas, SP, Brazil

[2]Biomolecular Simulations Group, Institut Pasteur de Montevideo, Mataojo 2020, Montevideo, Uruguay

Corresponding Author:

*Leandro Martínez: lmartine@unicamp.br, Institute of Chemistry, Universidade de Campinas. 13083-970, Campinas, SP. Brazil. http://m3g.iqm.unicamp.br

Table of Contents

Structural Overview of the Viral Envelope and Lipid Coordination.. 5

Figure S1. Protein–lipid contacts of the E–M complex with (a) POPE in the outer leaflet of chains, (b) POPS in the outer leaflet, (c) POPE in the inner leaflet, and (d) POPS in the inner leaflet. Darker areas indicate regions of more frequent lipid interactions in chains of type K and L...5

Figure S2. Structural mapping of lipid coordination differences across the E–M complex, highlighting the spatial distribution of preferred contacts for POPE, as Hammer-Aitoff projections...6

Figure S3. Hammer–Aitoff projections comparing POPS interaction frequencies across envelope–membrane protein chains...7

Spatial Distribution of Lipids and Local Preferences..8

Figure S4. Weighted coordination number differences between POPC and POPS, projected as 2D density maps for chain K...8

Figure S5. Density plots highlighting how chain K distinguishes coordination preferences between POPE and POPS...9

Figure S6. 2D density visualization of lipid coordination, showing the relative enrichment of POPC versus POPE around chain M.. 10

Figure S7. Coordination landscapes from 2D density maps comparing POPC and POPS interactions with chain M..11

Figure S8. Weighted differences in coordination numbers between POPE and POPS, mapped in two dimensions for chain M.. 12

Figure S9. Comparative density maps illustrating coordination disparities between POPC and POPE around chain O.. 13

Figure S10. Two-dimensional maps of weighted coordination differences for POPC versus POPS along chain O...14

Figure S11. Relative coordination densities of POPE and POPS, resolved on a 2D map for chain O... 15

Figure S12. 2D coordination number density differences between POPC and POPE in the context of chain L...16

Figure S13. Weighted 2D distributions showing how POPC and POPS differ in their coordination with chain L.. 16

Figure S14. Two-dimensional mapping of coordination asymmetries between POPE and POPS for chain L...17

Figure S15. Density-based view of coordination number differences between POPC and POPE, evaluated for chain N... 17

Figure S16. 2D projection of coordination differences contrasting POPC and POPS around chain N... 18

Figure S17. Weighted density maps of coordination number differences for POPE and POPS interactions with chain N.. 18

Figure S18. Visualization of coordination differences between POPC and POPE, displayed as 2D density maps for chain P.. 19

Figure S19. Two-dimensional density mapping of the relative coordination numbers of POPC versus POPS at chain P........ 19
Figure S20. Weighted 2D density plots revealing coordination differences between POPE and POPS with chain P........ 20
Figure S21. Normalized lipid contributions around lysine residues: (a–c) Lys480 of chain M with POPC, POPE, and POPS, respectively; (d–f) Lys480 of chain O with POPC, POPE, and POPS; (g–i) Lys60 of chain N with POPC, POPE, and POPS; and (j–l) Lys60 of chain P with POPC, POPE, and POPS........ 21
Selective Lipid Interactions at Protein–Membrane Interfaces - Part I........ 22
Figure S22. 2D density maps of lipid bead contributions from POPC, POPE, and POPS across the EH, ET, and MH subunits of chain K........ 22
Figure S23. Comparative 2D density maps showing how POPC, POPE, and POPS beads distribute around chain M subunits EH-1–3, ET-1–2, and MH-1–3........ 23
Figure S24. 2D projections of lipid bead density reveal the spatial organization of POPC, POPE, and POPS interactions with EH, ET, and MH subunits in the chains of type O........ 24
Figure S25. Spatial density maps (2D) illustrating the specific contributions of POPC, POPE, and POPS beads to the lipid environment of EH, ET, and MH subunits in the chain L........ 25
Figure S26. 2D density plots depicting the localization of lipid beads from POPC, POPE, and POPS in relation to chains N EH-1–3, ET-1–2, and MH-1–3 subunits........ 26
Figure S27. Mapping of lipid bead contributions (POPC, POPE, and POPS) as 2D densities highlights distinct interaction patterns across EH, ET, and MH subunits in chain P........ 27
Selective Lipid Interactions at Protein–Membrane Interfaces - Part II........ 28
Figure S28. Helical wheel representations of the subunits of chains of type K and L, illustrating POPC contribution at 5 Å, The arrows indicate the hydrophobic moment........ 32
Figure S29. Helical wheel diagrams of type K and L subunits, highlighting POPE contacts per residue within 5 Å........ 33
Figure S30. Helical wheel plots of chains K and L, where POPS contributions at 5 Å are shown........ 34
Figure S31. Helical wheel representations of chains M and N, showing that POPC interactions at 5 Å around residues aligned with the hydrophobic moment........ 35
Figure S32. POPE contacts mapped onto helical wheels of type M and N subunits at 5 Å, revealing the orientation of the hydrophobic moment........ 36
Figure S33. Helical wheel of chains M and N with POPS contacts at 5 Å........ 37
Figure S34. Helical wheels of chains O and P depicting POPC contributions within 5 Å of the subunits........ 38
Figure S35. Helical wheel diagrams of subunits O and P. POPE contributions are mapped at 5 Å........ 39
Figure S36. Helical wheels of chains O and P depicting POPS contributions within 5 Å, with arrows indicating hydrophobic moment vectors relevant for membrane embedding. 40

Additional Information **41**

Figure S37. Time series of the relative fractions of POPC, POPE, and POPS in the inner (left) and outer (right) leaflets over the 2 μs coarse-grained simulation. Lipids were classified based on the position of a representative headgroup bead relative to the virion center. The compositions remain essentially constant at the imposed 6:3:1 ratio, with only small fluctuations around the mean, indicating that transbilayer lipid exchange does not lead to net compositional changes on the simulated timescale 41

Figure S38. Coordination number as a function of time per residue W429, shown separately for contacts with POPC (a), POPE (b), and POPS (c). This residue is presented as a representative example to illustrate the equilibration of lipid–protein contacts. The absence of systematic drifts and the presence of stationary fluctuations indicate that the system is well equilibrated over the analyzed time window 43

Figure S39. Fluctuations of the coordination number of inner POPC along the simulation and block analysis for time-correlated data, showing the convergence of the worst block value to the global mean, the dependence of the standard error on the block size, the autocorrelation function with integrated correlation time, and the effective number of independent samples, calculated with the block-averages method of MolSimToolkit.jl.. 43

Figure S40. Fluctuations of the coordination number of outer POPC along the simulation and block analysis for time-correlated data, showing the convergence of the worst block value to the global mean, the dependence of the standard error on the block size, the autocorrelation function with integrated correlation time, and the effective number of independent samples, calculated with the block-averages method of MolSimToolkit.jl.. 44

Figure S41. Fluctuations of the coordination number of inner POPE along the simulation and block analysis for time-correlated data, showing the convergence of the worst block value to the global mean, the dependence of the standard error on the block size, the autocorrelation function with integrated correlation time, and the effective number of independent samples, calculated with the block-averages method of MolSimToolkit.jl.. 45

Figure S42. Fluctuations of the coordination number of outer POPE along the simulation and block analysis for time-correlated data, showing the convergence of the worst block value to the global mean, the dependence of the standard error on the block size, the autocorrelation function with integrated correlation time, and the effective number of independent samples, calculated with the block-averages method of MolSimToolkit.jl.. 46

Figure S43. Fluctuations of the coordination number of inner POPS along the simulation and block analysis for time-correlated data, showing the convergence of the worst block value to the global mean, the dependence of the standard error on the block size, the autocorrelation function with integrated correlation time, and the effective number of independent samples, calculated with the block-averages method of MolSimToolkit.jl.. 47

Figure S44. Fluctuations of the coordination number of outer POPS along the simulation and block analysis for time-correlated data, showing the convergence of the worst block value to the global mean, the dependence of the standard error on the block size, the autocorrelation function with integrated correlation time, and the effective number of independent samples, calculated with the block-averages method of MolSimToolkit.jl.. 48

Additional References **49**

Structural Overview of the Viral Envelope and Lipid Coordination

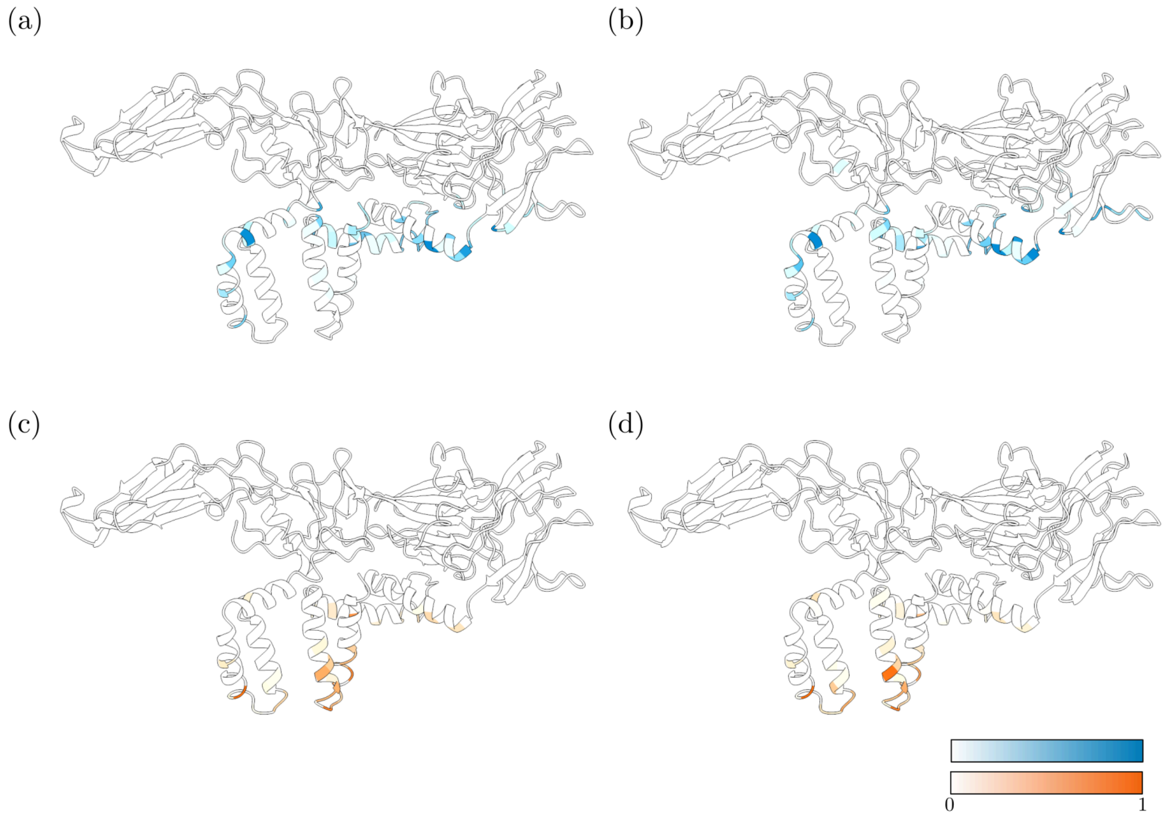

Figure S1. Protein–lipid contacts of the E–M complex with (a) POPE in the outer leaflet of chains, (b) POPS in the outer leaflet, (c) POPE in the inner leaflet, and (d) POPS in the inner leaflet. Darker areas indicate regions of more frequent lipid interactions in chains of type K and L.

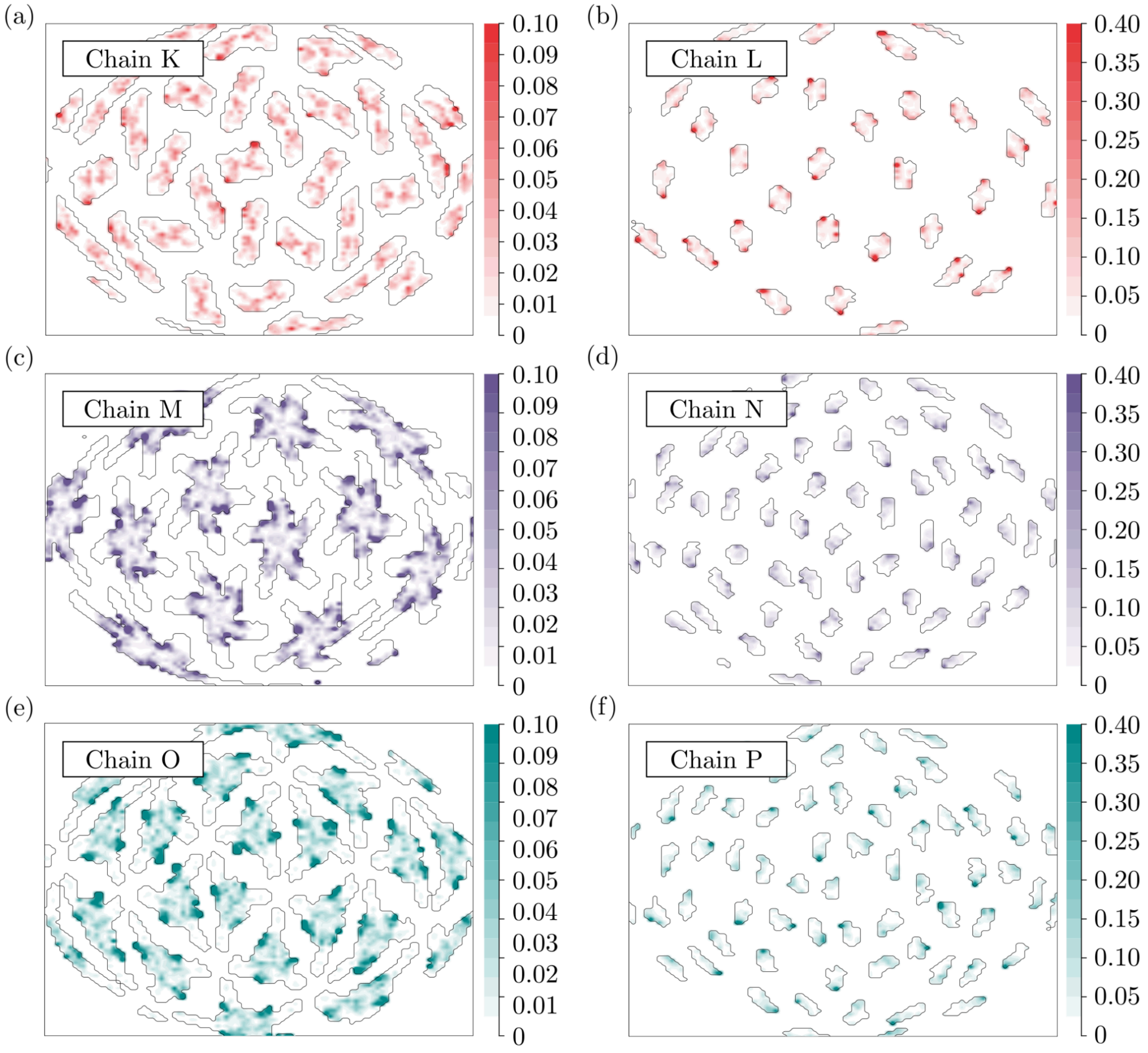

Figure S2. Structural mapping of lipid coordination differences across the E–M complex, highlighting the spatial distribution of preferred contacts for POPE, as Hammer-Aitoff projections.

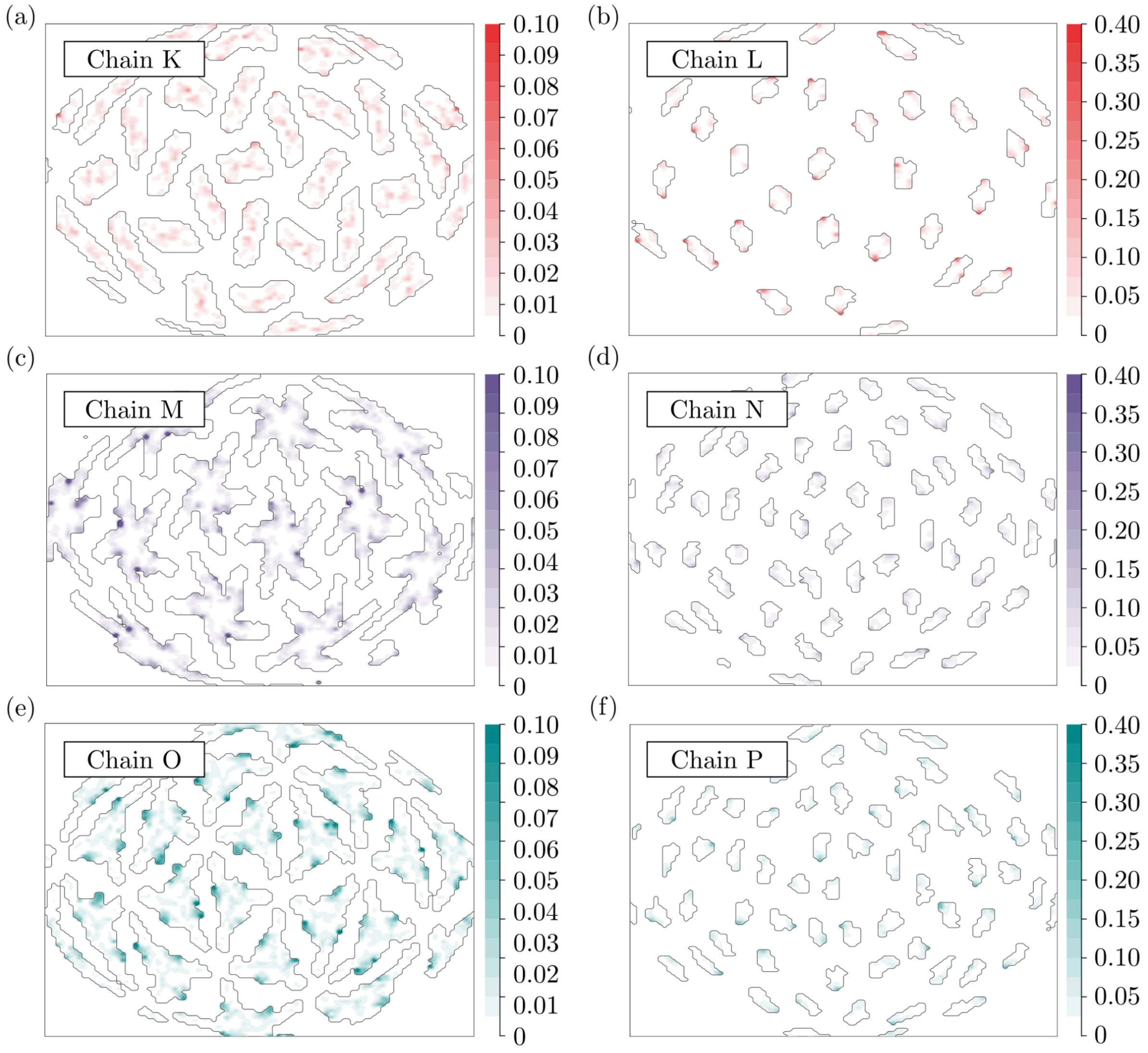

Figure S3. Hammer–Aitoff projections comparing POPS interaction frequencies across envelope–membrane protein chains.

Spatial Distribution of Lipids and Local Preferences

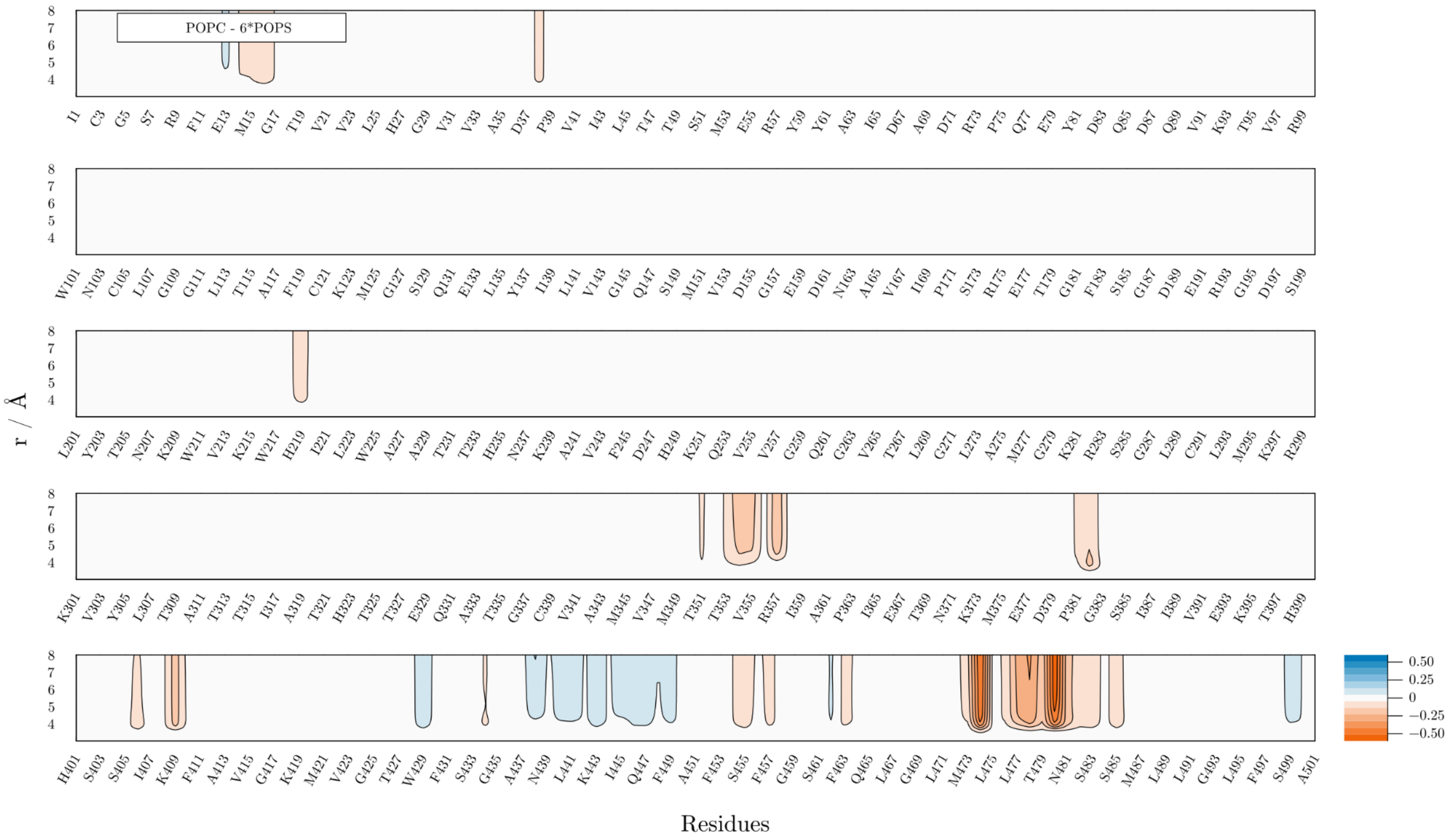

Figure S4. Weighted coordination number differences between POPC and POPS, projected as 2D density maps for chain K.

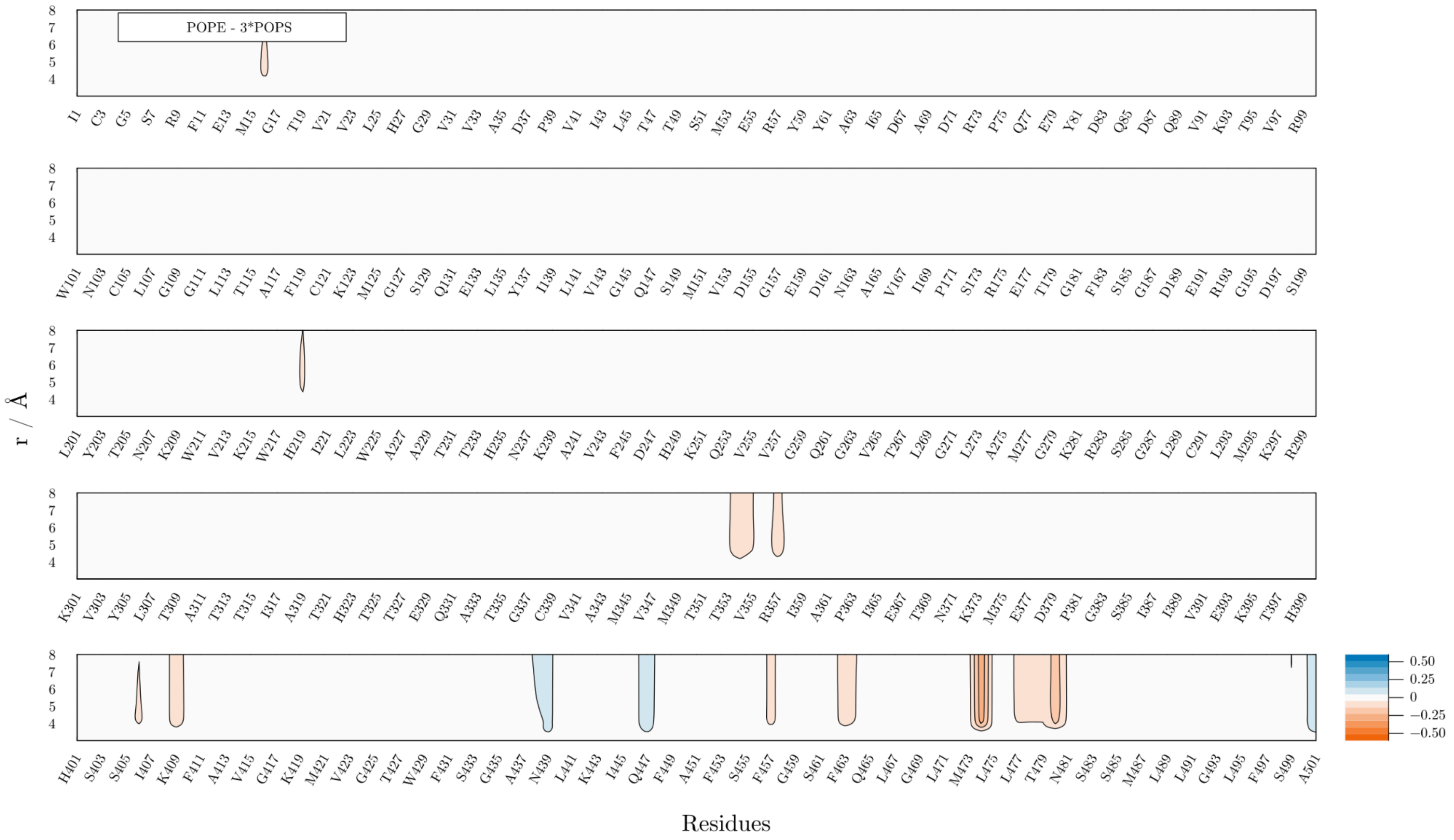

Figure S5. Density plots highlighting how chain K distinguishes coordination preferences between POPE and POPS.

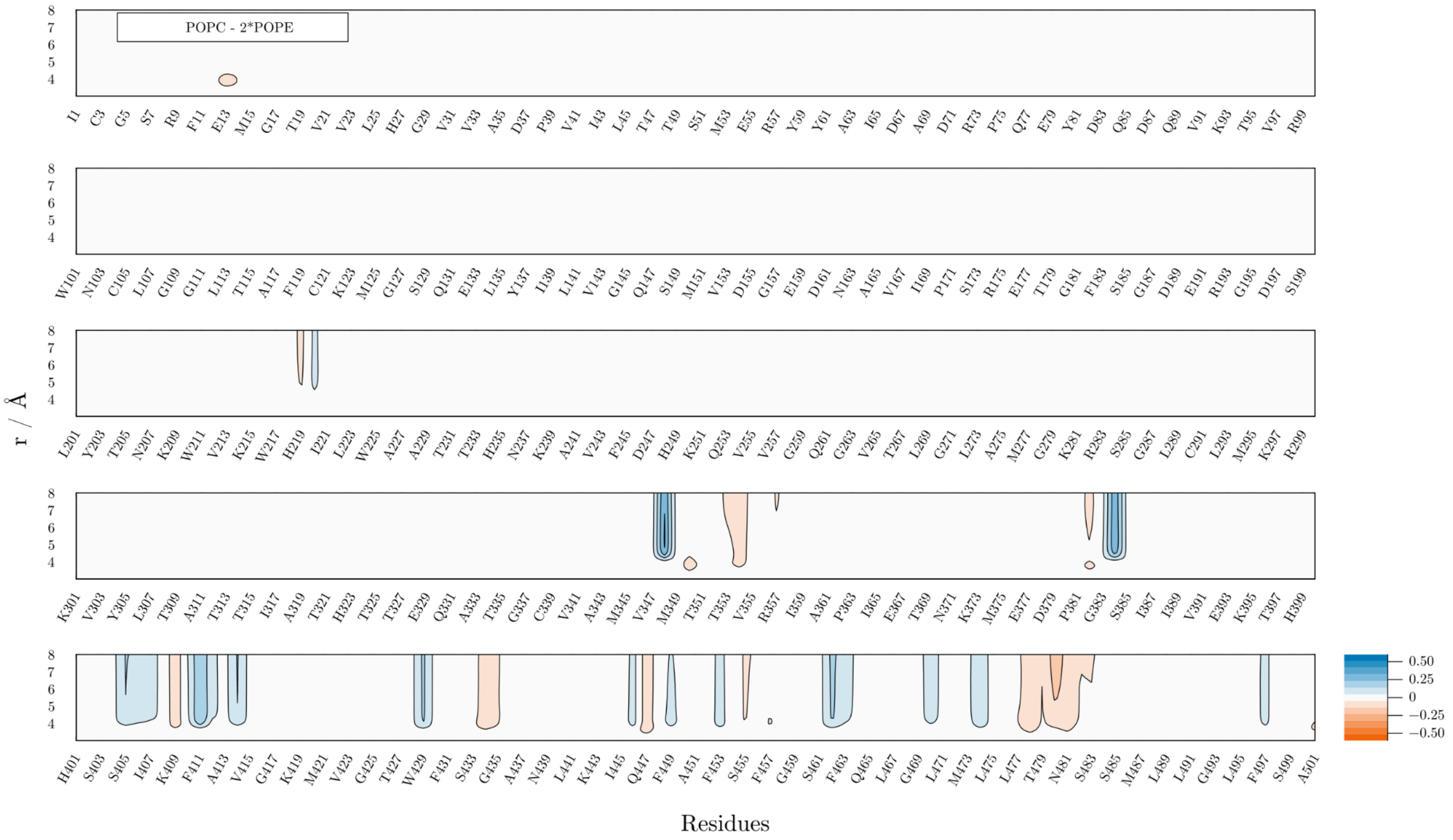

Figure S6. 2D density visualization of lipid coordination, showing the relative enrichment of POPC versus POPE around chain M.

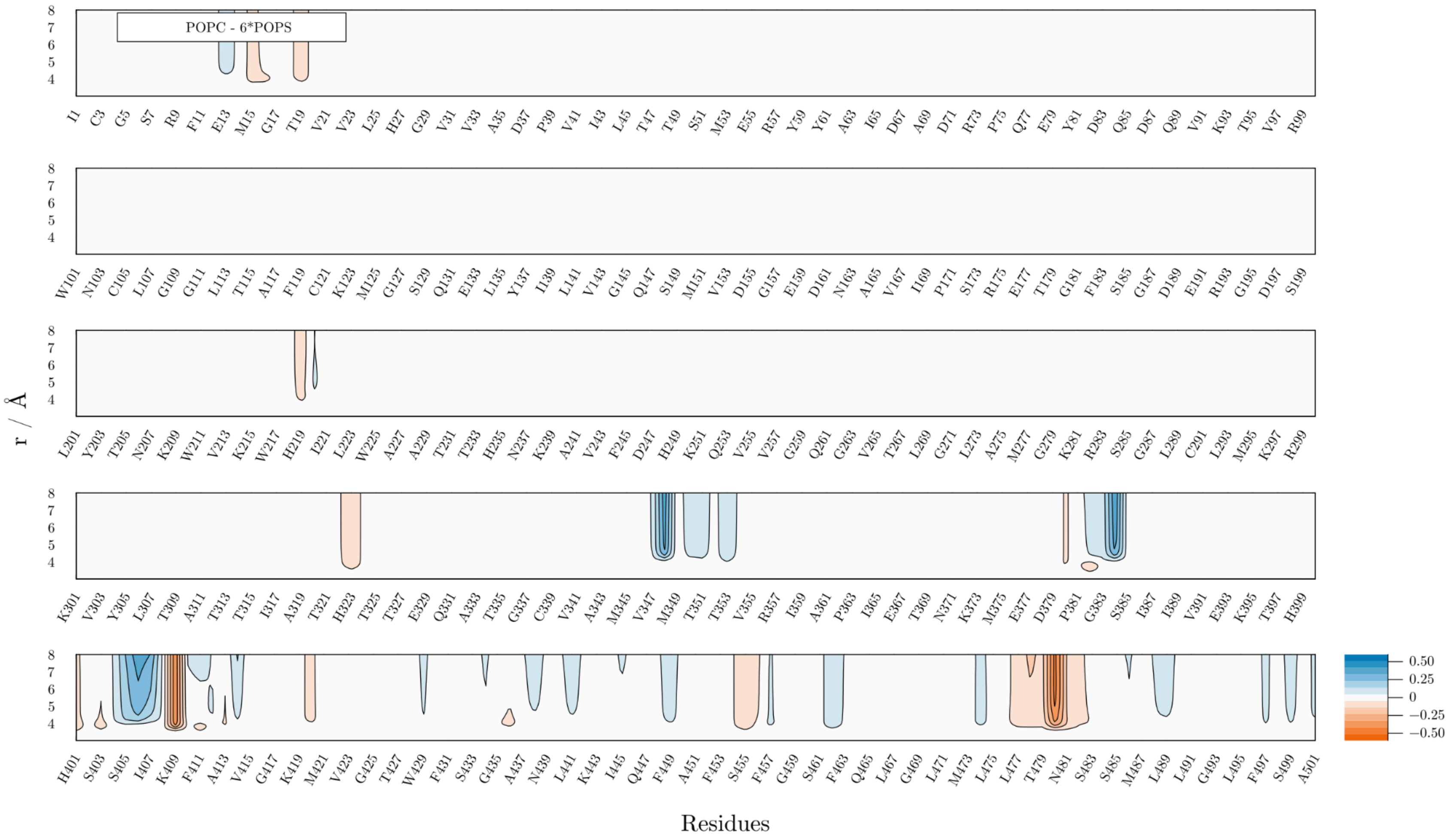

Figure S7. Coordination landscapes from 2D density maps comparing POPC and POPS interactions with chain M.

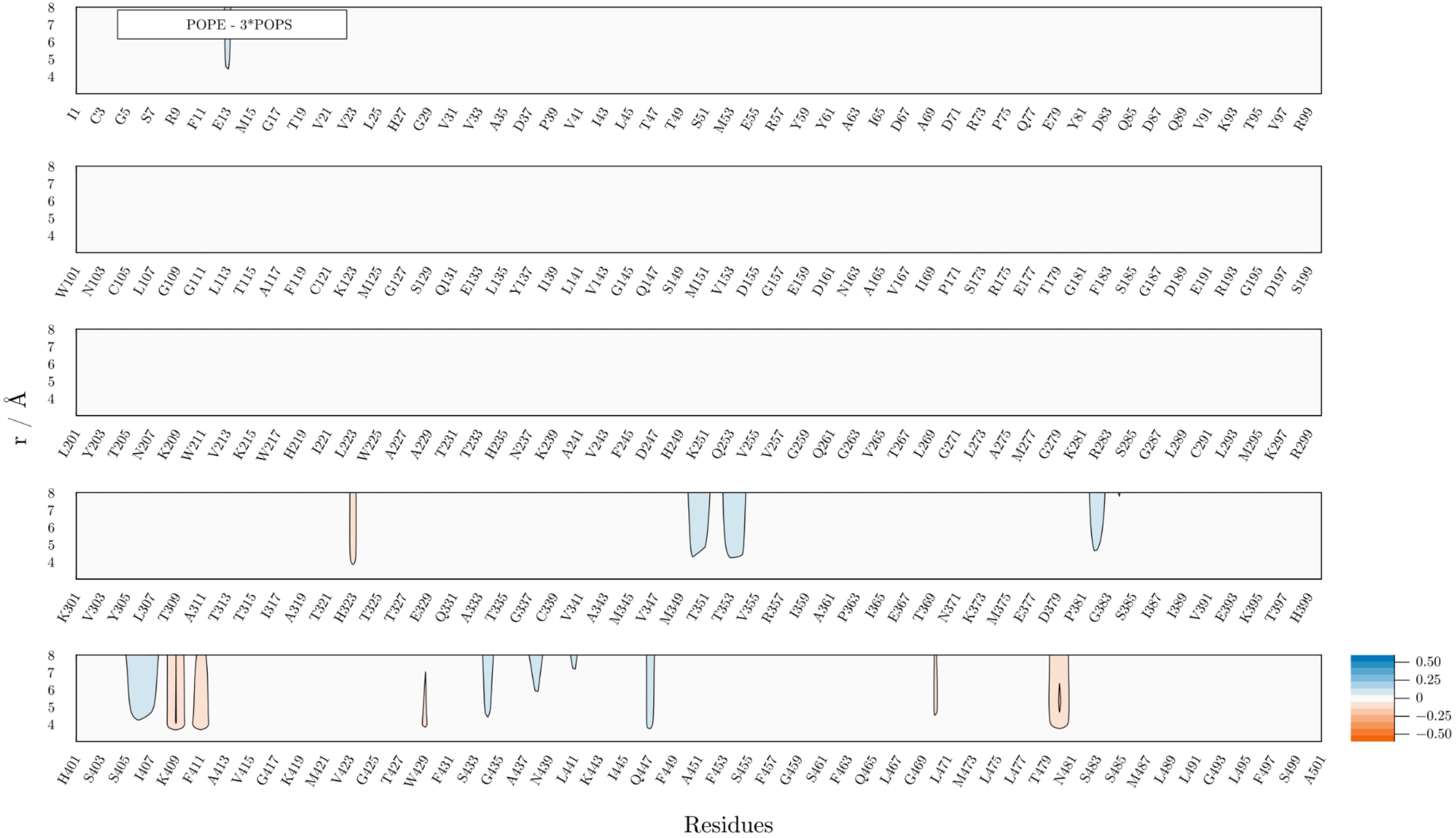

Figure S8. Weighted differences in coordination numbers between POPE and POPS, mapped in two dimensions for chain M.

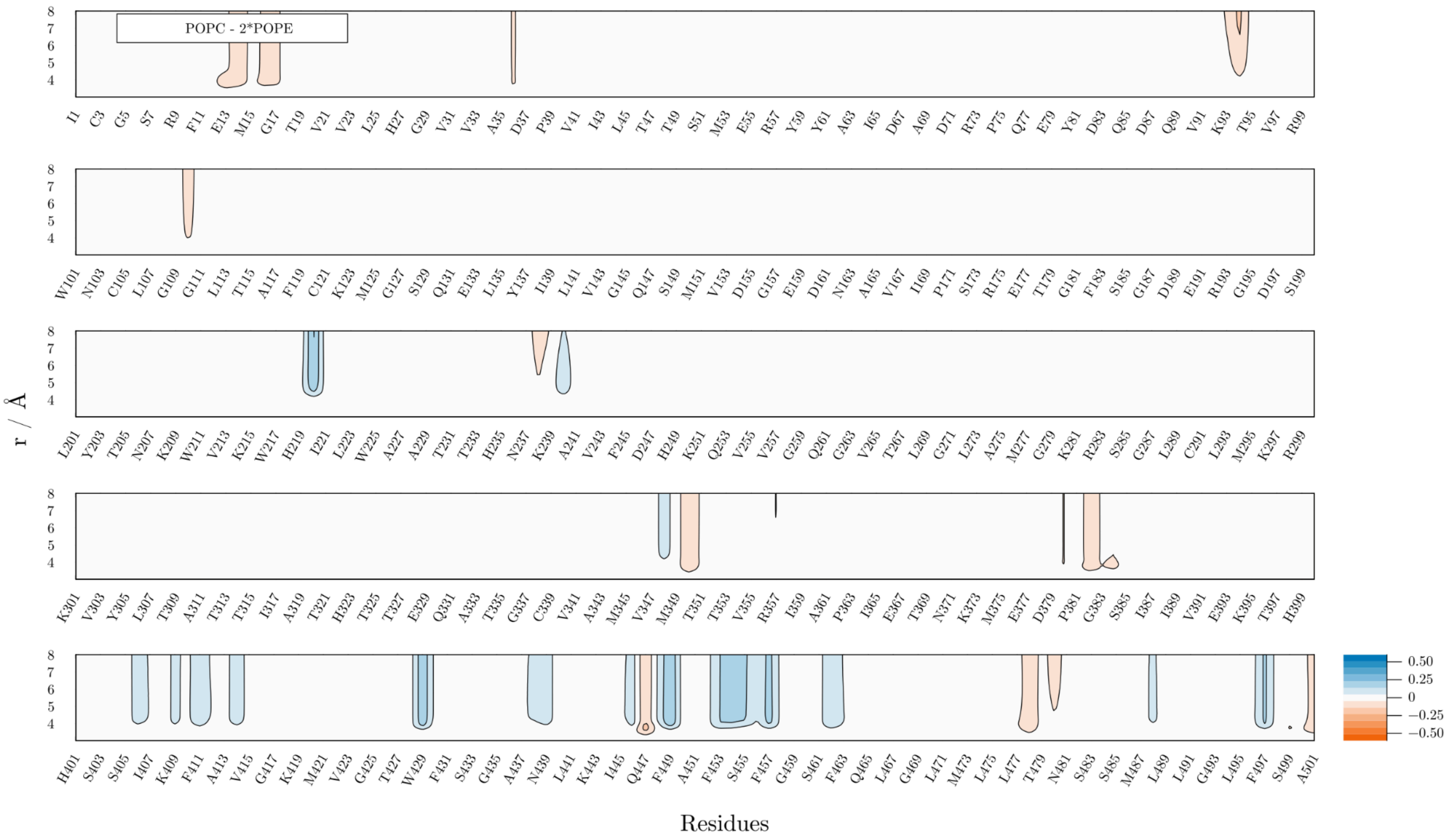

Figure S9. Comparative density maps illustrating coordination disparities between POPC and POPE around chain O.

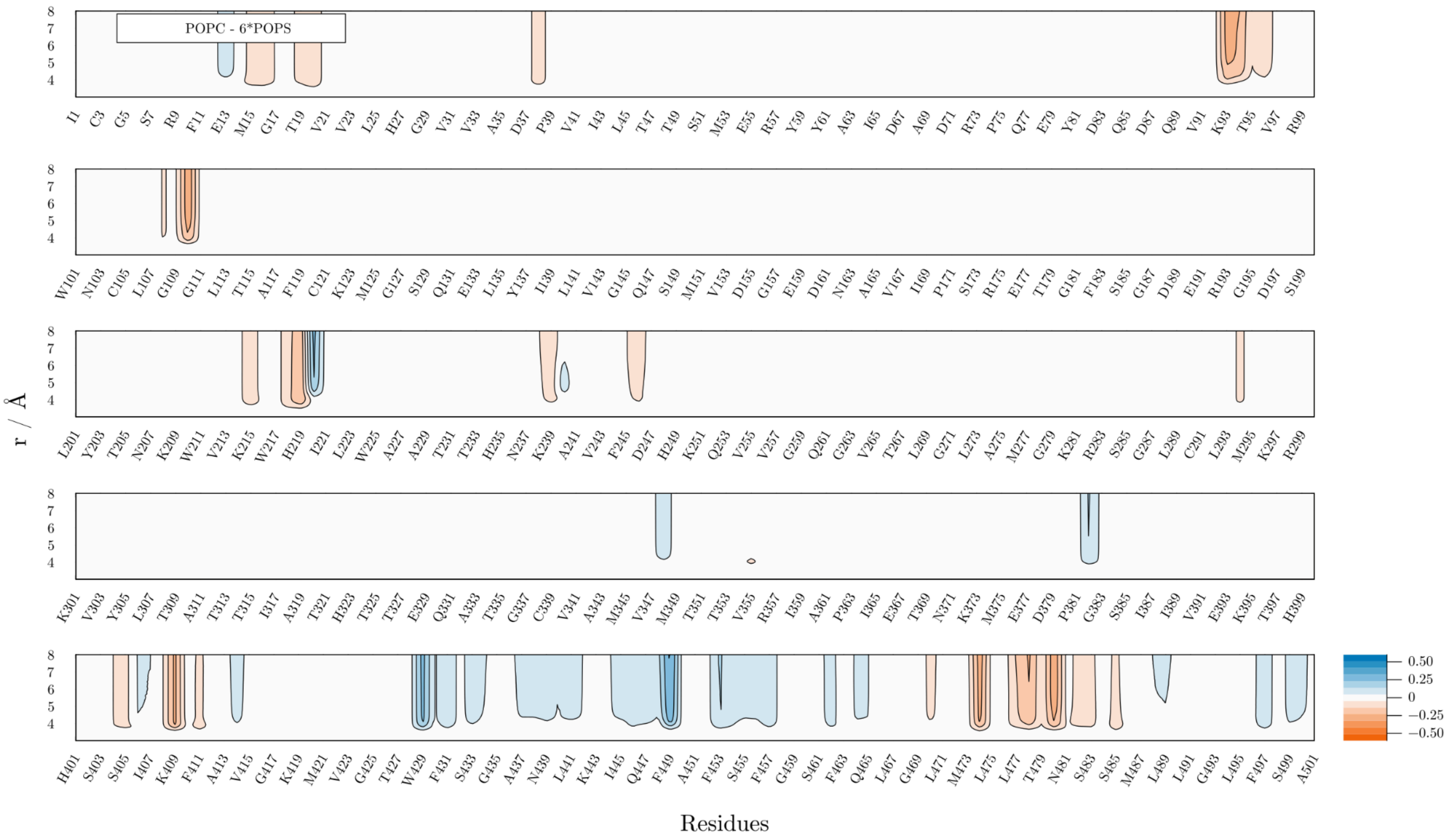

Figure S10. Two-dimensional maps of weighted coordination differences for POPC versus POPS along chain O.

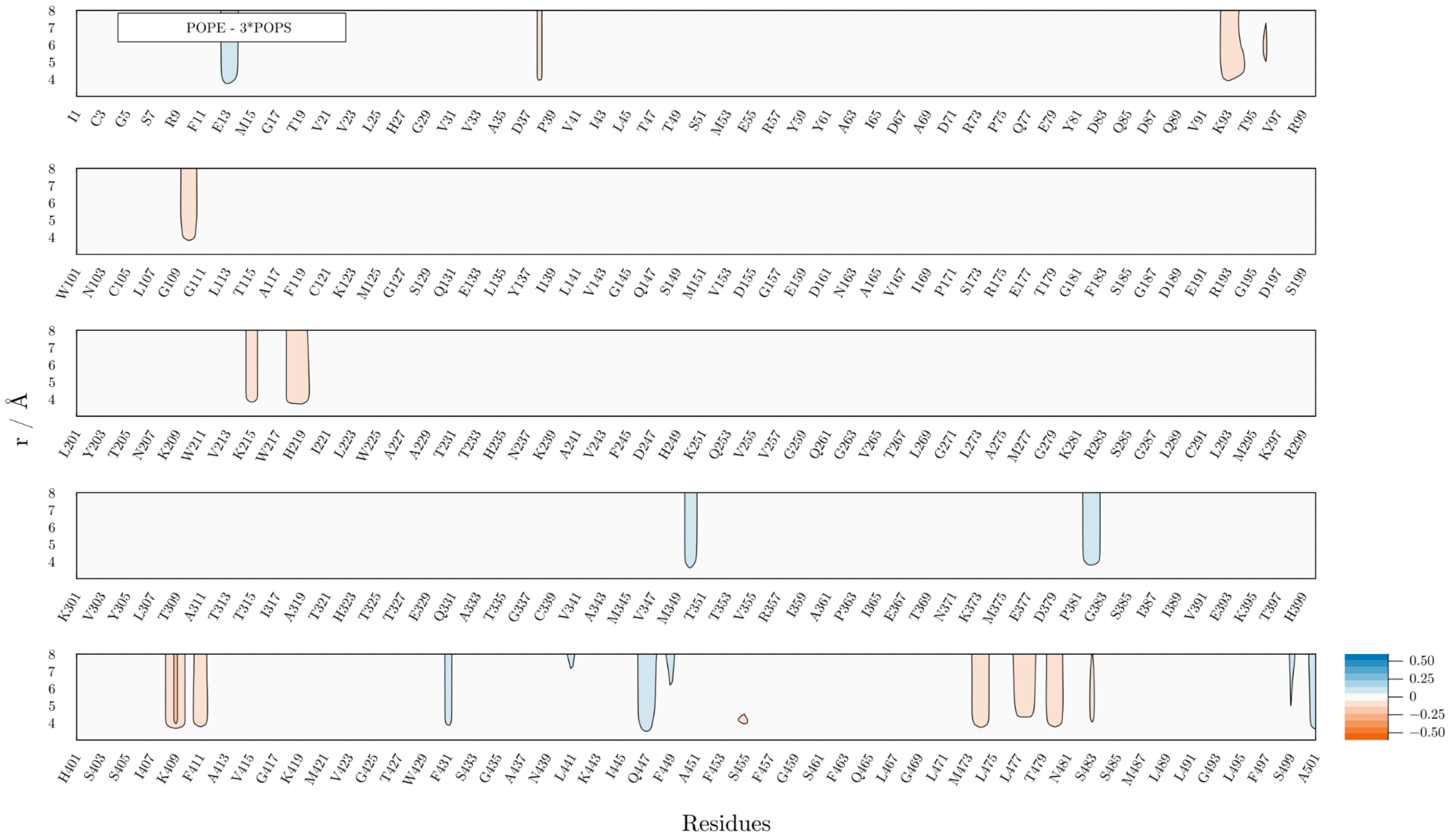

Figure S11. Relative coordination densities of POPE and POPS, resolved on a 2D map for chain O.

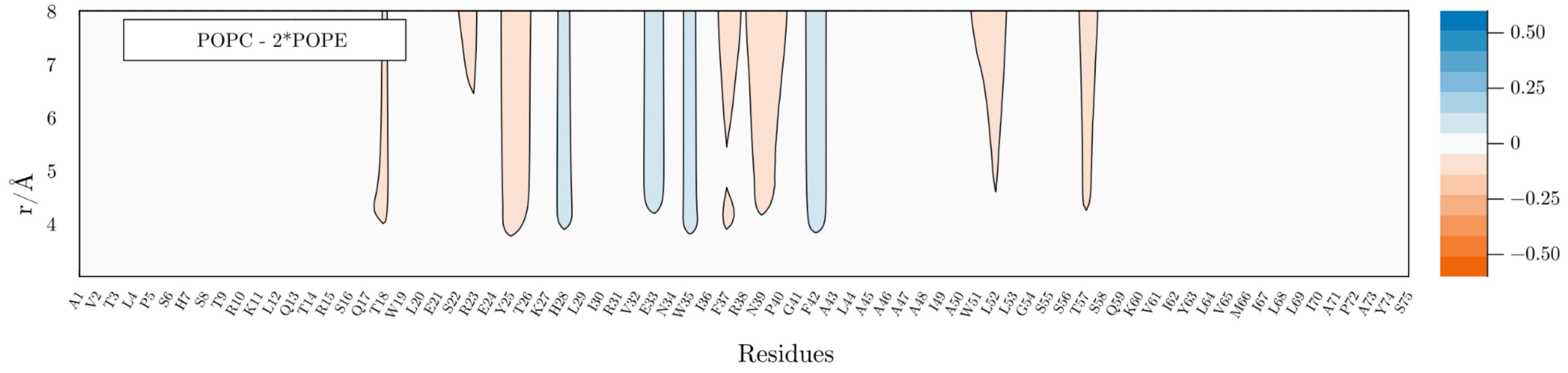

Figure S12. 2D coordination number density differences between POPC and POPE in the context of chain L.

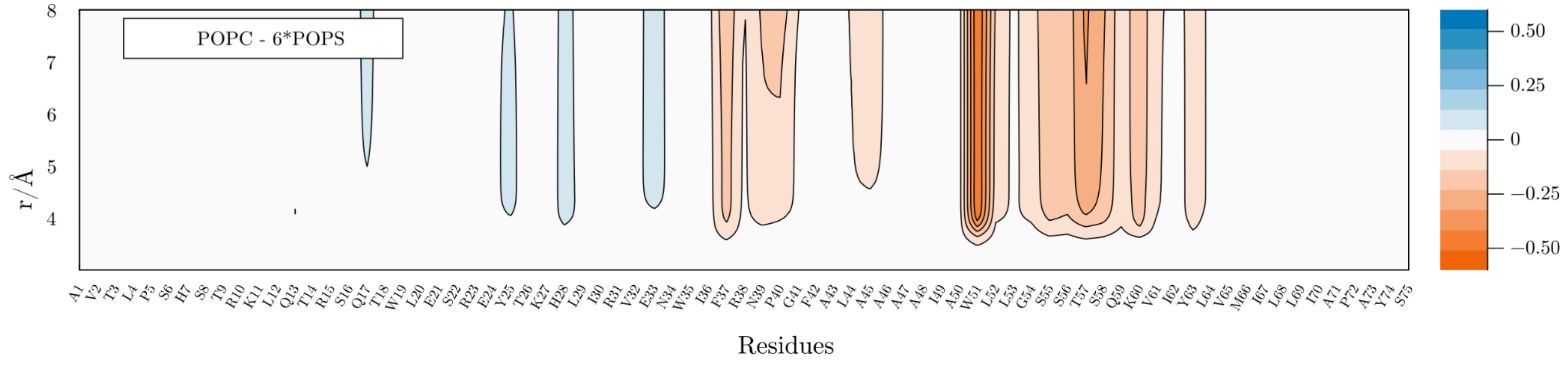

Figure S13. Weighted 2D distributions showing how POPC and POPS differ in their coordination with chain L.

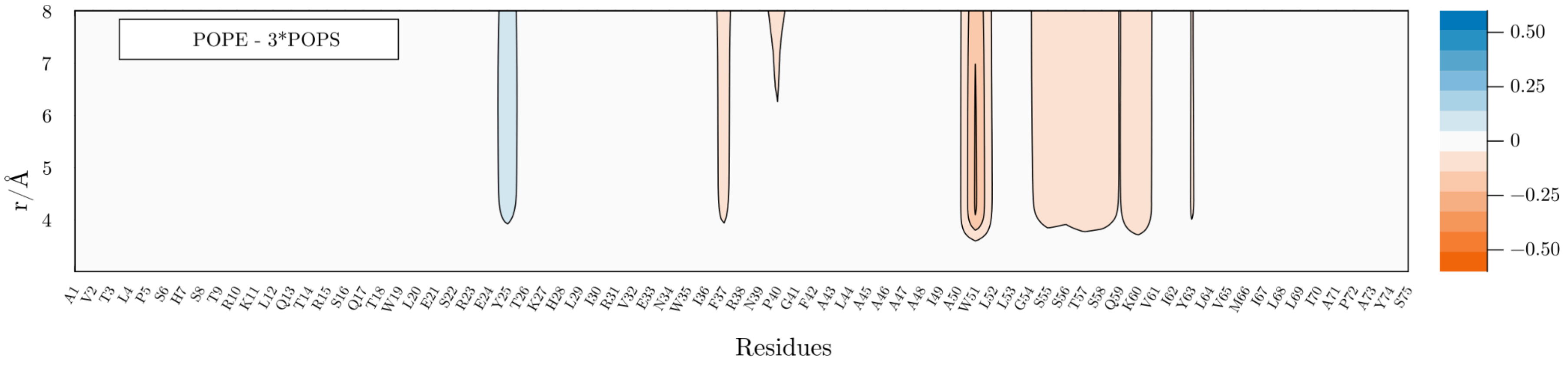

Figure S14. Two-dimensional mapping of coordination asymmetries between POPE and POPS for chain L.

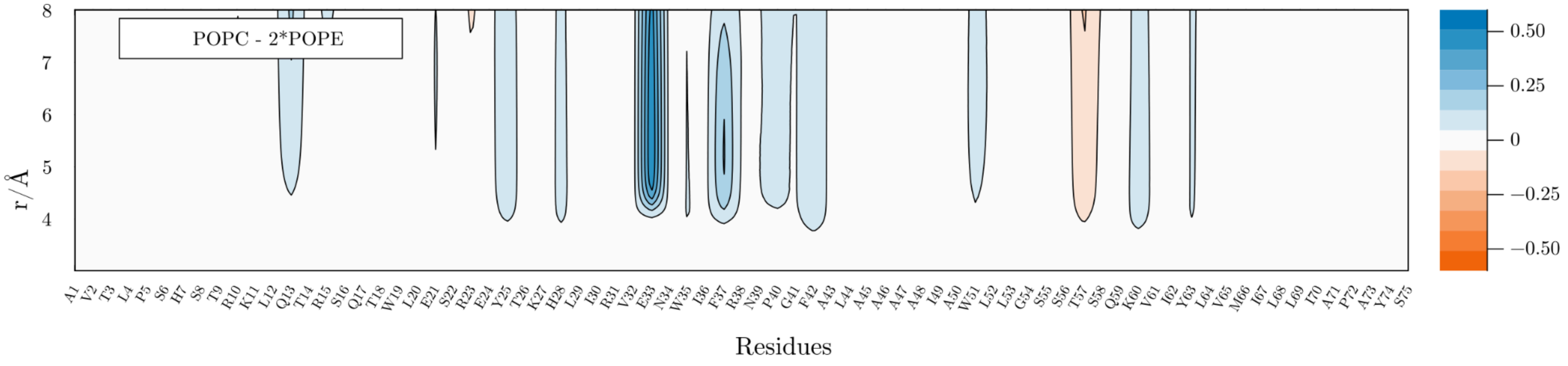

Figure S15. Density-based view of coordination number differences between POPC and POPE, evaluated for chain N.

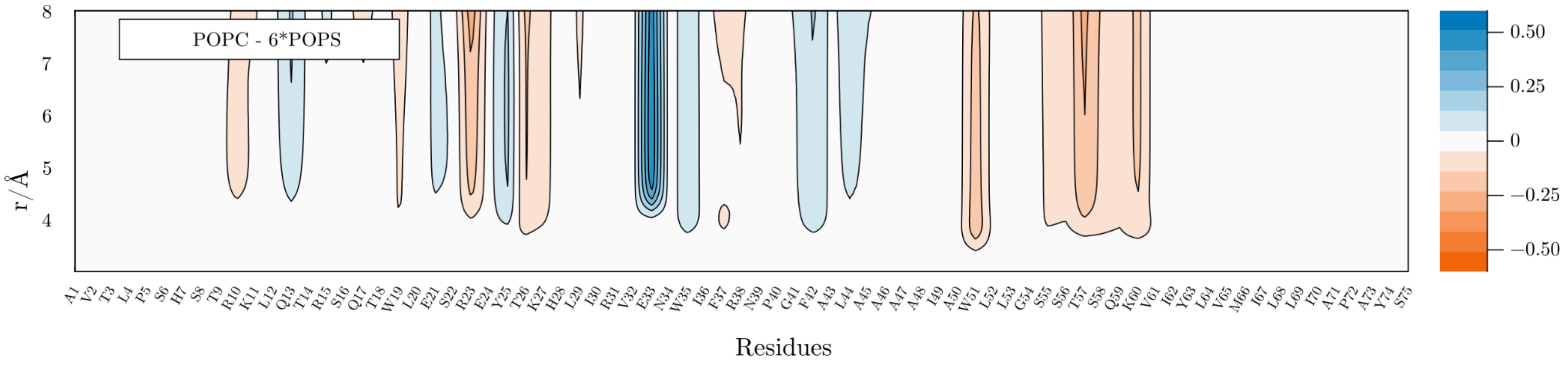

Figure S16. 2D projection of coordination differences contrasting POPC and POPS around chain N.

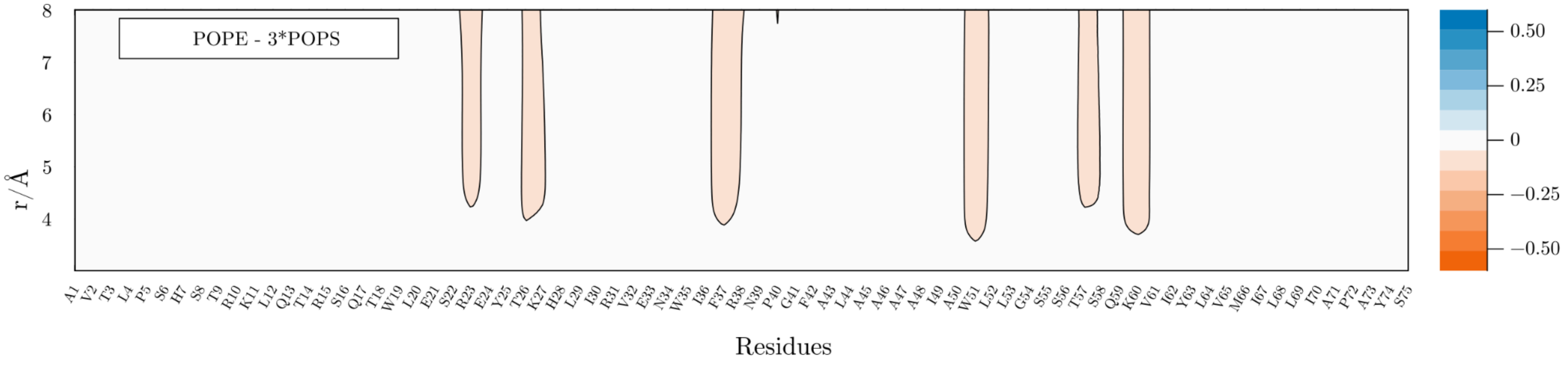

Figure S17. Weighted density maps of coordination number differences for POPE and POPS interactions with chain N.

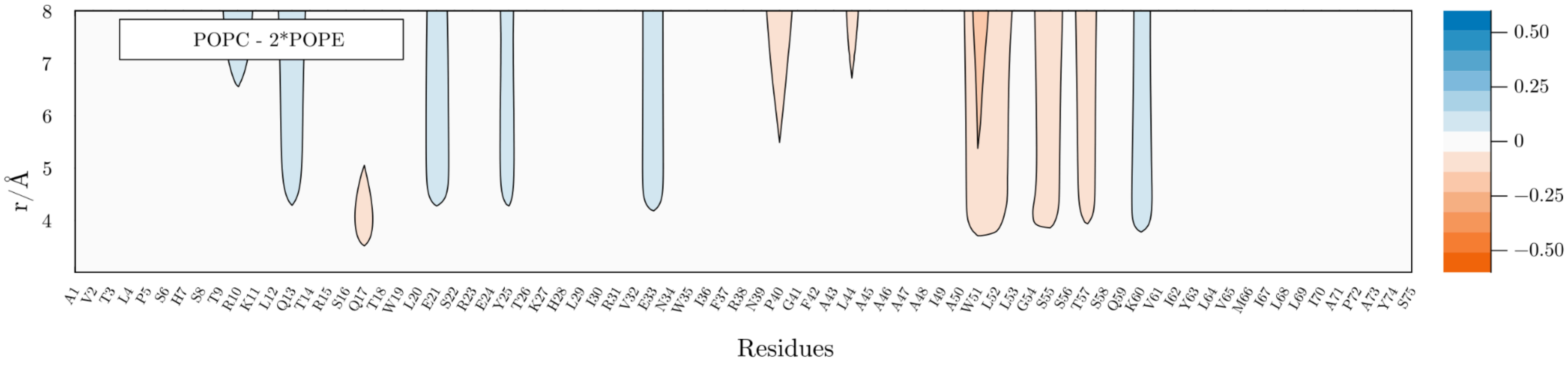

Figure S18. Visualization of coordination differences between POPC and POPE, displayed as 2D density maps for chain P.

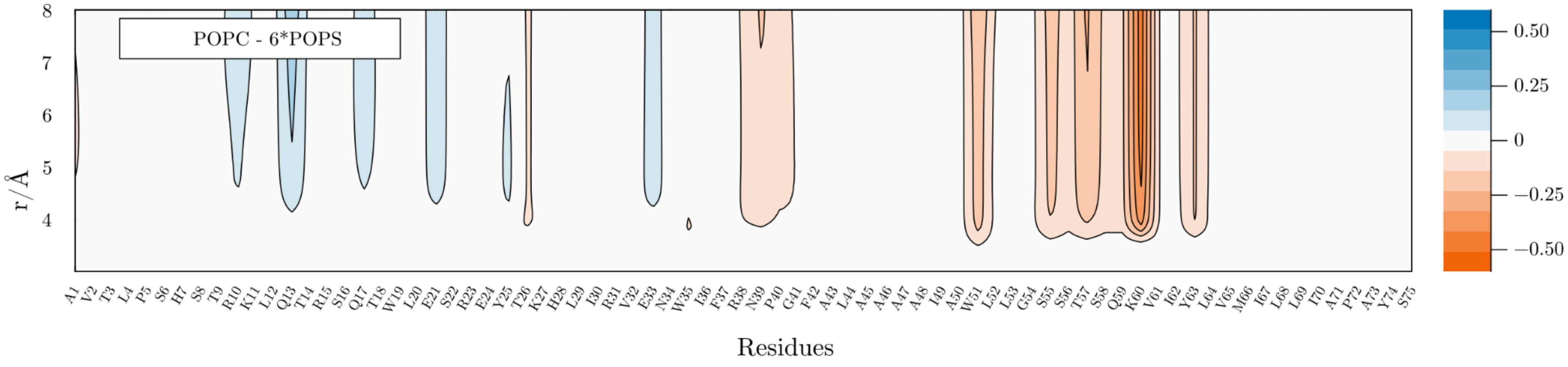

Figure S19. Two-dimensional density mapping of the relative coordination numbers of POPC versus POPS at chain P.

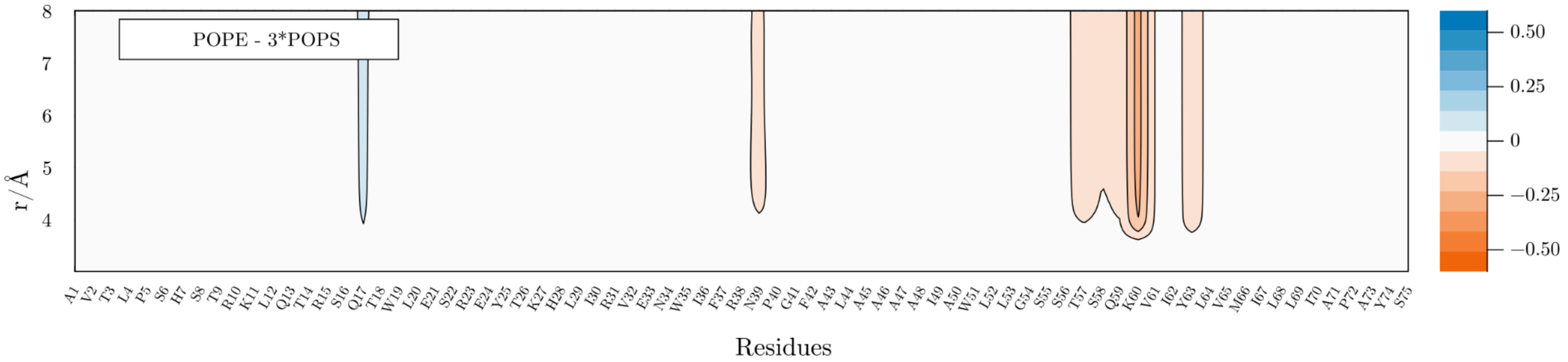

Figure S20. Weighted 2D density plots revealing coordination differences between POPE and POPS with chain P.

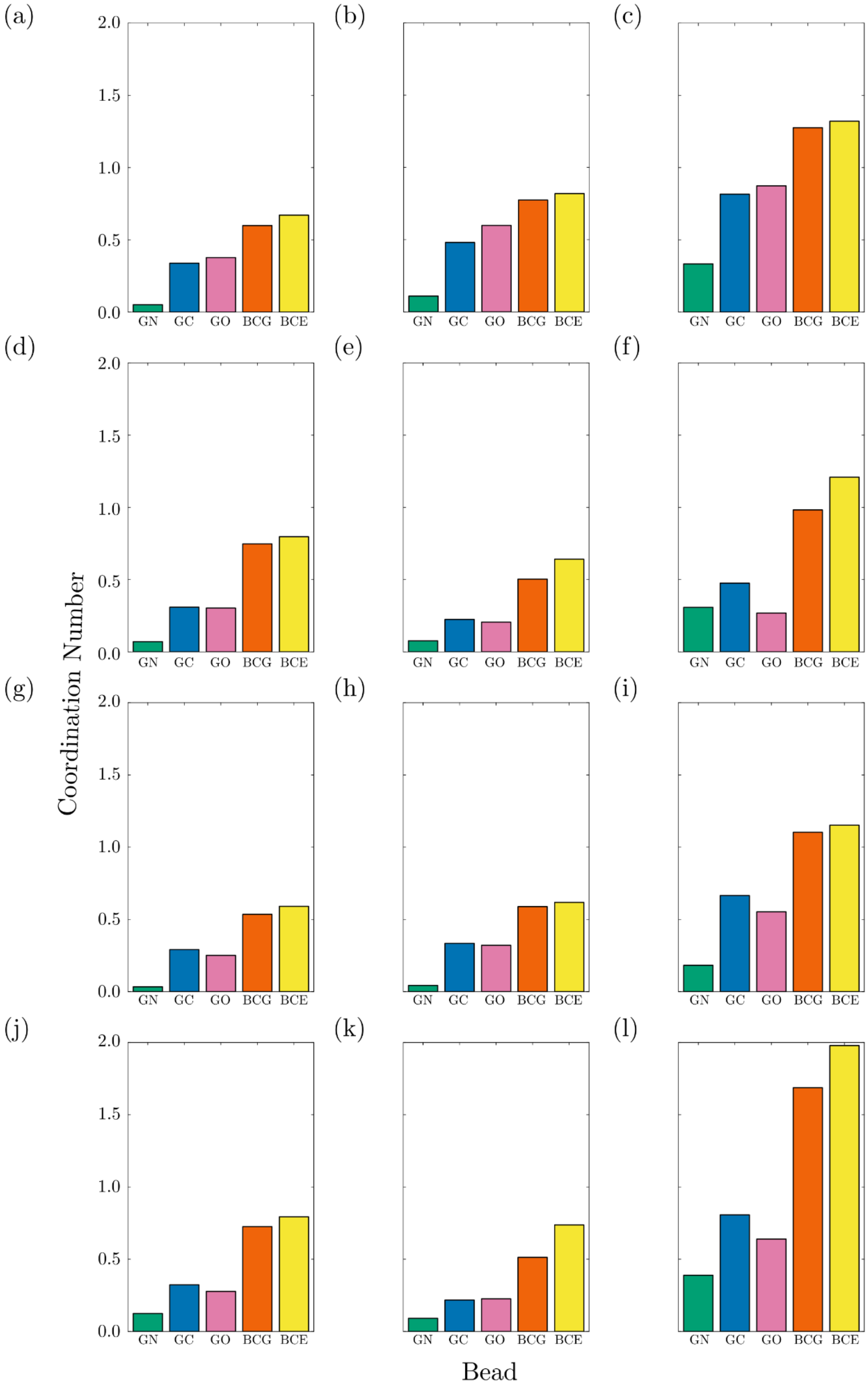

Figure S21. Normalized lipid contributions around lysine residues: (a–c) Lys480 of chain M with POPC, POPE, and POPS, respectively; (d–f) Lys480 of chain O with POPC, POPE, and POPS; (g–i) Lys60 of chain N with POPC, POPE, and POPS; and (j–l) Lys60 of chain P with POPC, POPE, and POPS.

Selective Lipid Interactions at Protein–Membrane Interfaces - Part I

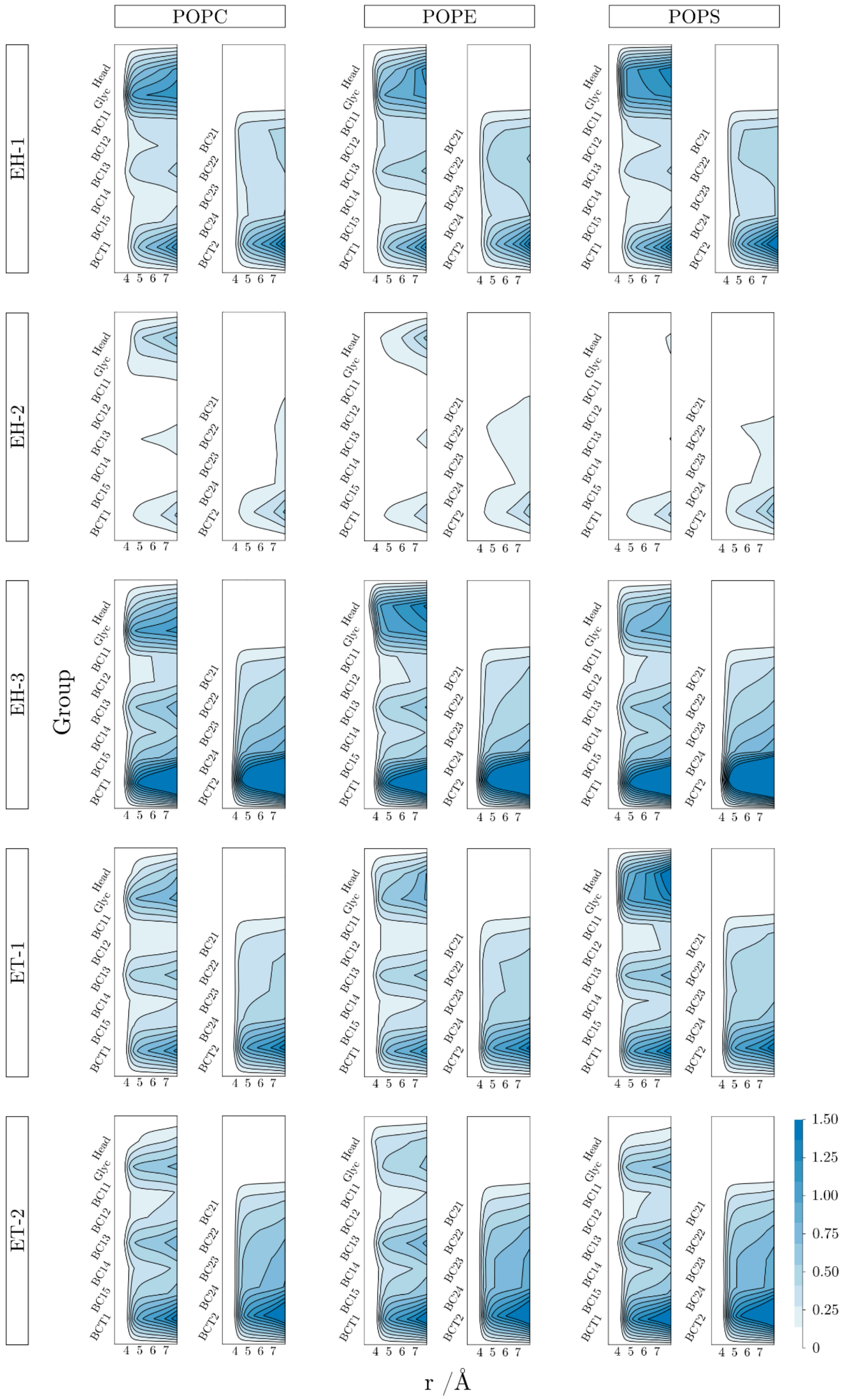

Figure S22. 2D density maps of lipid bead contributions from POPC, POPE, and POPS across the EH, ET, and MH subunits of chain K.

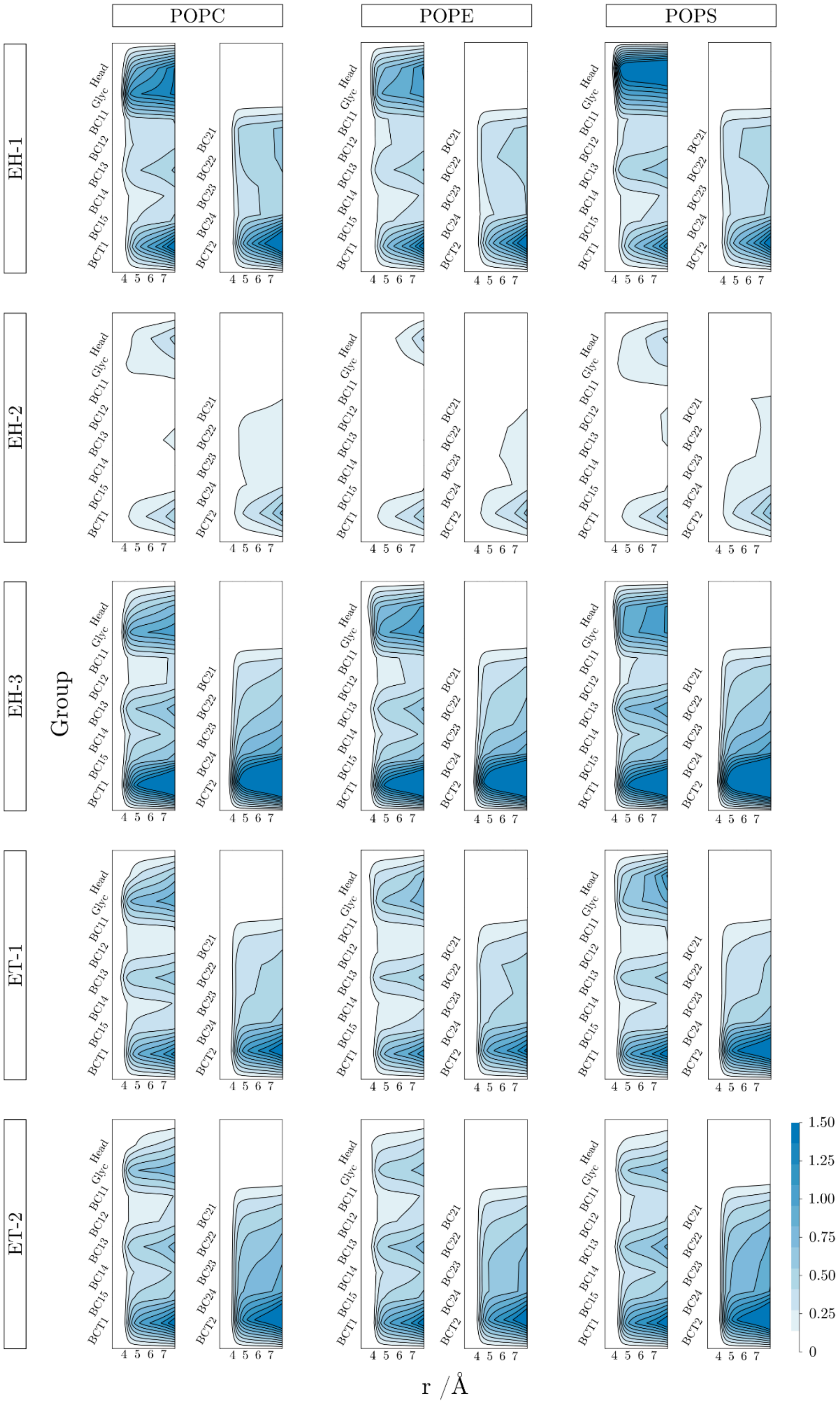

Figure S23. Comparative 2D density maps showing how POPC, POPE, and POPS beads distribute around chain M subunits EH-1–3, ET-1–2, and MH-1–3.

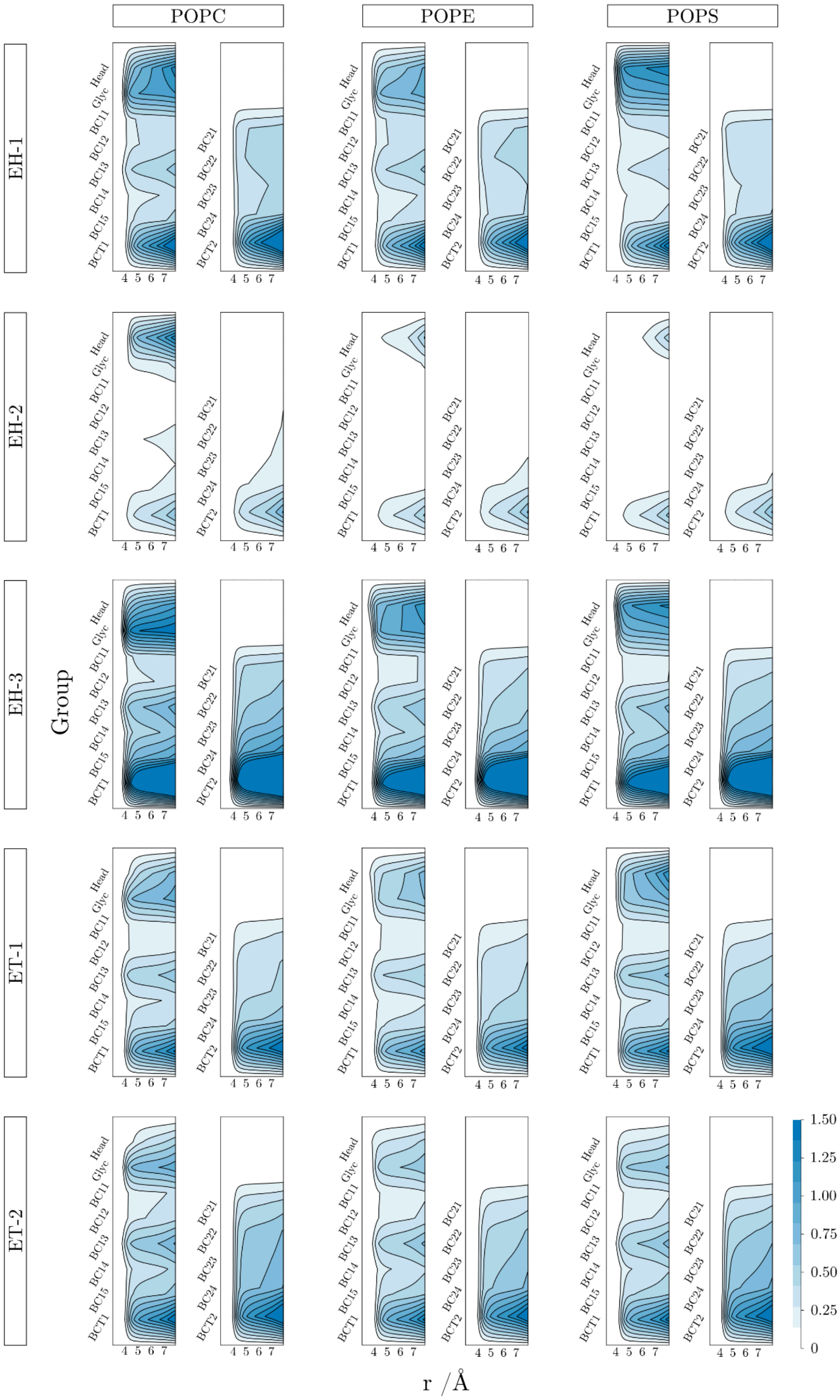

Figure S24. 2D projections of lipid bead density reveal the spatial organization of POPC, POPE, and POPS interactions with EH, ET, and MH subunits in the chains of type O.

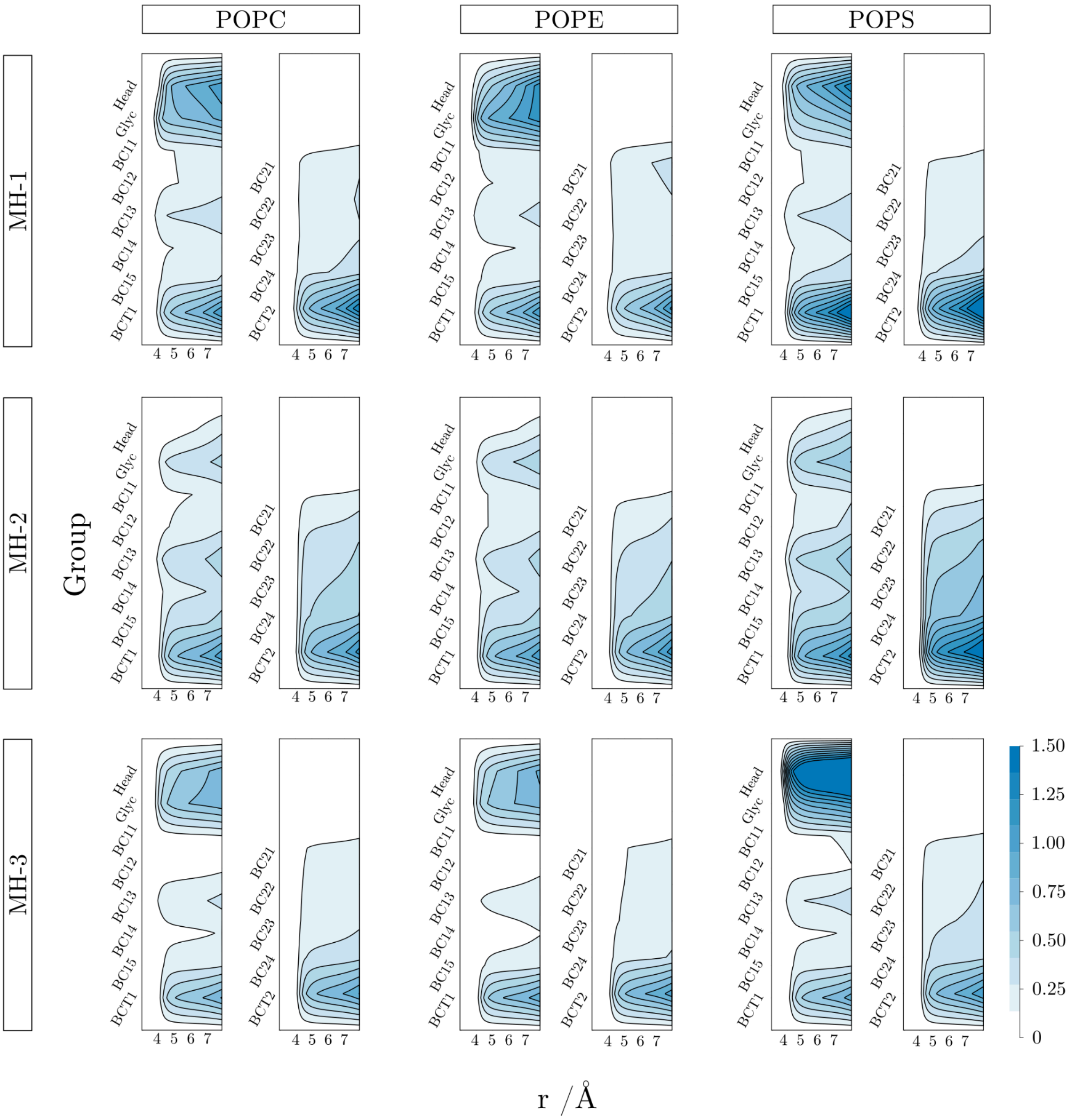

Figure S25. Spatial density maps (2D) illustrating the specific contributions of POPC, POPE, and POPS beads to the lipid environment of EH, ET, and MH subunits in the chain L.

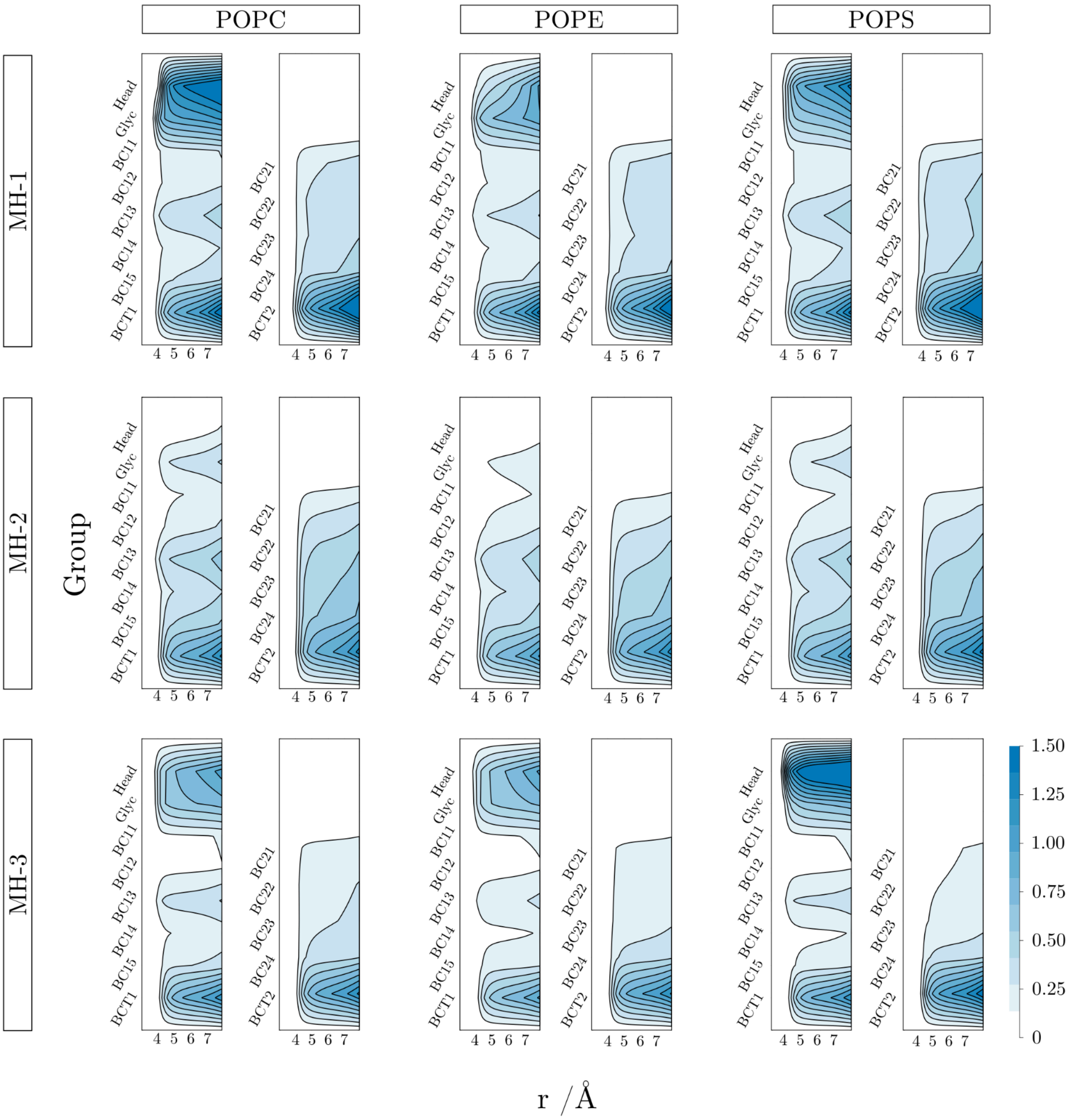

Figure S26. 2D density plots depicting the localization of lipid beads from POPC, POPE, and POPS in relation to chains N EH-1–3, ET-1–2, and MH-1–3 subunits.

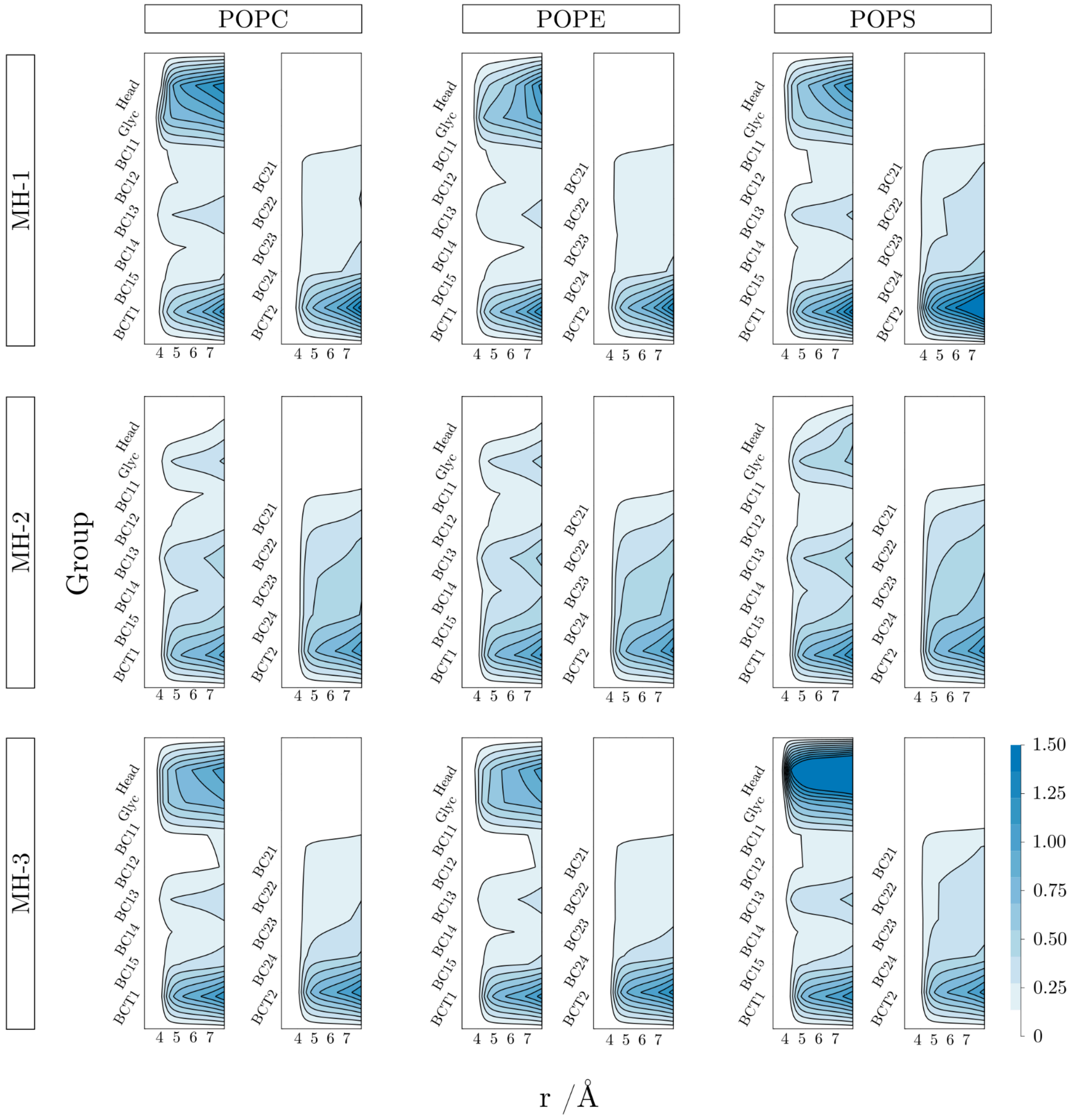

Figure S27. Mapping of lipid bead contributions (POPC, POPE, and POPS) as 2D densities highlights distinct interaction patterns across EH, ET, and MH subunits in chain P.

Selective Lipid Interactions at Protein–Membrane Interfaces - Part II

EH-1, EH-2, and EH-3 (Figures S39-37a, b, and c) are amphipathic helices positioned close to the membrane surface, and their composition suggests distinct modes of association to the viral membrane. EH-1 contains one positively charged lysine and several hydrophobic residues (Phe, Thr, and Tyr) showing significant interactions with the membrane, consistently with a surface-bound amphipathic helix stabilized by complementary hydrophobic–polar interactions. EH-2 is remarkable for containing a single tryptophan that interacts with the lipids. The indole moiety of Trp is well suited to engage in hydrogen bonding via the pyrrole NH. EH-3, while also amphipathic, contains a larger proportion of hydrophobic residues (Phe, Leu, Ile, and His), together with neutral polar residues (Asn and Gln) interacting with the lipid bilayer. This dual composition likely supports surface association, with lipid binding dominated by aromatic Phe residues. In all three EH helices, POC interactions appear consistent with its neutral but bulky polar head, which can engage both hydrophobic faces of amphipathic helices and polar residues exposed at the surface.

The ET helices adopt a transmembrane orientation, favoring extensive contacts with hydrophobic residues, as shown in Figures S29-37d and e. ET-1 is stabilized by two tryptophans at the interface, acting as orientational anchors through interactions with headgroups and the glycerol backbone. Additional contributions from Phe within the aromatic belt strengthen this interfacial positioning, while deeper hydrophobic contacts involve Thr, Met, Leu, and Ile. ET-2 also displays transmembrane character, with interactions dominated by hydrophobic residues (Cys, Leu, Phe, and Gly). The presence of serine, a neutral polar residue, at the interface suggests possible stabilization through headgroup contacts.

The MH helices exhibit complementary contributions from both aromatic and hydrophobic residues (Figures S29-37f, g, and h). Residues Tyr, Trp, His, and Phe, in the MH-1, interact considerably with the lipid bilayer. This suggests a cooperative role of aromatic belt residues and electrostatic contributions in orienting MH-1. MH-2 is more deeply embedded, dominated by hydrophobic interactions (Leu, Pro, Phe, Gly, Ile, and Ala). Here, Phe residues may still play a role at the interfacial boundary. MH-3 also behaves as a transmembrane segment, with hydrophobic residues (Thr, Tyr, Val, and Ile) providing the main contribution, while polar residues Ser and Gln establish complementary interactions with headgroups.

EH-1 and EH-3 are both amphipathic α-helices with moderate hydrophobicity (0.32 and 0.58, respectively) and relatively high hydrophobic moments, suggesting favorable interactions with the interfacial region of the bilayer. These characteristics are consistent with their roles in membrane association without full bilayer penetration. Coordination with both headgroup and glycerol beads from the membrane was observed in these regions, particularly in EH-3, which displayed strong and spatially organized lipid interactions. The amphipathic nature of EH-1 and EH-3 enables these helices to align parallel to the bilayer surface, as supported by their horizontal orientation and their distribution of polar and nonpolar residues, facilitating both hydrophobic and electrostatic contacts with the lipid interface. This is in agreement with studies showing that surface-bound amphipathic helices contribute to viral protein reorganization during membrane fusion and particle maturation.

EH-2, also amphipathic, exhibits unique interfacial behavior. This helix is characterized by a dominant tryptophan residue (Trp429), which appears to act as a membrane anchor. The preference for interaction with lipid headgroups in this region (particularly from POPE and POPC) is consistent with previous findings that aromatic

residues such as tryptophan and tyrosine are frequently enriched at membrane interfaces due to their ability to form hydrogen bonds and cation–π interactions with phosphate or choline groups[1]. The spatial localization of Trp429 at the interface may confer structural stabilization at the bilayer boundary, possibly influencing conformational transitions in the viral envelope during fusion.

ET-1 and ET-2 are strongly hydrophobic helices (hydrophobicity > 1.1) with low hydrophobic moments, indicating uniform distribution of nonpolar residues and suggesting full transmembrane insertion. These helices display robust coordination with the hydrophobic tail beads of POPC and POPE, in line with their vertical orientation within the membrane. Residues such as tryptophan, phenylalanine, and methionine contribute significantly to lipid interactions in these domains. This is consistent with structural studies reporting that aromatic residues at the midplane or termini of TM helices stabilize bilayer integration by reducing local bilayer perturbation and promoting favorable lipid packing[2].

The MH-1 segment of the M protein is characterized by a high hydrophobic moment and a balanced proportion of polar and nonpolar residues. The helical wheels (Figures S29-37f) show a distribution of side chains compatible with interfacial alignment, and residues such as Tyr25, His28, Trp35, and Phe37 dominate its lipid interaction profile. These features suggest that MH-1 acts similarly to an amphipathic helix aligned parallel to the membrane surface, where it engages primarily with lipid headgroups and the glycerol backbone. This interpretation is supported by prior cryo-EM studies of flaviviral membrane proteins, which describe MH-1 as participating in conformational rearrangements known to occur on fusion[3].

In contrast, MH-2 and MH-3 (Figures S29-37g, and h) show lower hydrophobic moments and higher nonpolar residue content (Val, Phe), in accordance with their

transmembrane configurations. Their lipid interactions are predominantly mediated through tail beads, again consistent with a deeper insertion into the bilayer interior.

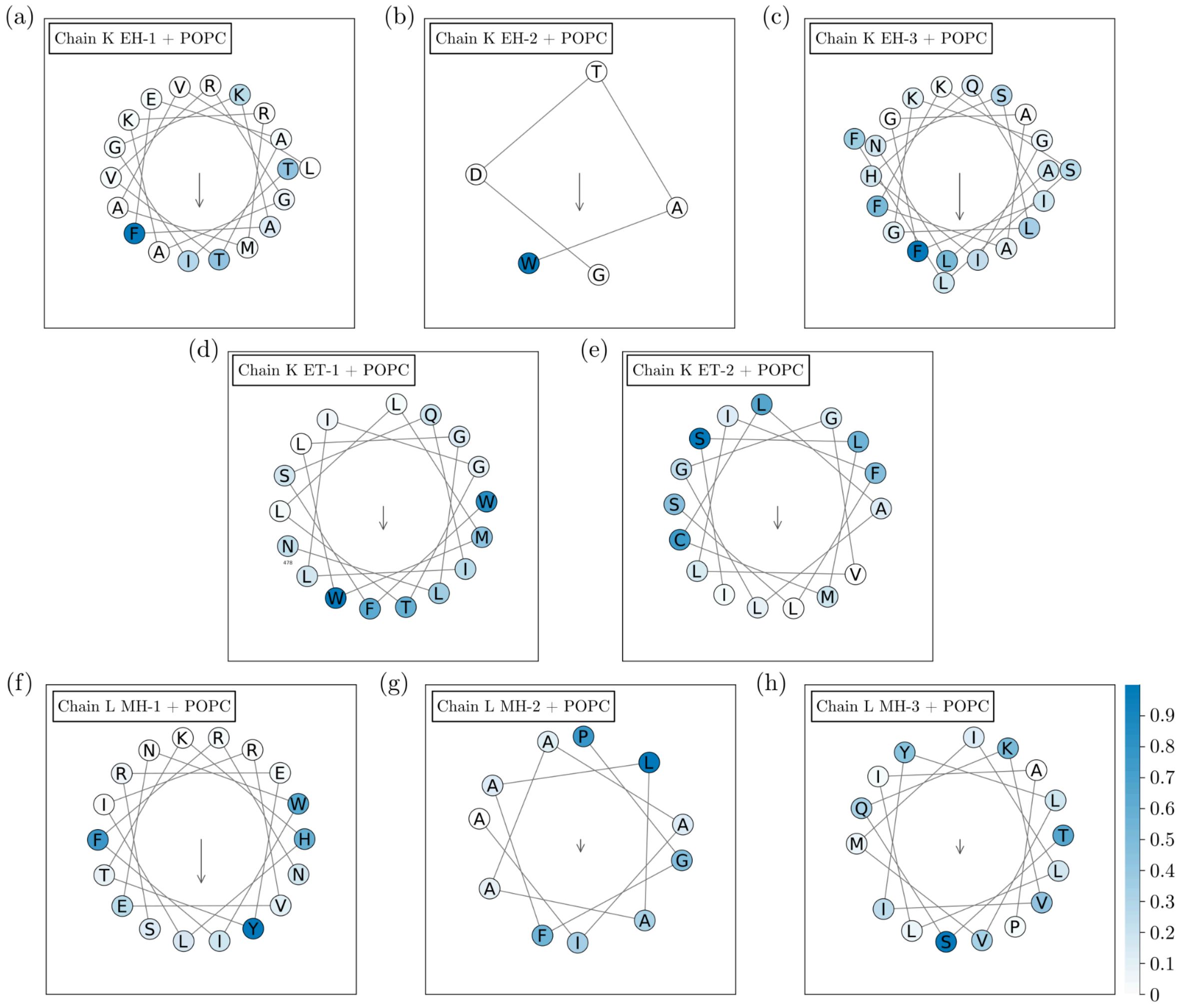

Figure S28. Helical wheel representations of the subunits of chains of type K and L, illustrating POPC contribution at 5 Å, The arrows indicate the hydrophobic moment.

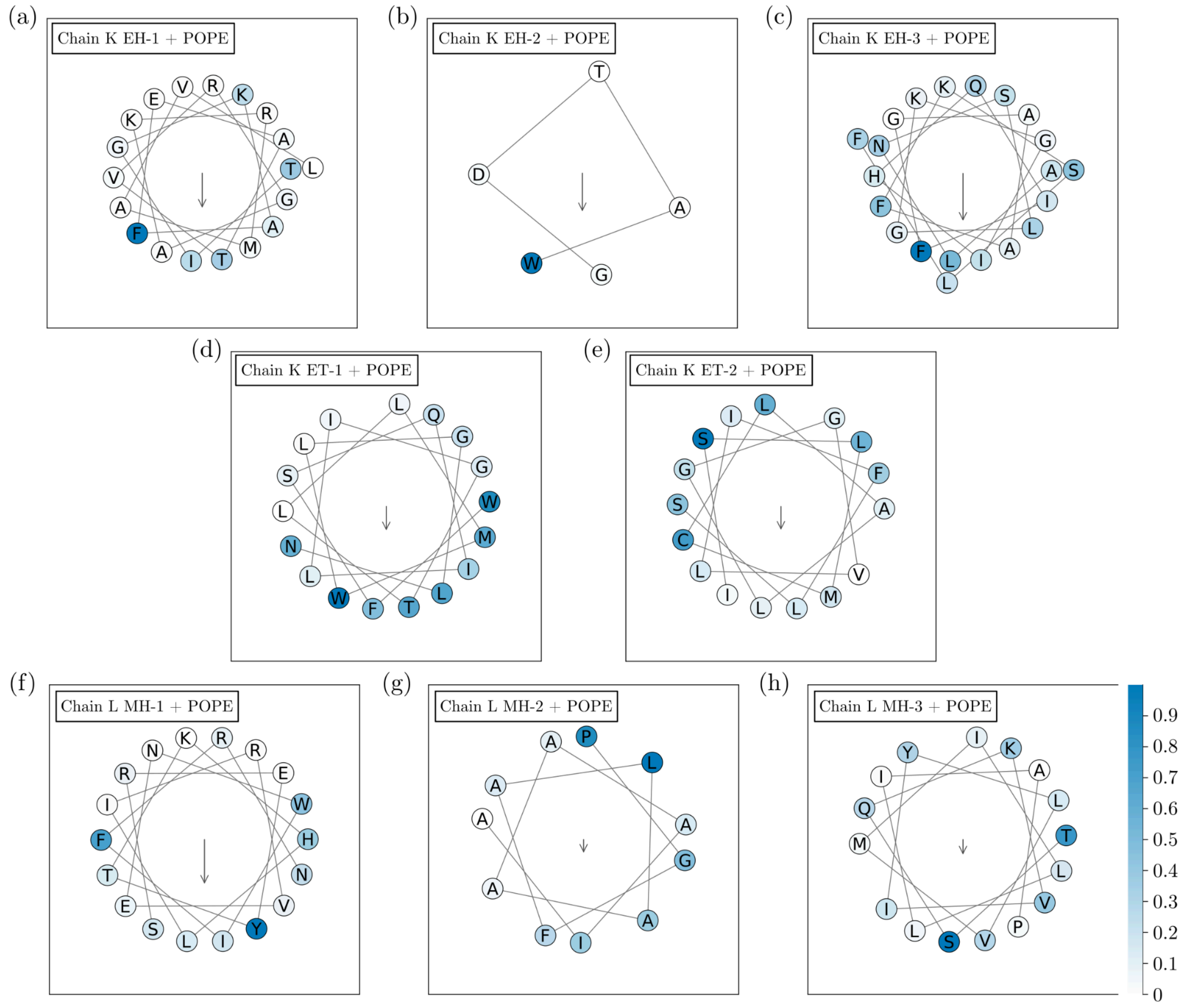

Figure S29. Helical wheel diagrams of type K and L subunits, highlighting POPE contacts per residue within 5 Å.

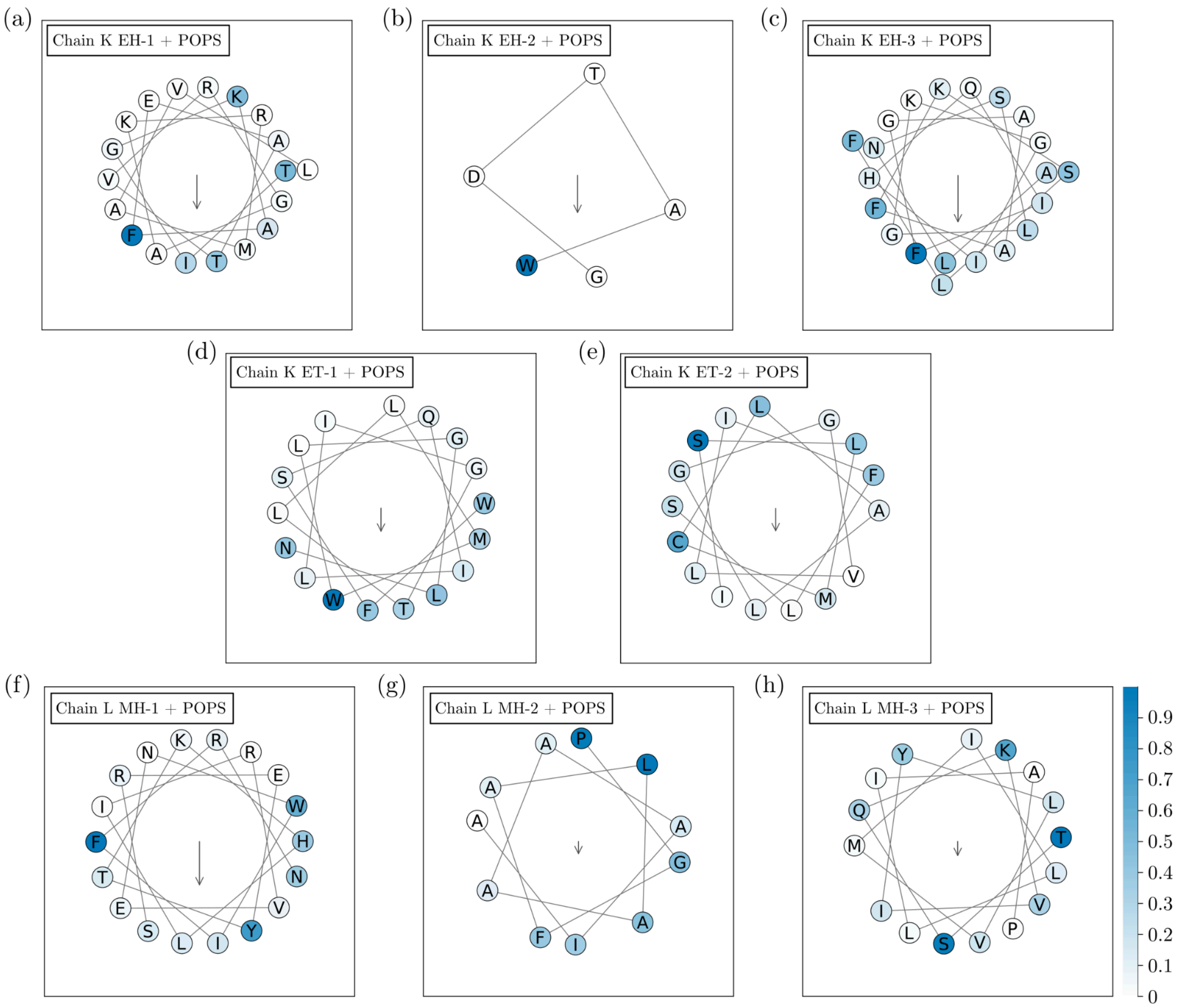

Figure S30. Helical wheel plots of chains K and L, where POPS contributions at 5 Å are shown.

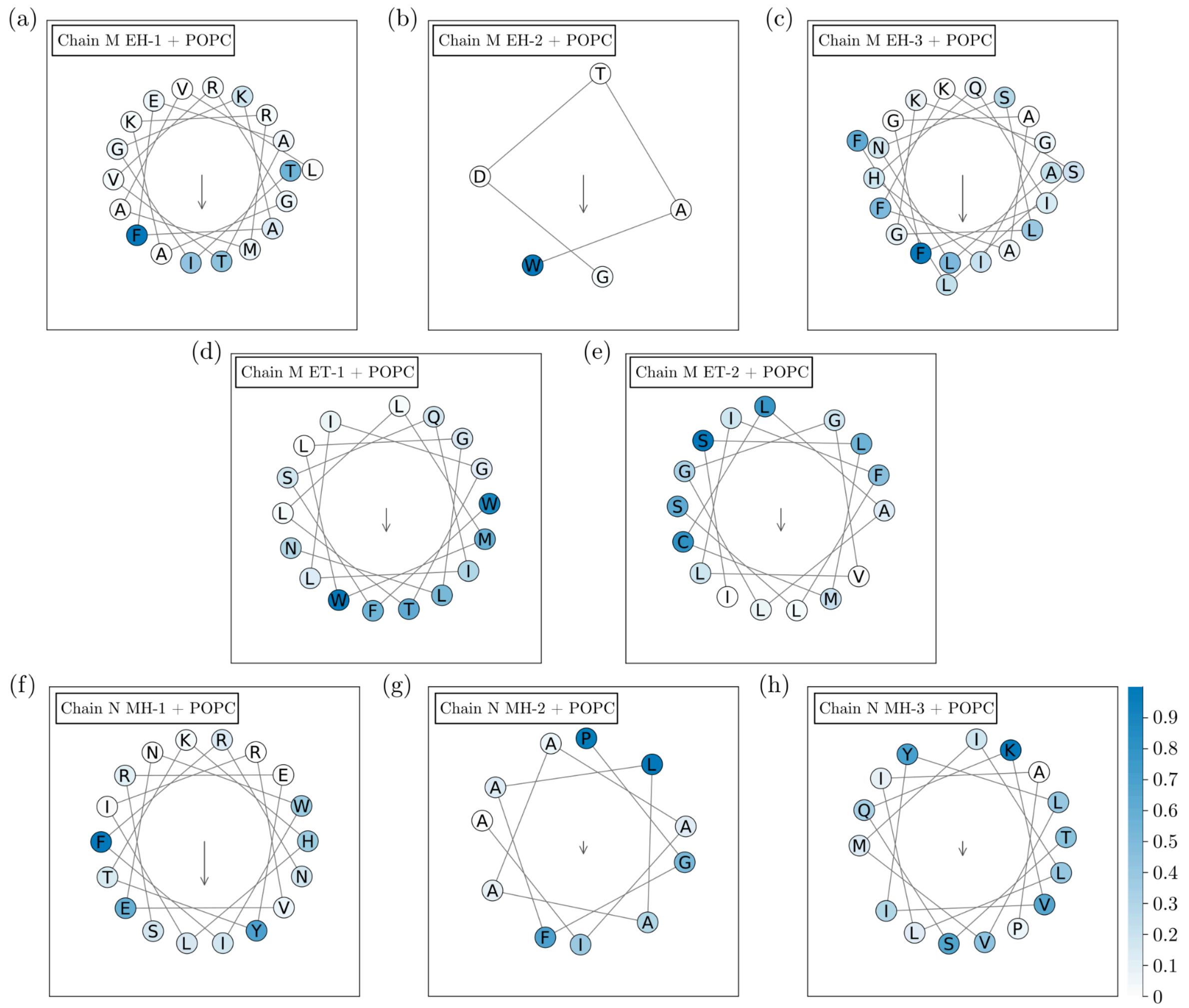

Figure S31. Helical wheel representations of chains M and N, showing that POPC interactions at 5 Å around residues aligned with the hydrophobic moment.

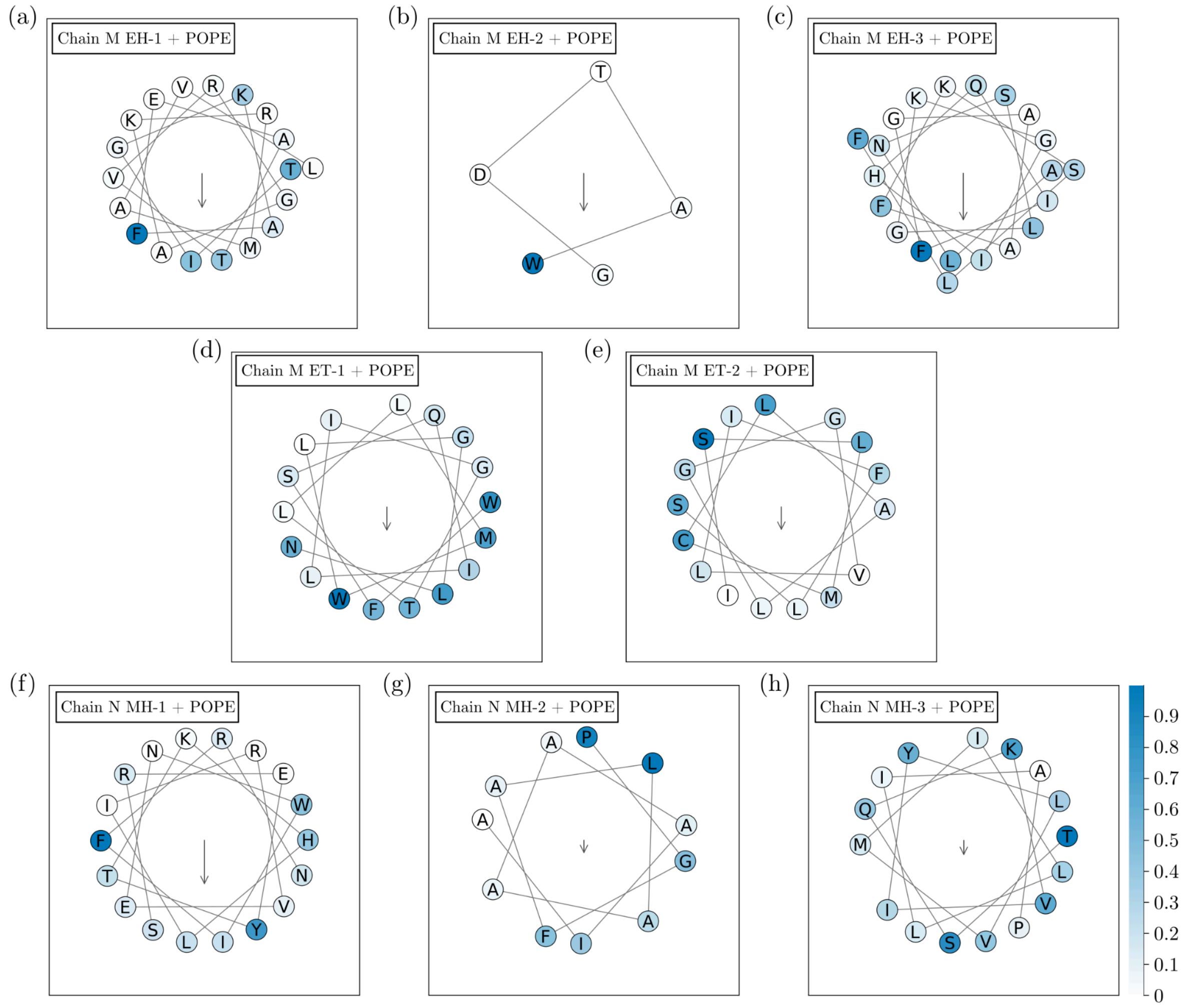

Figure S32. POPE contacts mapped onto helical wheels of type M and N subunits at 5 Å, revealing the orientation of the hydrophobic moment.

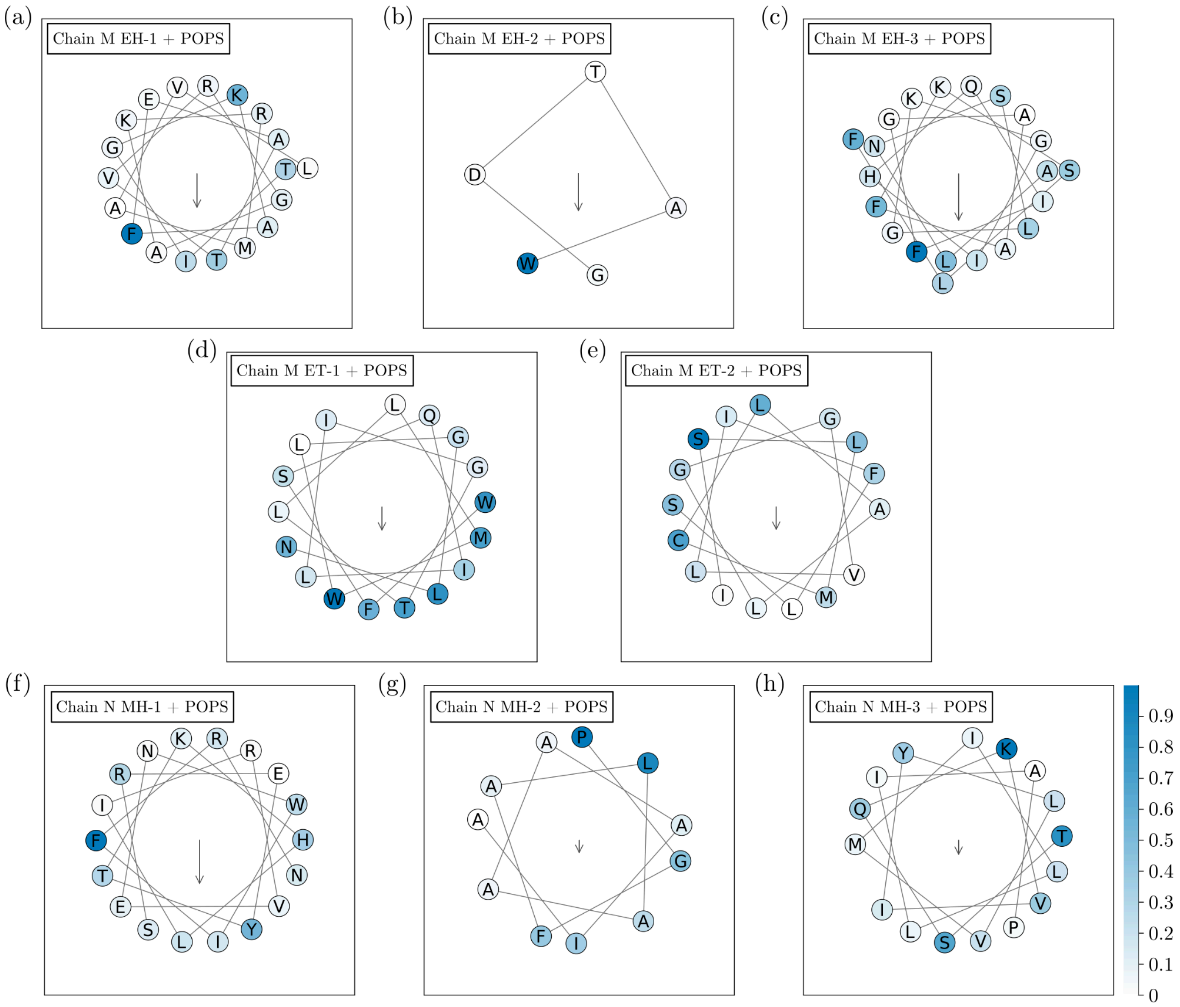

Figure S33. Helical wheel of chains M and N with POPS contacts at 5 Å.

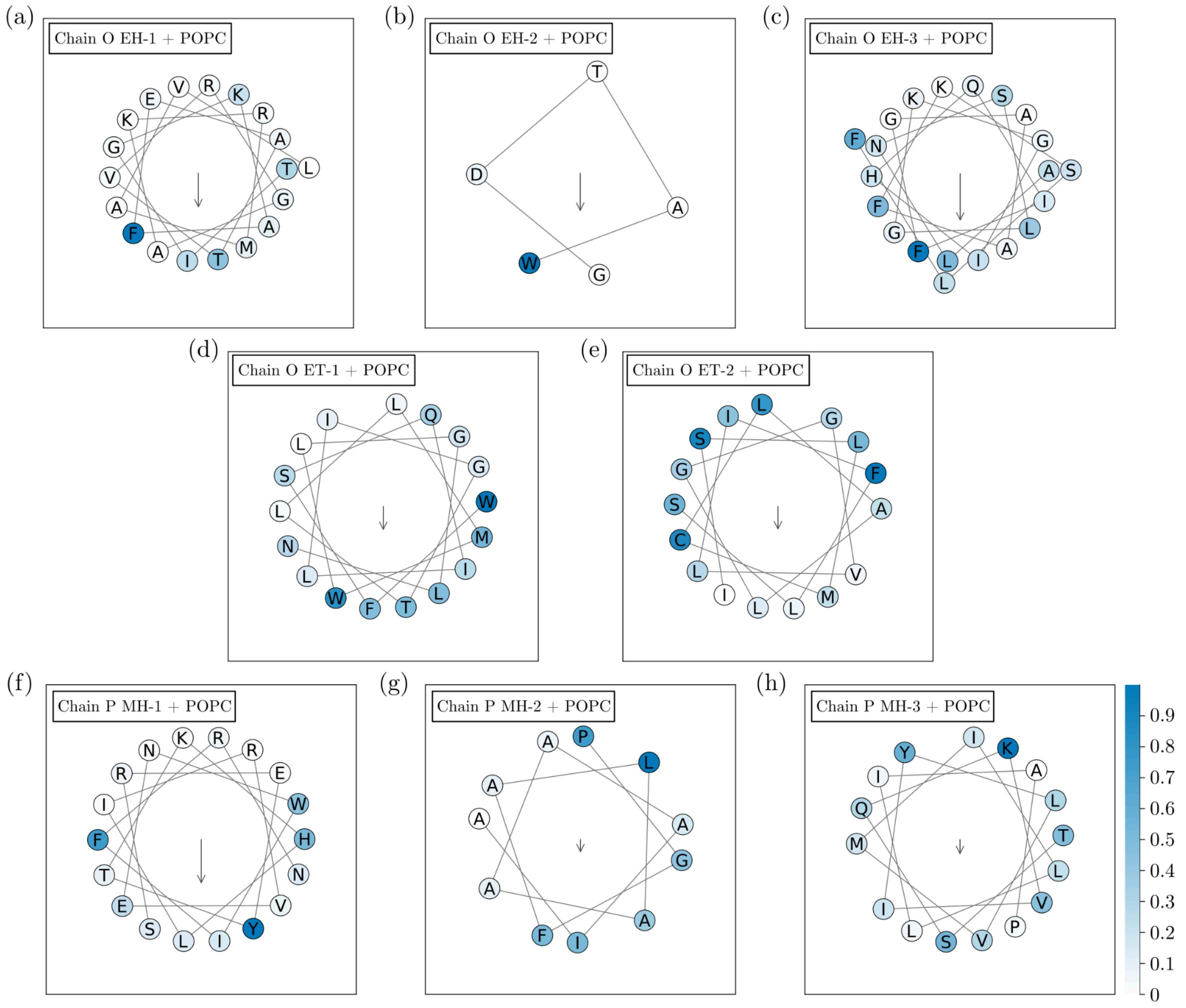

Figure S34. Helical wheels of chains O and P depicting POPC contributions within 5 Å of the subunits.

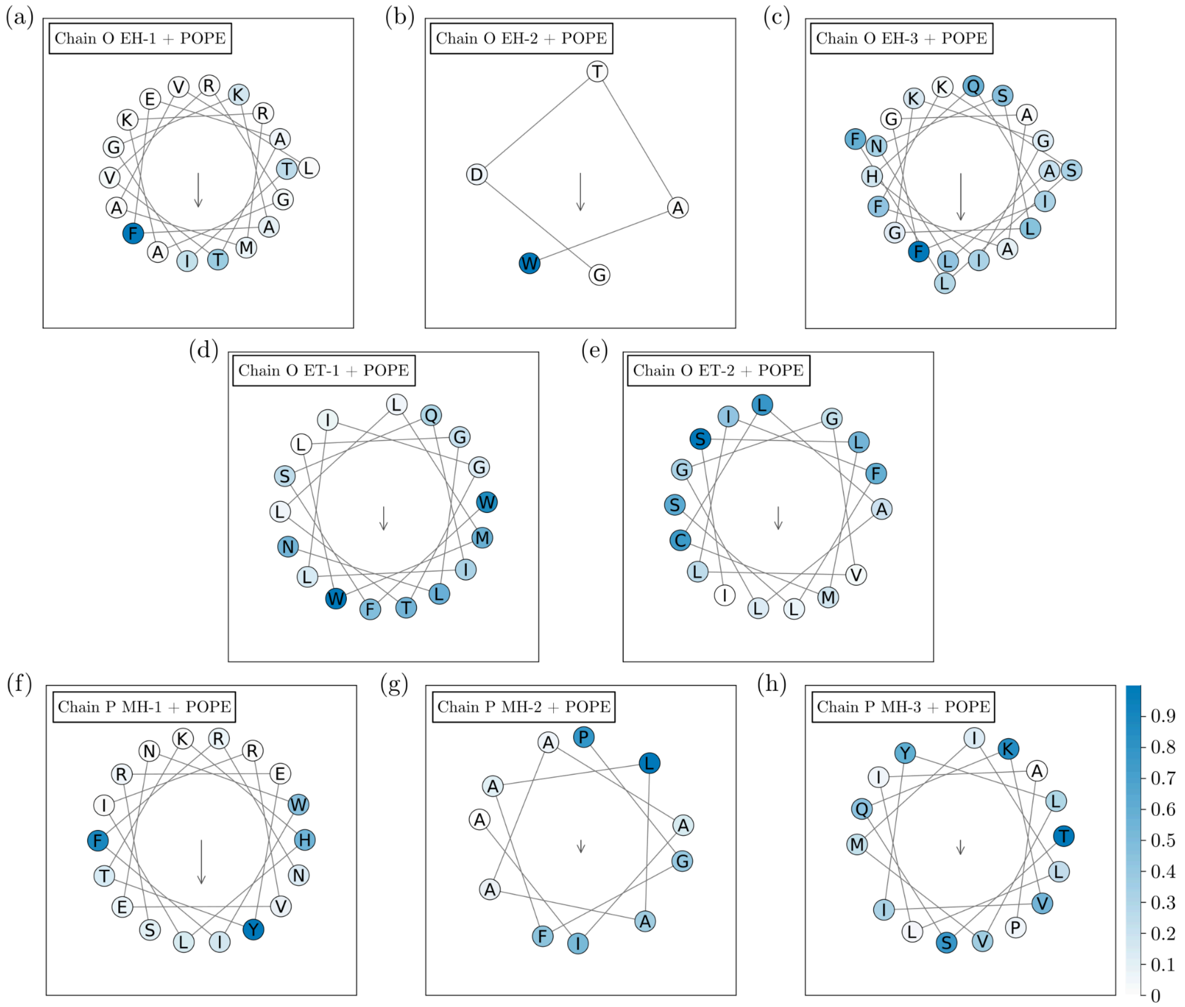

Figure S35. Helical wheel diagrams of subunits O and P. POPE contributions are mapped at 5 Å.

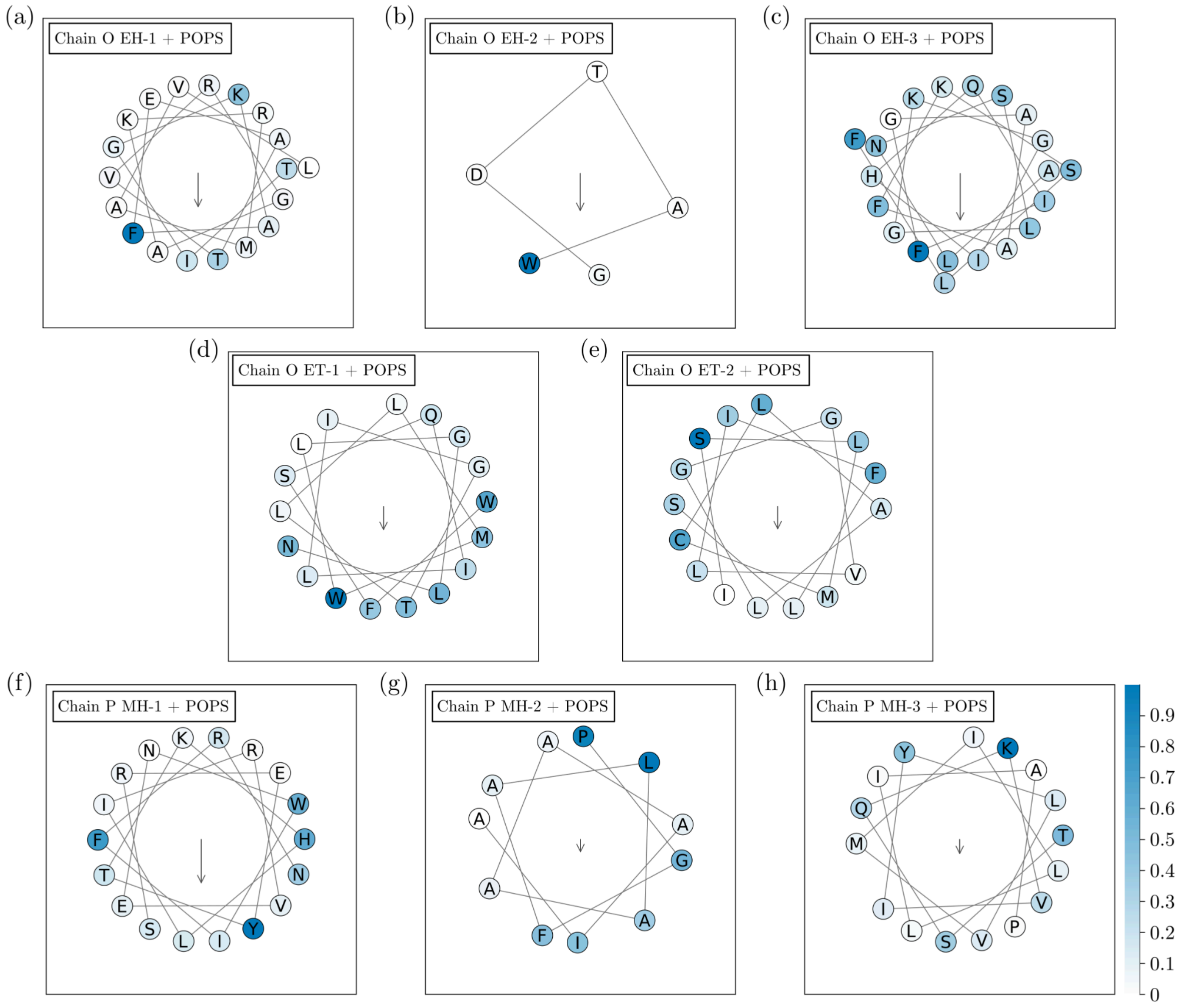

Figure S36. Helical wheels of chains O and P depicting POPS contributions within 5 Å, with arrows indicating hydrophobic moment vectors relevant for membrane embedding.

Additional Information

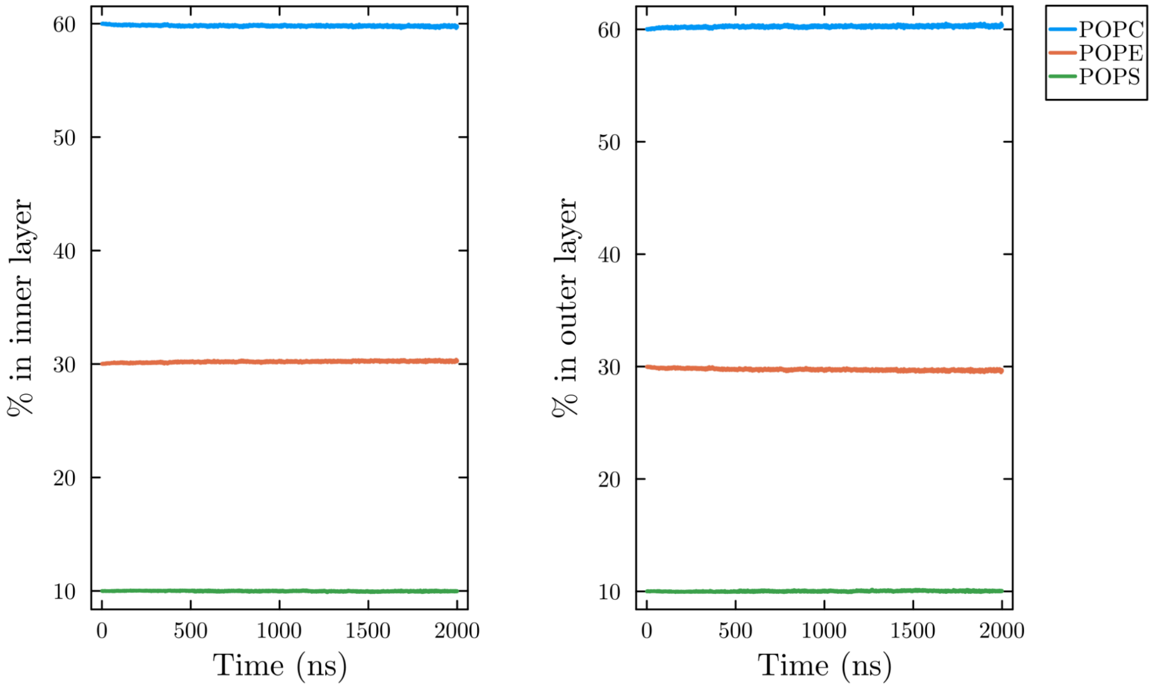

Figure S37. Time series of the relative fractions of POPC, POPE, and POPS in the inner (left) and outer (right) leaflets over the 2 μs coarse-grained simulation. Lipids were classified based on the position of a representative headgroup bead relative to the virion center. The compositions remain essentially constant at the imposed 6:3:1 ratio, with only small fluctuations around the mean, indicating that transbilayer lipid exchange does not lead to net compositional changes on the simulated timescale.

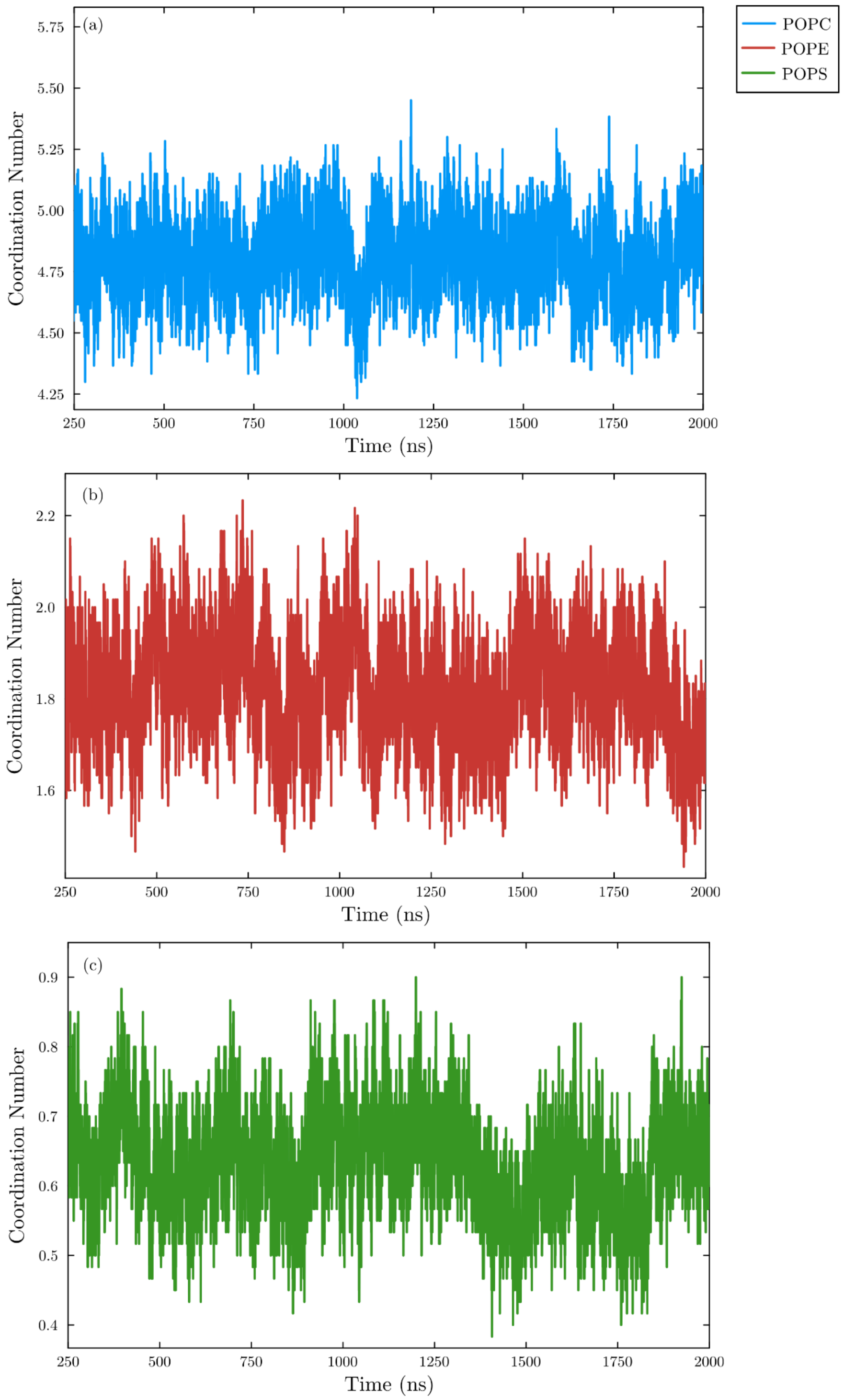

Figure S38. Coordination number as a function of time per residue W429, shown separately for contacts with POPC (a), POPE (b), and POPS (c). This residue is presented as a representative example to illustrate the equilibration of lipid–protein contacts. The absence of systematic drifts and the presence of stationary fluctuations indicate that the system is well equilibrated over the analyzed time window.

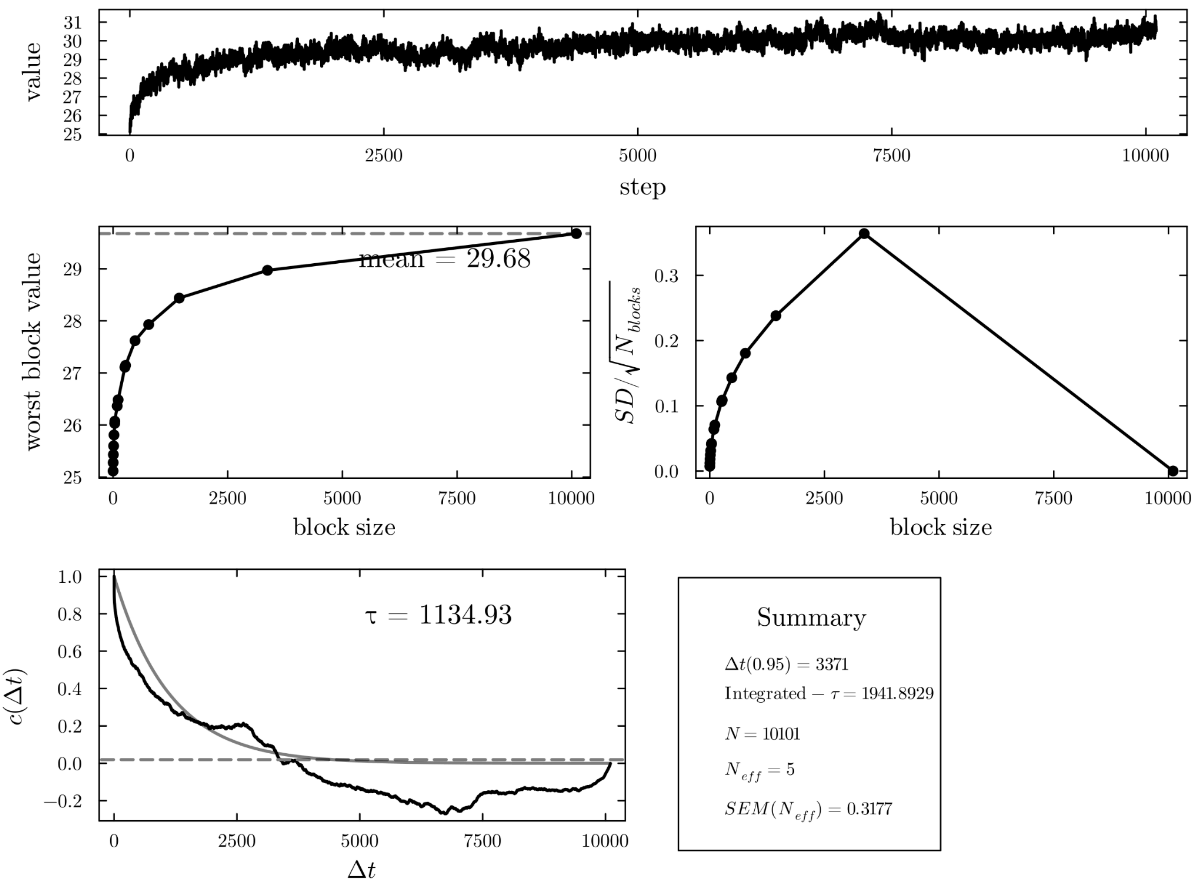

Figure S39. Average coordination numbers of asymmetric units of the virus shell relative inner-monolayer POPC at 5 Å along the simulation and block analysis for time-correlated data, showing the convergence of the worst block value to the global mean, the dependence of the standard error on the block size, the autocorrelation function with integrated correlation time, and the effective number of independent samples, calculated with the block-averages method of MolSimToolkit.jl. The estimated error in the mean is the standard error computed considering the effective number of samples.

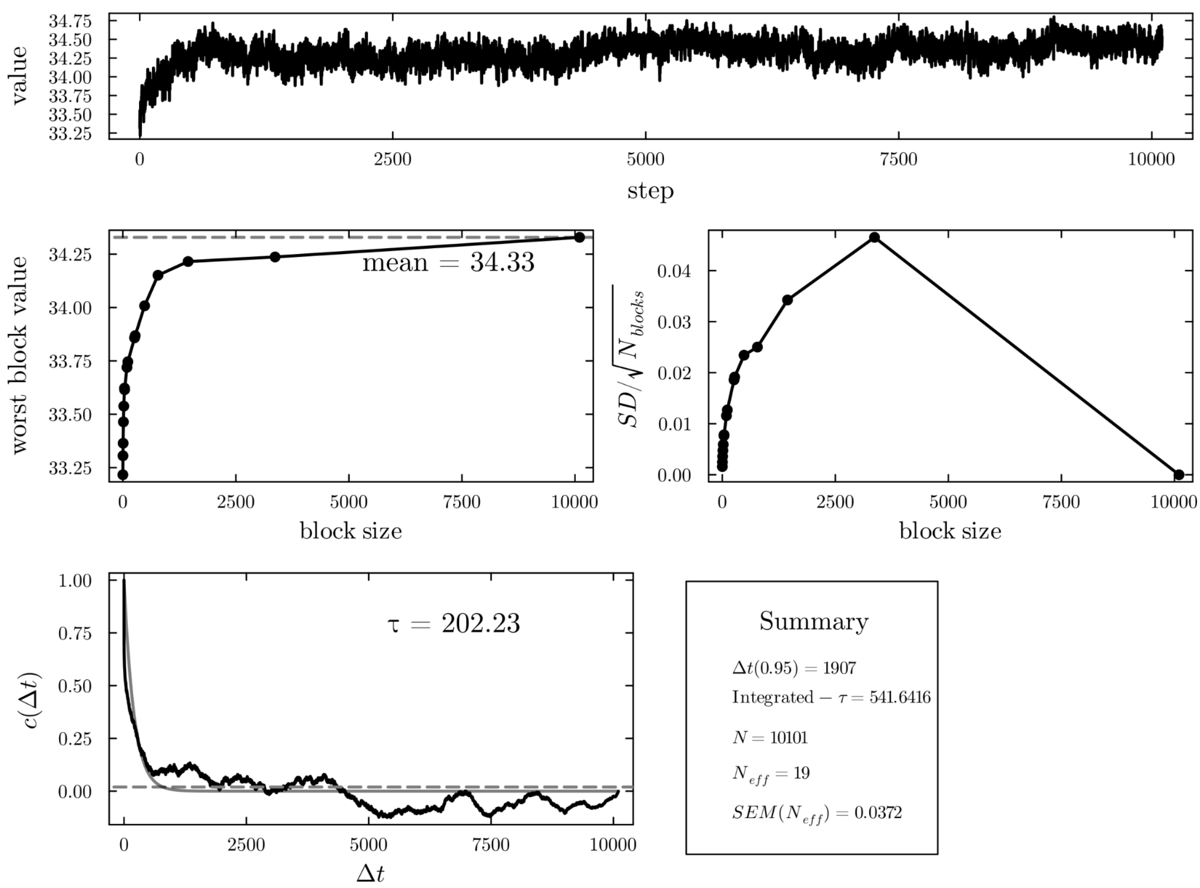

Figure S40. Average coordination numbers of asymmetric units of the virus shell relative outer-monolayer POPC at 5Å along the simulation and block analysis for time-correlated data, showing the convergence of the worst block value to the global mean, the dependence of the standard error on the block size, the autocorrelation function with integrated correlation time, and the effective number of independent samples, calculated with the block-averages method of MolSimToolkit.jl. The estimated error in the mean is the standard error computed considering the effective number of samples.

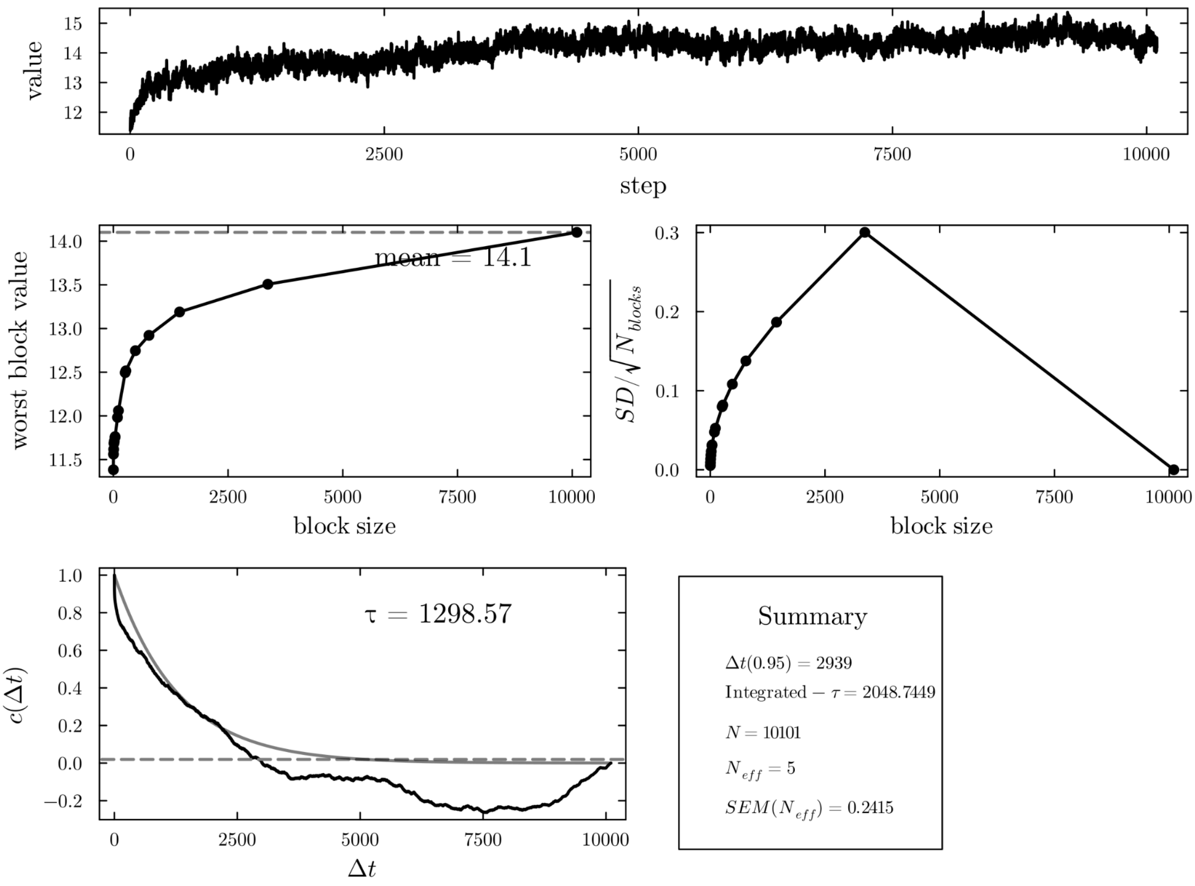

Figure S41. Average coordination numbers of asymmetric units of the virus shell relative inner-monolayer POPE at 5 Å along the simulation and block analysis for time-correlated data, showing the convergence of the worst block value to the global mean, the dependence of the standard error on the block size, the autocorrelation function with integrated correlation time, and the effective number of independent samples, calculated with the block-averages method of MolSimToolkit.jl. The estimated error in the mean is the standard error computed considering the effective number of samples.

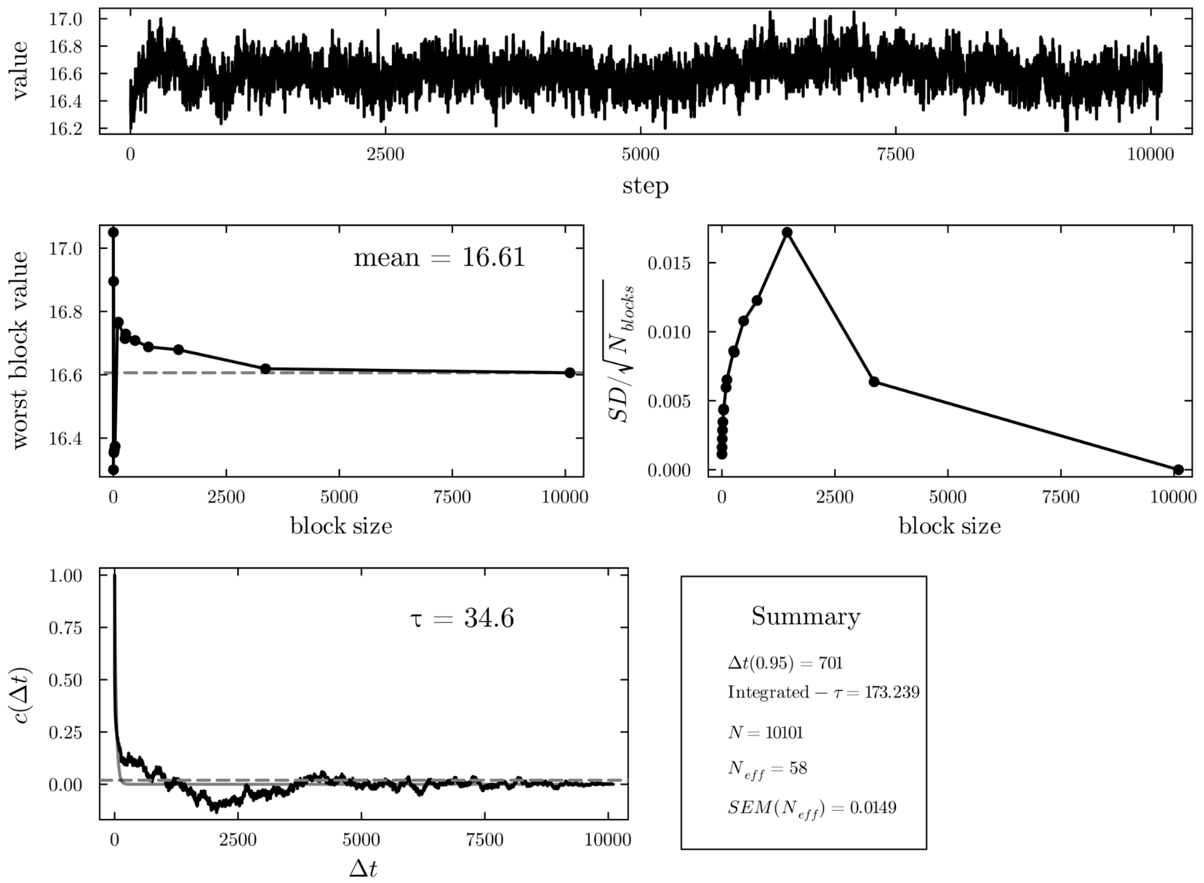

Figure S42. Average coordination numbers of asymmetric units of the virus shell relative outer-monolayer POPE at 5 Å along the simulation and block analysis for time-correlated data, showing the convergence of the worst block value to the global mean, the dependence of the standard error on the block size, the autocorrelation function with integrated correlation time, and the effective number of independent samples, calculated with the block-averages method of MolSimToolkit.jl. The estimated error in the mean is the standard error computed considering the effective number of samples.

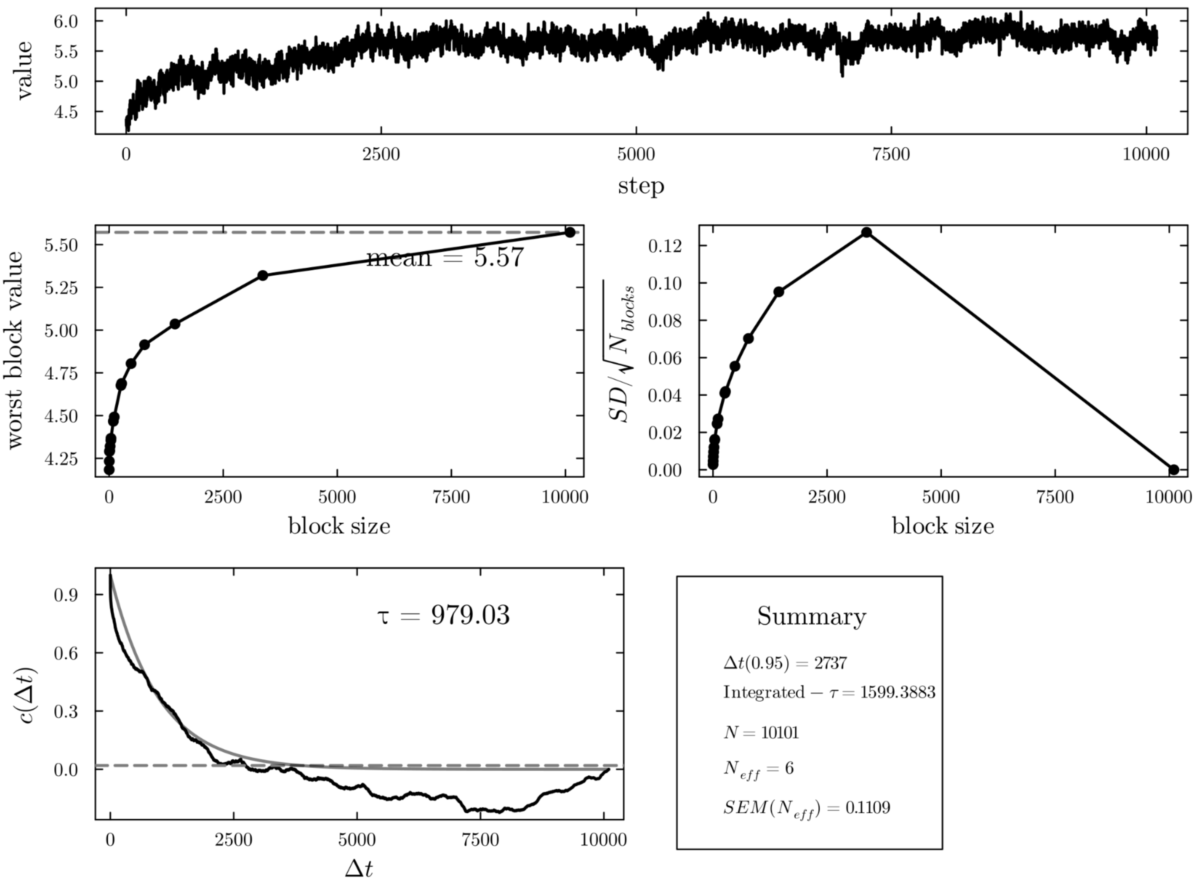

Figure S43. Average coordination numbers of asymmetric units of the virus shell relative inner-monolayer POPS at 5Å along the simulation and block analysis for time-correlated data, showing the convergence of the worst block value to the global mean, the dependence of the standard error on the block size, the autocorrelation function with integrated correlation time, and the effective number of independent samples, calculated with the block-averages method of MolSimToolkit.jl. The estimated error in the mean is the standard error computed considering the effective number of samples.

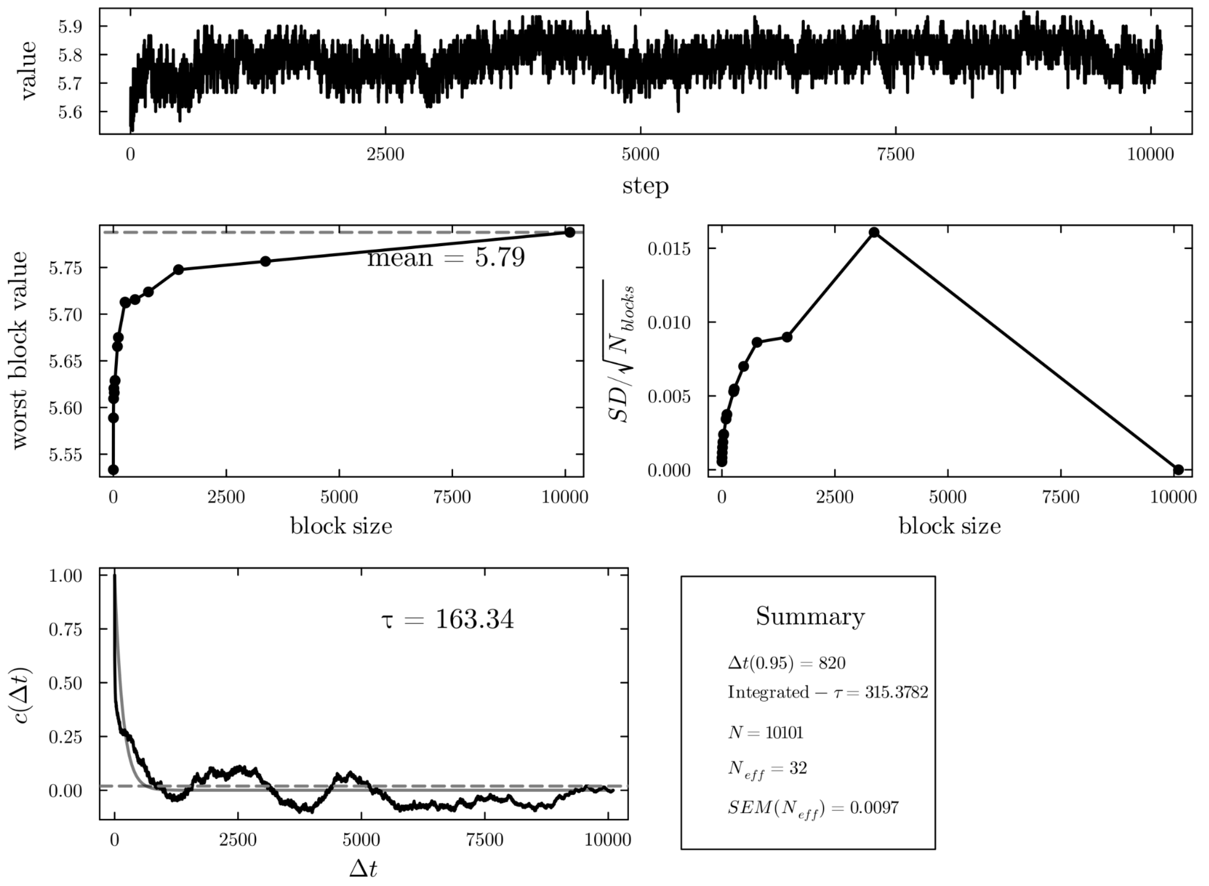

Figure S44. Average coordination numbers of asymmetric units of the virus shell relative outer-monolayer POPS at 5 Å along the simulation and block analysis for time-correlated data, showing the convergence of the worst block value to the global mean, the dependence of the standard error on the block size, the autocorrelation function with integrated correlation time, and the effective number of independent samples, calculated with the block-averages method of MolSimToolkit.jl. The estimated error in the mean is the standard error computed considering the effective number of samples.

Additional References

1. Khemaissa, S., Sagan, S. & Walrant, A. Tryptophan, an Amino-Acid Endowed with Unique Properties and Its Many Roles in Membrane Proteins. *Crystals* **11**, 1032 (2021).
2. Amino-Acid Solvation Structure in Transmembrane Helices from Molecular Dynamics Simulations. *Biophysical Journal* **91**, 4450–4463 (2006).
3. Zhang, W. *et al.* Visualization of membrane protein domains by cryo-electron microscopy of dengue virus. *Nature Structural & Molecular Biology* **10**, 907–912 (2003).
